# How learning to read Braille in visual and tactile domains reorganizes the sighted brain

**DOI:** 10.3389/fnins.2024.1297344

**Published:** 2025-01-06

**Authors:** Maciej Gaca, Alicja M. Olszewska, Dawid Droździel, Agnieszka Kulesza, Małgorzata Paplińska, Bartosz Kossowski, Katarzyna Jednoróg, Jacek Matuszewski, Aleksandra M. Herman, Artur Marchewka

**Affiliations:** ^1^Laboratory of Brain Imaging, Nencki Institute of Experimental Biology, Polish Academy of Sciences, Warsaw, Poland; ^2^The Maria Grzegorzewska University, Warsaw, Poland; ^3^Laboratory of Language Neurobiology, Nencki Institute of Experimental Biology, Polish Academy of Sciences, Warsaw, Poland

**Keywords:** brain plasticity, visual and tactile Braille reading, longitudinal design, fMRI, cross-modal plasticity

## Abstract

Learning tactile Braille reading leverages cross-modal plasticity, emphasizing the brain’s ability to reallocate functions across sensory domains. This neuroplasticity engages motor and somatosensory areas and reaches language and cognitive centers like the visual word form area (VWFA), even in sighted subjects following training. No study has employed a complex reading task to monitor neural activity during the first weeks of Braille training. Since neuroplasticity can occur within days, understanding neural reorganization during early learning stages is critical. Moreover, such activation was not tested in visual and tactile domains using comparable tasks. Furthermore, implicit reading has not been studied in tactile Braille. Although visual reading in the native script occurs automatically, it remains uncertain whether the same applies to tactile reading. An implicit reading task could extend the knowledge of linguistic processing in Braille. Our study involved 17 sighted adults who learned Braille for 7 months and 19 controls. The experimental group participated in 7 testing sessions (1 week before the course, on the first day, after 1 and 6 weeks, after 3 and 7 months, and after 3 month-long hiatus). Using the fMRI Lexical Decision Task, we observed increased activity within the reading network, including the inferior frontal and supramarginal gyri, 1 week into learning in tactile and visual Braille. Interestingly, VWFA activation was observed after 1 week in the visual domain but only after 6 weeks in the tactile domain. This suggests that skill level in tactile reading influences the onset of involvement of VWFA. Once this activation was achieved, the peak level of VWFA engagement remained stable, even after the follow-up. Furthermore, an implicit reading task revealed increased activity within the reading network, including the VWFA, among participants learning Braille compared to the passive controls. Possibly, implicit reading occurs during non-reading tactile tasks where the Braille alphabet is present. We showed that the VWFA activity peak occurs faster in the visual domain compared to the tactile domain. We also showed that sighted subjects can process tactile Braille implicitly. These results enrich our understanding of neural adaptation mechanisms and the interplay between sensory modalities during complex, cross-modal learning.

## 1 Introduction

Over the past two decades, research has demonstrated that various forms of training can lead to neuroplasticity in the adult brain, encompassing physical activities, motor skills development, and language acquisition (Draganski et al., 2004; Raichlen et al., 2016; West et al., 2018; Kuper et al., 2021). Braille reading offers a unique opportunity to study cross-modal plasticity, a remarkable adaptive feature of the brain where the loss or alteration of one sensory modality induces cortical reorganization, enhancing sensory performance in the remaining modalities. Cross-modal plasticity occurs when brain structures previously devoted to processing a particular sensory input begin to accept input from a different sensory modality (Grafman, 2000). Previous research highlighted how sensory deprivation prompts extensive cortical reorganization in blind individuals. Cross-modal plasticity can be observed in the visual cortex following auditory stimulation (Ortiz-Terán et al., 2016) and tactile stimulation (Burton et al., 2004), including both early sensory regions (Sadato et al., 1996) and higher-order visual areas (Büchel et al., 1998), as well as the reading network (Reich et al., 2011). Furthermore, such plasticity can persist even after restoring the lost sense, highlighting its role in maintaining neural activity (Mowad et al., 2020). Braille training engages basic motor and sensory regions and brain areas associated with higher-order cognitive functions, providing a comprehensive model to study cross-modal plasticity (Matuszewski et al., 2021). Most importantly, however, Braille training induces changes in the activity of the reading network (Dehaene, 2010).

**Figure 1:**
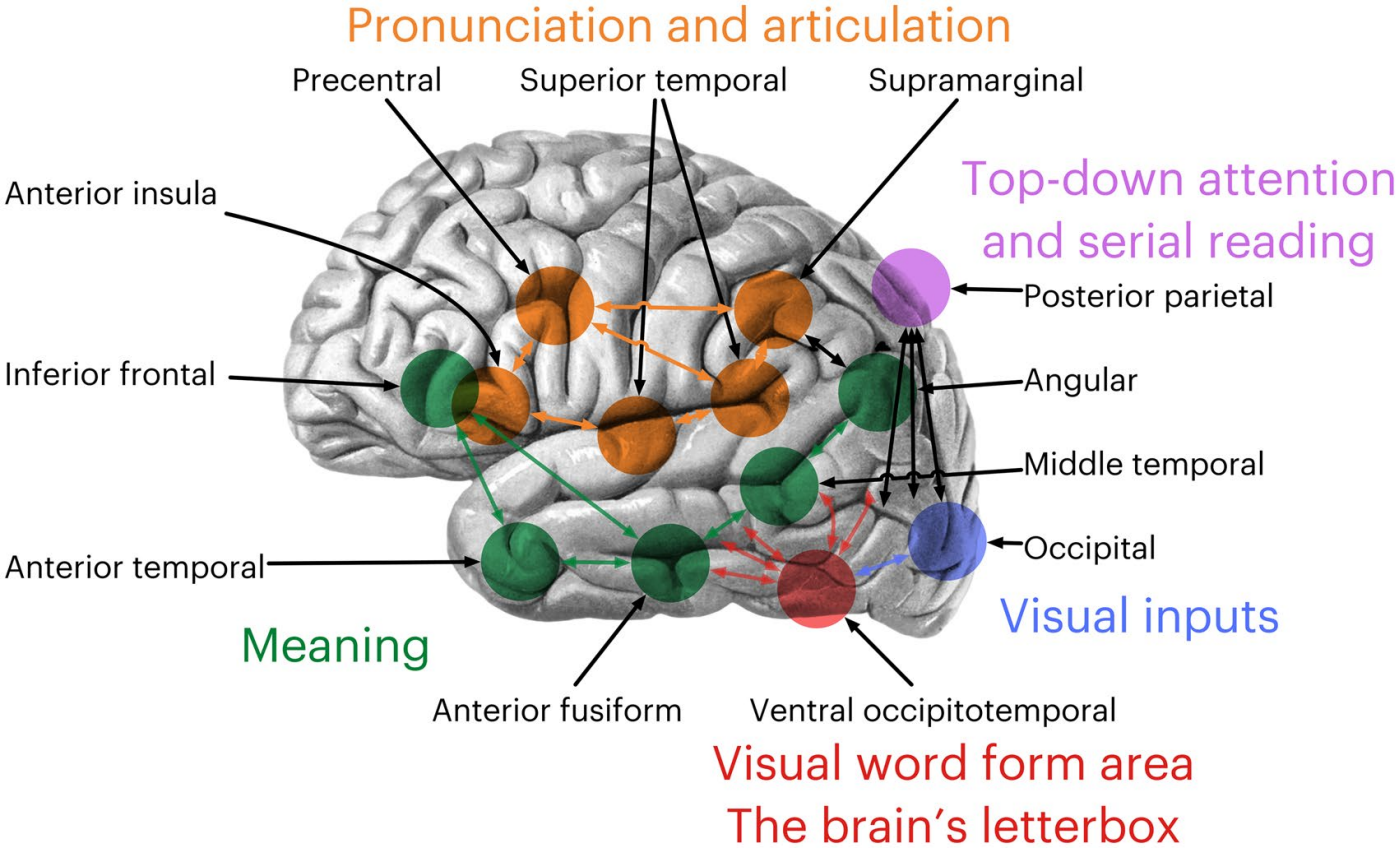
The reading network defined by Dehaene (2010). Brain adapted from an illustration from Sobotta’s (1911) Textbook and Atlas of Human Anatomy.

The reading network is a complex system of interconnected brain regions that work together to process language. It begins in the posterior parietal region, responsible for top-down attention, and the occipital areas, responsible for processing visual inputs. The ventral occipitotemporal cortex—the visual word form area (VWFA)—comes next, acting as the brain’s letterbox (Dzięgiel-Fivet et al., 2021). This area is crucial for recognizing and interpreting letters and words. Previous research has identified this area as involved in sighted adults reading visually (Lerma-Usabiaga et al., 2018) and blind individuals reading Braille tactilely (Reich et al., 2011). The inferior frontal gyrus, anterior temporal gyrus, anterior fusiform gyrus, middle temporal gyrus, and angular gyrus are responsible for retrieving meaning in language, allowing us to understand and interpret written words. The superior temporal gyrus, anterior insula, precentral gyrus, and supramarginal gyrus are responsible for pronunciation and articulation. These areas form the reading network, working together to process and understand written language (Figure 1).

Importantly, cross-modal plasticity is not limited to individuals with sensory impairments. For instance, when blindfolded, sighted adults also show increased early visual cortex activation during tactile stimuli discrimination (Merabet et al., 2008). More recent studies using both complex tasks, such as the Lexical Decision Task (LDT) and simple word reading tasks, have provided valuable insights into the dynamics of training-induced plasticity, including, but not limited to, cross-modal plasticity, through Braille reading in sighted individuals (Siuda-Krzywicka et al., 2016; Matuszewski et al., 2021). These studies collectively indicate that tactile reading stimulates motor and sensory regions, the reading network, and brain areas linked with advanced cognitive functions, such as the VWFA. Notably, the first effects were observed after 3 months of training. However, given that neuroplasticity can manifest within weeks (Debowska et al., 2016), days (Chen et al., 2019), or even minutes (Sagi et al., 2012), the temporal dynamics of neural reorganization during Braille’s early learning stages should also be examined. This can be achieved by using a longitudinal approach. By assessing brain activity at multiple time points, we can capture the progression and consolidation of learning-induced plasticity. Such an approach provides a deeper understanding of the learning process than the pre-post approach used in many longitudinal studies (Mårtensson et al., 2012; Grant et al., 2015; Legault et al., 2019) and allows distinguishing between initial rapid changes and longer-term adaptations in the brain. Such detailed temporal mapping is crucial for understanding how the brain reorganizes itself to accommodate new skills, particularly in the context of cross-modal plasticity.

No study has leveraged a linguistic task to observe neural activation shifts within the first few weeks of Braille learning. While increased brain activity has been observed in the primary somatosensory cortex and the fusiform gyrus (Debowska et al., 2016), it was done in a task with a strong detection and recognition component present, where no language-related decision-making was required. Since reading acquisition requires a distinctive manner of linguistic processing in response to stimuli, it is crucial to distinguish it from other processes, such as detecting simple patterns or objects, which may still induce activity in the reading network (Reed et al., 2004). Using linguistic tasks makes it possible to assert that the reorganization reflects increased abilities to identify and process Braille symbols and the appropriation of linguistic meaning to abstract symbol representations that constitute the tactile script. Moreover, a reading task and the presence of a linguistic component allows us to precisely measure the learning progress through quantifiable metrics such as the number of letters or words or correctly classified Braille stimuli as words or pseudowords. Significant changes in the performance and activation within the reading network would allow us to draw valuable comparisons between the neural regions activated during Braille reading and those involved in reading through sight. These comparisons are vital for understanding how different sensory inputs influence the reading network and cognitive development.

In sighted people, the Braille alphabet can be learned using both vision and touch. Contrasting visual and tactile Braille-related activity could help identify which regions of the reading network are specifically engaged in tactile reading. Previous research involving the same group of sighted Braille teachers has demonstrated that visual Braille reading, which lacks natural line junctions, is significantly less efficient than reading scripts like Cyrillic. Even with previous visual Braille knowledge, reading was slower and more prone to errors than in the Cyrillic alphabet learning group after just 3 months of learning (Bola et al., 2017a). Although tactile and visual reading have been tested in an fMRI setting before (Siuda-Krzywicka et al., 2016), no direct comparison has been computed. This highlights the need for a direct comparison to understand the specific neural mechanisms engaged in tactile Braille reading, preferably in naive people with no prior Braille knowledge.

Previous research with sighted Braille learners has focused mainly on explicit reading or simple tactile pattern recognition tasks. While invaluable when it comes to understanding cross-modal plasticity, these tasks do not consider that in everyday life, much of the interaction of sighted individuals with reading is done without conscious effort, for example, when reading a book or reading subtitles while watching a movie. This form of reading can be defined as implicit. Studies have demonstrated that implicit reading can induce significant neural activity similar to that observed in explicit reading tasks. For instance, even when subjects are instructed to perform a nonlinguistic visual feature detection task, the presence of words or pseudowords activates a widespread neuronal network that aligns with areas of the reading network (Thuy et al., 2004; Price and Devlin, 2011). This suggests that the brain processes words beyond the functional demands of the task, highlighting the underlying neural mechanisms that support implicit reading. However, to our knowledge, no one has tested whether implicit reading in the tactile domain can induce a different functional response in the brain. With the acquisition of linguistic context for the Braille symbols, such implicit reading could also emerge even in a non-linguistic task. Thus, the activation of regions in the reading network in response to tactile Braille stimulation without an overt linguistic task would further support the multimodal and modality-independent nature of the brain areas involved in reading.

Finally, learning might evoke potential transfer effects, although the literature on this topic remains inconclusive (Soveri et al., 2017). Cognitive training paradigms, including linguistic ones, are proposed as tools in neurorehabilitation or as preventive measures to delay the onset of age-related cognitive decline. In this context, it is a valid question whether acquiring a new skill, such as reading in a tactile domain, translates into improved performance on an unrelated cognitive task. We incorporated n-back and Stroop tasks in our study design to test this. These tasks are well-established measures of working memory and cognitive control, respectively, and they allowed us to investigate potential generalization cognitive effects induced by complex learning.

Current research consisted of a seven-month tactile Braille reading course combined with functional neuroimaging, including Braille processing in visual and tactile modalities as well as behavioral testing across multiple time points ranging from days to months after training onset and aimed to answer the following questions:

(1) “At which stage does the reading network become involved in visual and tactile Braille reading?” We postulated that the reading network would be engaged during visual reading within the first week of learning. However, given the inherent complexity of tactile reading, we expected the VWFA and inferior frontal gyrus (IFG) involvement to become apparent in the tactile domain after a more extended period than visual reading.
(2) “What are the brain networks engaged in tactile and visual Braille reading, and which regions are involved in Braille word processing regardless of the presentation domain?” Studies on blind people showed that VWFA constitutes a reading center independent of visual experience (Reich et al., 2011). We hypothesized, therefore, that areas of the reading network, such as the IFG and VWFA, would be involved in both visual and tactile reading tasks.
(3) “Can the involvement of the reading network be observed during implicit Braille reading?” We hypothesized that a comparison between Braille learners and passive controls would reveal a higher activity level in the reading network areas among the learning group during an implicit reading task (Thuy et al., 2004; Brem et al., 2009).

## 2 Materials and methods

### 2.1 Participants

Twenty-one right-handed female university students were recruited to the experimental group in the study. One participant resigned due to health-related problems before the learning started. Two participants took part in the Braille course but quit before it ended. One participant did not participate in the follow-up experimental session. Therefore, the final number of participants was 17 (Age M = 21.00; SD = 1.37). We collected written informed consent before the study. We recruited all participants upon completing an online questionnaire focused on demographics, education, general linguistics, and health-related issues. To increase the subjects’ motivation to complete the training course, we recruited only students from a pedagogical university (The Maria Grzegorzewska University) in the experimental group (Siuda-Krzywicka et al., 2016; Bola et al., 2017b; Matuszewski et al., 2021). This is one of the very few schools with a possibility of a degree in typhlopedagogy - a branch of special education dealing with the education of visually impaired individuals.

Additionally, 21 right-handed demographically matched female students were recruited as a passive control group. Two participants resigned from the study before the end of its main part. The final sample consisted, therefore, of 19 participants (Age M = 20.84; SD = 1.57). Our recruitment criteria for controls were the same as in the experimental group, though we did not restrict our recruitment process to the pedagogical school. Furthermore, we matched controls demographically to the experimental group and found no statistically significant differences in age (*t* (34.77) = −0.33; *p* = 0.75; *d* = −0.11) and number of known foreign languages (*t* (30.90) = −0.59; *p* = 0.56; *d* = −0.19).

### 2.2 Experimental design

**Figure 2:**
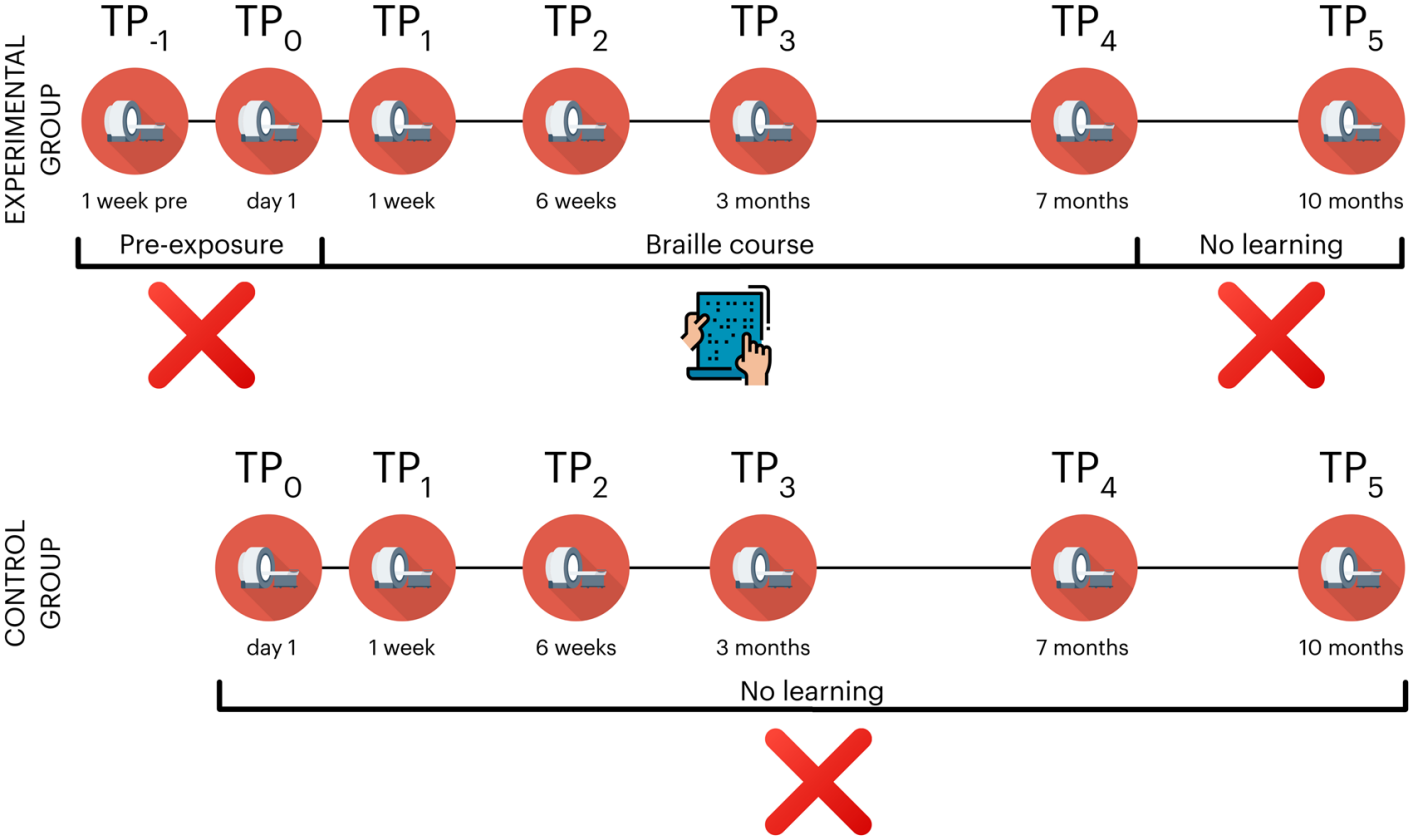
Overview of experimental design in the Braille learning (experimental) and passive control groups. The control group did not participate in TP_−1_ (a prescan a week before the beginning of the course); TP, time point. Icons created by Flaticon.

During the study, the subjects in the experimental group took part in 7 behavioral and neuroimaging sessions: 2 pre-exposure (time point −1; TP_−1_ and TP_0_), 3 during (TP_1_ - TP_3_), 1 at the end (TP_4_) and one 3 months after the end of training (TP_5_). The control participants did not participate in the 1^st^ pre-exposure session (TP_−1_) (see Figure 2 and Table 1 for an overview). The Committee for Research Ethics of the Institute of Psychology of the Jagiellonian University approved this study.

**Table 1:** Overview of the behavioral and fMRI tasks in the Braille learning (experimental) and passive control groups.

Group	Time point	Task										Time interval
		fMRI + behavioral				Behavioral						
		LDT			DD6	Letter reading		Word reading		Stroop	N-back	
		Tactile Braille	Visual Braille	Visual Print	Tactile	Tactile	Visual	Tactile	Visual	Visual	Visual	
Experimental	TP_−1_	✓	✓	✓	✓	✓	✓	✓	✓			1 week before training
	TP_0_	✓	✓	✓	✓	✓	✓	✓	✓	✓	✓	Training onset
	TP_1_	✓	✓	✓	✓	✓	✓	✓	✓			1 week
	TP_2_	✓	✓	✓	✓	✓	✓	✓	✓			6 weeks
	TP_3_	✓	✓	✓	✓	✓	✓	✓	✓			3 months
	TP_4_	✓	✓	✓	✓	✓	✓	✓	✓	✓	✓	7 months (end of training)
	TP_5_	✓	✓	✓	✓	✓	✓	✓	✓	✓	✓	10 months
Control	TP_0_				✓					✓	✓	Study onset
	TP_1_				✓							1 week
	TP_2_				✓							6 weeks
	TP_3_				✓							3 months
	TP_4_				✓					✓	✓	7 months
	TP_5_				✓					✓	✓	10 months

LDT, Lexical Decision Task; DD6, 6-dots Detection Task. Letter reading and word reading behavioral tasks were done using the Braille alphabet.

### 2.3 Braille course

The tactile Braille reading course was scheduled for 6 months. Due to the ongoing SARS-CoV-2 pandemic and no possibility of conducting on-site research at the scheduled end of the course, we extended its duration to 7 months. The course involved 9 online meetings with an instructor and asynchronous work of all the participants (see Figure 2 for an overview) with 6 sets of Braille study cards (60 cards in the set for the first month and 30 in each set dedicated for the months 2–6). Each study card’s workload was estimated to take from 10 to 20 min of tactile reading - a total of approximately 52.5 h of self-practicing without any repetition. The first 3 meetings on the first, seventh, and fourteenth days were held in small groups of at most 5 students. All the other meetings were held in larger groups of around 10 students. To ensure similar progress in learning crucial for the initial stage of training in the first week, the participants received a list of individual tasks to do on a specific day of the course. The remaining weeks of the first month were organized in a weekly manner. The instructions for the remaining months were not organized in any timely manner - the participants were free to learn and process the material at their own pace, though they were highly encouraged to do one card a day. The course focused primarily on teaching to read Braille in the tactile domain. However, because the participants are sighted, they naturally supported the learning process using vision.

**Figure 3:**
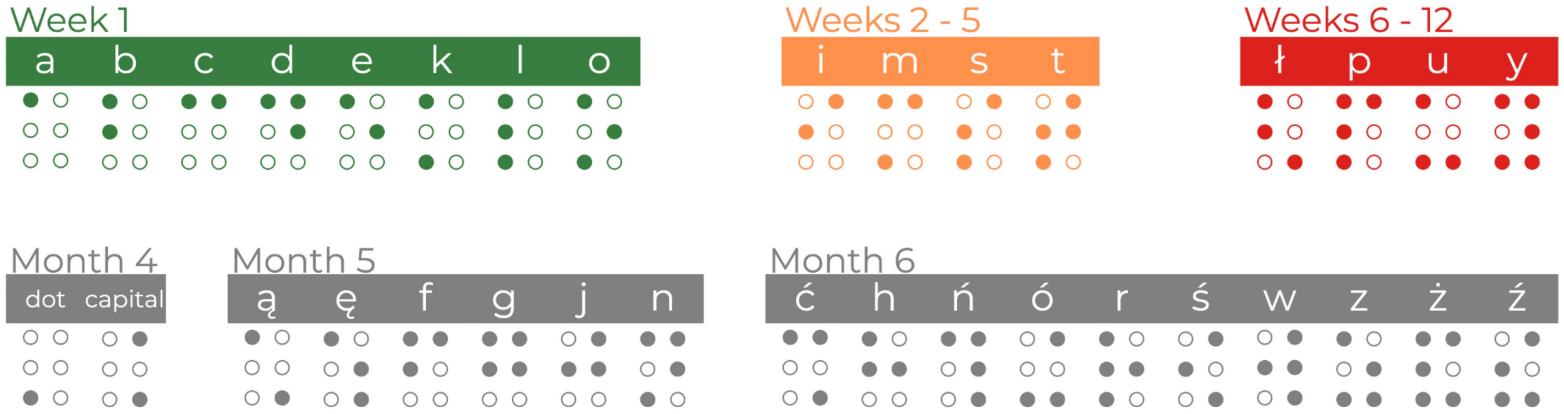
The overview of learning material during the Braille course. The core letters used to create stimuli for fMRI tasks are colored green, orange, and red. Additional letters and symbols (marked in gray) were introduced during the course to equalize subjects’ engagement.

In line with previous Braille courses (Bola et al., 2016; Matuszewski et al., 2021), the current course introduced 32 letters and 2 symbols. The first week of the course was the most demanding. The participants had to practice the proper way of moving the hand through the Braille card or shape discrimination during the meetings and later by themselves. They were instructed to read with their right index fingers and navigate the Braille card using their left index finger. At the same time, the instructor introduced the easiest 8 letters (A, B, C, D, E, K, L, O). During weeks 2–5, 4 new letters (I, M, S, T) were introduced. During weeks 6–12, the participants were presented with the next 4 letters (Ł, P, U, Y). Sixteen letters introduced in the first 12 weeks of the course were considered the core letters of the study and were used in stimuli during the fMRI experimental sessions. While months 4–6 of the course focused on mastering the core letters, they also introduced new material to maintain high interest and engagement. The fourth month of the course introduced 2 new signs - a Braille dot and the capital letter sign. The fifth month of the course introduced 6 additional letters of the Polish alphabet (Ą, Ę, F, G, J, N). The sixth month of the course introduced the remaining 10 letters (Ć, H, Ń, Ó, R, Ś, W, Z, Ż, Ź). See Figure 3 for a graphical overview of the material during the Braille course.

### 2.4 Behavioral measures

#### 2.4.1 Braille reading tests

At each TP, we assessed the experimental group’s Braille words and letters reading skills in both visual and tactile domains. For both tasks, participants read aloud as many words or letters as possible in 1 min, and we counted correctly read stimuli. For the word-reading test, tests consisted of 15 words that were 3 to 5 letters long. Crucially, in every experimental session after the course started, the words selected for the test contained only those core letters that that specific stage of the Braille course had already introduced. The only exceptions to the rule were TP_−1_ and TP_0_, the time points before the beginning of the course, which used the whole set of core letters presented in the core part of the course.

On the other hand, the letter-reading test comprised 28 letters randomly selected from the learning material. To maintain consistency with the course, we used a pseudo-randomized order, ensuring that the already covered letters were presented before the ones that had not been introduced.

#### 2.4.2 Cognitive tests

On 3 separate occasions - TP_0_ (day 1), TP_4_ (7 months), and TP_5_ (follow-up) the participants from both the experimental and the control group did 2 cognitive tasks: the Stroop task and the N-back task. All experiments were programmed using the Presentation software (Neurobehavioral Systems, Inc., 2022). We used versions of the tasks available on the software’s website as part of the Neurobehavioral Systems’ experiment packages.^1^ In both tasks, a Cedrus RB-540 Response Pad was used to gather responses.

The Stroop task consisted of two separate blocks. In the ink blocks, participants were asked to identify the color of stimuli. In the color name blocks, their task was to determine the name of the color spelled by the word. Each experimental stimulus could appear in or be named with one of four target colors (red, green, blue, or yellow). While the control stimuli in the ink blocks contained Xs rather than color names, the control stimuli in the color name blocks were written in black. The experiment consisted of 3 ink and 3 color name blocks, which were presented alternately. A single block comprised 72 trials, half incongruent and half neutral. A single trial started with a fixation cross presented for 500 ms, followed by the stimulus presented until the answer was given (but no longer than 5,000 ms). An empty interval of 1,000 ms separated the trials. Before the main experiment, the participants did a test run of the task, which consisted of 12 trials of each block type. Feedback was given only in the test run. The task lasted around 5 min, with the fastest participant finishing in 4 min and 51 s and the slowest in 6 min and 40 s.

The visual n-back task was created using single letters on three levels of difficulty: 1-back, 2-back, and 3-back. The participants were presented with a sequence of single letters and had to respond with a button press every time the presented letter was the same as the one n steps back. A single block comprised 50 trials, 10 of which were the target and 40 were control trials. A single trial consisted of a stimulus presented for 500 ms. An empty interval of 1,000 ms separated the trials. Before the main experiment, the participants did a test run of the task, which consisted of 8 trials of each block type.

### 2.5 MRI protocols

We acquired the MRI data using a Siemens Trio 3 T scanner with a 32-channel coil. Structural T1-weighted (T1w) image was acquired with a standard MPRAGE sequence with the following parameters: Field of view: 256 × 256 mm, voxel size: 1 × 1 × 1 mm, Repetition time: 2,530 ms, Echo time: 3.32 ms, Flip angle: 7°, 176 slices. The functional and resting state data were acquired using an Echo Planar Imaging (EPI) pulse sequence (FOV: 210 × 205 mm, voxel size: 2.5 × 2.5 × 2.5 mm, Repetition time: 1410 ms, Echo time: 30.4 ms, Flip angle: 56°, Multiband factor: 3).

### 2.6 fMRI tasks and stimuli

#### 2.6.1 Lexical decision tasks

**Figure 4:**
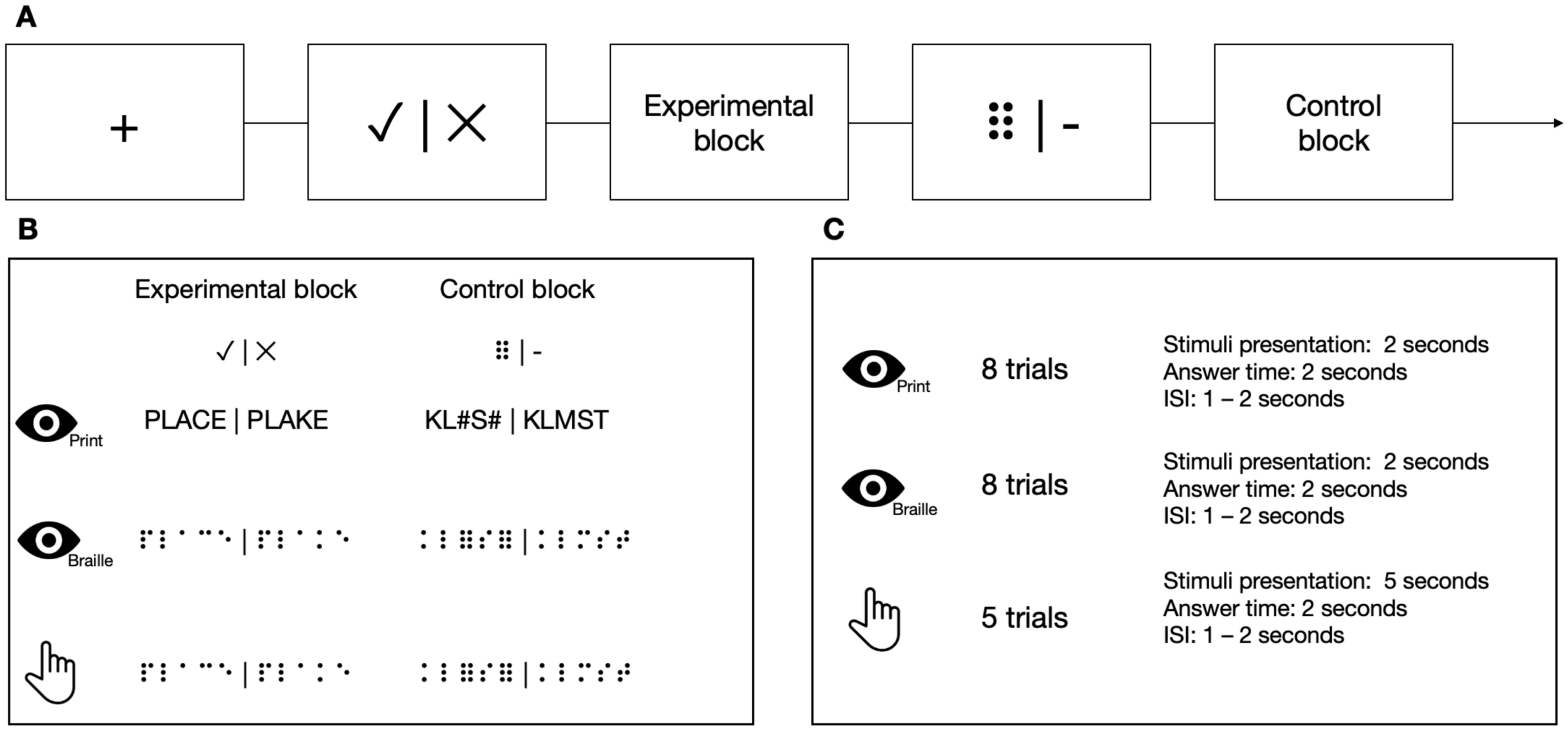
**(A)** The stimuli in Lexical Decision Tasks were presented in a block design. **(B)** In the experimental blocks, the participants had to decide whether the stimulus was a real word or a pseudoword. In the control blocks, the participants had to determine whether the presented stimulus contained two six-points (or hash symbols in the print visual task). **(C)** In visual tasks, each block consisted of eight stimuli. In the tactile task, each block consisted of five stimuli. Icons created by Flaticon.

At each TP, participants from the experimental group performed 3 types of LDTs in the MRI scanner in the following order: visual using the Polish print alphabet, tactile using the Braille alphabet, and visual using the Braille alphabet (Figure 4). Tactile stimuli were presented via an MRI-compatible Braille display (Debowska et al., 2013), and visual stimuli were presented on a screen. In the experimental condition, the participants had to decide whether the presented sequence of letters constituted a real word or was just a set of letters that resembled one but does not exist in the Polish language (pseudoword). In the control condition, the task was to decide whether a presented sequence of characters contained exactly two nonlinguistic symbols (hash signs (#) in the Latin alphabet task or Braille six-dot characters (⠿) in the Braille) among random consonants. The stimuli were prepared so that half of the trials contained exactly two nonlinguistic symbols, and half contained none of them. The participant responded (nonlinguistic symbols present/not present) using a response pad with their left hand at the end of each trial. In visual tasks, each block consisted of 8 stimuli presented for 1 s each. In the tactile task, each block consisted of 5 stimuli presented for 5 s each. Each block was preceded by a fixation cross (in pseudo-randomized duration between 6 and 8 s) and a visual indication of the block to be presented (2 s). Every stimulus was then succeeded by response time (2 s) and an inter-stimulus interval (ISI, 1–2 s).

In Braille tasks, the words were 3–5 letters long, consisting only of the core letters from the course (see point 2.3 for details). The only exceptions to these rules were TP_−1_ and TP_0_, the time points before the beginning of the course, which used the whole set of core letters presented in the core part of the course. All words had a frequency of occurrence higher than 1 per million, according to the SUBTLEX-PL database (Mandera et al., 2015). The pseudowords were created by changing 1 letter in the word.

#### 2.6.2 6-dots detection task (DD6)

**Figure 5:**
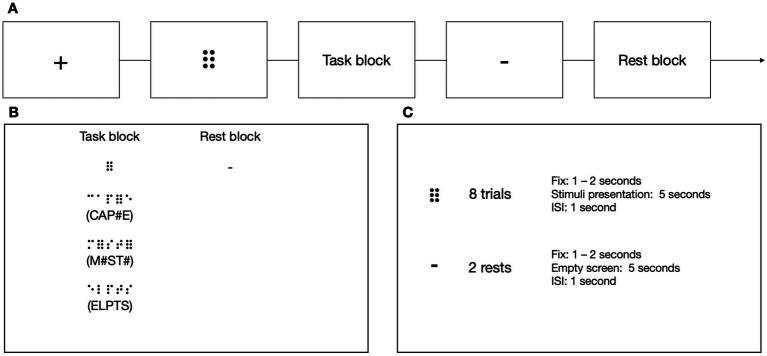
**(A)** The stimuli in the 6-dots Detection Task were presented in a block design. **(B)** In the task blocks, the participants had to move their right index finger from left to right and press a button with their left hand every time they detected a 6-dot symbol (⠿). The participants were unaware that some of the presented stimuli contained a simple three-letter long word. The participants had to wait during the rest while no sequence was presented. **(C)** Every experimental block consisted of eight stimuli, and every rest block consisted of two resting periods. Icons created by Flaticon.

To test subjects’ ability to read implicitly, we introduced a new task that could be used in both the experimental and control groups, the latter unfamiliar with the Braille alphabet (Figure 5). In the experimental condition, the participants sequentially moved their right index finger from left to right. They pressed a response button with their left hand whenever they detected a symbol of 6 dots (⠿) among random letter strings, resulting in 0, 1, or 2 responses per trial. The participants were not informed that approximately half of the stimuli in the experimental block included short, three-letter long words. The experimental blocks were separated by rest. Each block was preceded by a fixation cross (in pseudo-randomized duration between 6 and 8 s) and a visual indication of the block to be presented (1 to 2 s). In the experimental condition, each block consisted of 8 stimuli presented for 5 s each and an ISI which lasted 1 s. Each rest lasted 12 s.

### 2.7 Statistical analysis of behavioral data

We used a repeated measures analysis of variance (rmANOVA) to analyze the Braille behavioral data. We computed a one-way analysis with time as a factor for Braille reading tasks. For the LDTs, we ran a series of two-way rmANOVAs (for each task separately), with time and condition as factors. In DD6, we ran a two-way mixed ANOVA, with time as a within-subject factor and group as a between-subject factor. We computed a three-way mixed ANOVA for cognitive tasks, with time and condition as within-subject factors and the group as the between-subject factor.

All *post hoc* tests were computed with Bonferroni correction for multiple comparisons. Greenhouse–Geisser F-tests and degrees of freedom corrections were used for cases with a violated sphericity assumption. Analyses were performed using the pingouin (Vallat, 2018) and statsmodels (Seabold and Perktold, 2010) packages written in Python and, in the case of a three-way ANOVA, using the rstatix library in R (Kassambara, 2023).

### 2.8 MRI data preprocessing

Each subject’s data underwent preprocessing with fMRIPrep (Esteban et al., 2022). T1-weighted (T1w) images were corrected for intensity non-uniformity (INU) with N4BiasFieldCorrection (Tustison et al., 2010), distributed with ANTs 2.3.3 (Avants et al., 2008, RRID:SCR_004757). The T1w-reference was then skull-stripped with a Nipype implementation of the antsBrainExtraction.sh workflow (from ANTs), using OASIS30ANTs as the target template. Brain tissue segmentation of cerebrospinal fluid (CSF), white matter (WM) and gray matter (GM) was performed on the brain-extracted T1w using FAST [FSL 6.0.5.1:57b01774, RRID:SCR 002823, (Zhang et al., 2001)]. A T1w-reference map was computed after registration of T1w images from all sessions (after INU-correction) using mri_robust_template [FreeSurfer 6.0.1, (Reuter et al., 2010)]. Volume-based spatial normalization to two standard spaces (MNI152NLin2009cAsym, MNI152NLin6Asym) was performed through nonlinear registration with antsRegistration (ANTs 2.3.3), using brain-extracted versions of both T1w reference and the T1w template. The following templates were selected for spatial normalization: ICBM 152 Nonlinear Asymmetrical template version 2009c [(Fonov et al., 2009], RRID:SCR_008796; TemplateFlow ID: MNI152NLin2009cAsym), FSL’s MNI ICBM 152 non-linear 6th Generation Asymmetric Average Brain Stereotaxic Registration Model [(Evans et al., 2012), RRID:SCR_002823; TemplateFlow ID: MNI152NLin6Asym].

Each functional session for every subject underwent preprocessing with fMRIPrep (Esteban et al., 2022). A reference volume was created by aligning and averaging the single-band references (SBRefs). Preprocessing included head-motion parameters estimation using mcflirt [FSL 6.0.5.1:57b01774, (Jenkinson et al., 2002)], and slice-time correction using 3dTshift from AFNI [(Cox and Hyde, 1997), RRID: SCR_005927]. The BOLD time series were resampled onto their original, native space, correcting for head motion, producing preprocessed BOLD in the original space. Co-registration to the T1w reference used mri_coreg (FreeSurfer) and flirt [FSL 6.0.5.1:57b01774, (Jenkinson and Smith, 2001)]. We calculated several confounding time series, including framewise displacement (FD), DVARS, and three region-wise global signals using Nipype (Power et al., 2014). Noise correction was applied with physiological regressor extraction [CompCor, (Behzadi et al., 2007)]. After high-pass filtering the preprocessed BOLD time series, two CompCor variants were used: temporal (tCompCor) and anatomical (aCompCor). Motion artifact removal was done using independent component analysis [ICA-AROMA, (Pruim et al., 2015)] on the preprocessed BOLD on MNI space time-series post spatial smoothing. The functional data was smoothed with a 6 mm FWHM Gaussian kernel. Noise regressors were placed in the corresponding confounds file. Resamplings were done using antsApplyTransforms (ANTs) and mri_vol2surf (FreeSurfer). Internal operations used Nilearn 0.8.1 [(Abraham et al., 2014), RRID:SCR_001362].

### 2.9 Statistical analysis of functional data

First, functional data of three Lexical Decision Tasks (Print, Visual Braille, and Tactile Braille) and the 6-dots detection task were analyzed using a general linear model (GLM) at the subject level. The timings of experimental and control blocks (rest in DD6 task) were entered for each time point separately, together with six head movement regressors. The hemodynamic response was modeled using the default canonical functions of SPM 12.7771 (Penny et al., 2011). All data were filtered with a 128 Hz high-pass filter. In the LDTs, the experimental > control contrast was computed separately at each time point for every variant of the task (print, visual Braille, tactile Braille) for each subject. In the DD6, the experimental contrast was computed separately for task activation against the global baseline at each time point for each subject. Each contrast was additionally masked using a group-level brain mask from the fMRIPrep preprocessing pipeline.

On the group level, the GLMs were specified using SPM12’s flexible factorial models tailored separately to find answers to each research question. Results were thresholded at a voxel level with a Family-Wise Error (FWE) comparisons correction with a *p*-value of 0.05 and a cluster extent of 20 voxels. All anatomical structures were labelled with the Automated Anatomical Labelling (AAL) atlas (Rolls et al., 2020).

To find answers to the research questions introduced in this paper, a series of analyses has been carried out on several models:

To answer the first question (at which stage does the reading network become involved in visual and tactile Braille reading?), we introduced 2 main rmANOVA models for each Braille Lexical task (Visual and Tactile), with time (TP_0_ - TP_5_) and subject as factors. To control the effect of task repetition, we introduced 3 additional models: paired t-tests for each Braille task with pre-training time points (TP_−1_ and TP_0_) and subject as factors, as well as a rmANOVA model for the Print LDT, with time (TP_0_ - TP_5_) and subject as factors. Additionally, we extracted the contrast estimates for every time point from the single most active voxel in the 4 most active areas of the reading network in Tactile and Visual Braille LDTs (using experimental condition > control condition contrast). This allowed us to visually inspect the time courses throughout the study and compare the general trends in activation levels.

We aimed to answer our second question (what are the specific brain networks engaged in Tactile and Visual Braille reading?) by creating a single rmANOVA model with the Lexical task (Print, Visual Braille, and Tactile Braille [TP_0_–TP_5_ pooled together]) and subject as factors. To find regions active in Braille reading regardless of domain, we computed the conjunction of the main effects of the condition in Tactile and Visual Braille LDT. We extracted voxels not specific to Braille reading voxels by excluding any active ones in the Print LDT. To find Braille-activated regions specific to tactile reading, we looked at the main effect of condition in this Tactile LDT with voxels active in Visual Braille or Print LDT excluded. To find Braille-activated regions specific to visual reading, we looked at the main effect of condition in this LDT with voxels active in either Tactile Braille or Print LDT excluded.

A final model was introduced to answer the third question (can the involvement of the reading network be observed during implicit Braille reading?) by doing mixed ANOVA and analyzing the DD6 task with group (experimental, control) and time (TP_0_-TP_5_) interaction and subject as factors.

## 3 Behavioral results

### 3.1 Braille reading tests

**Figure 6:**
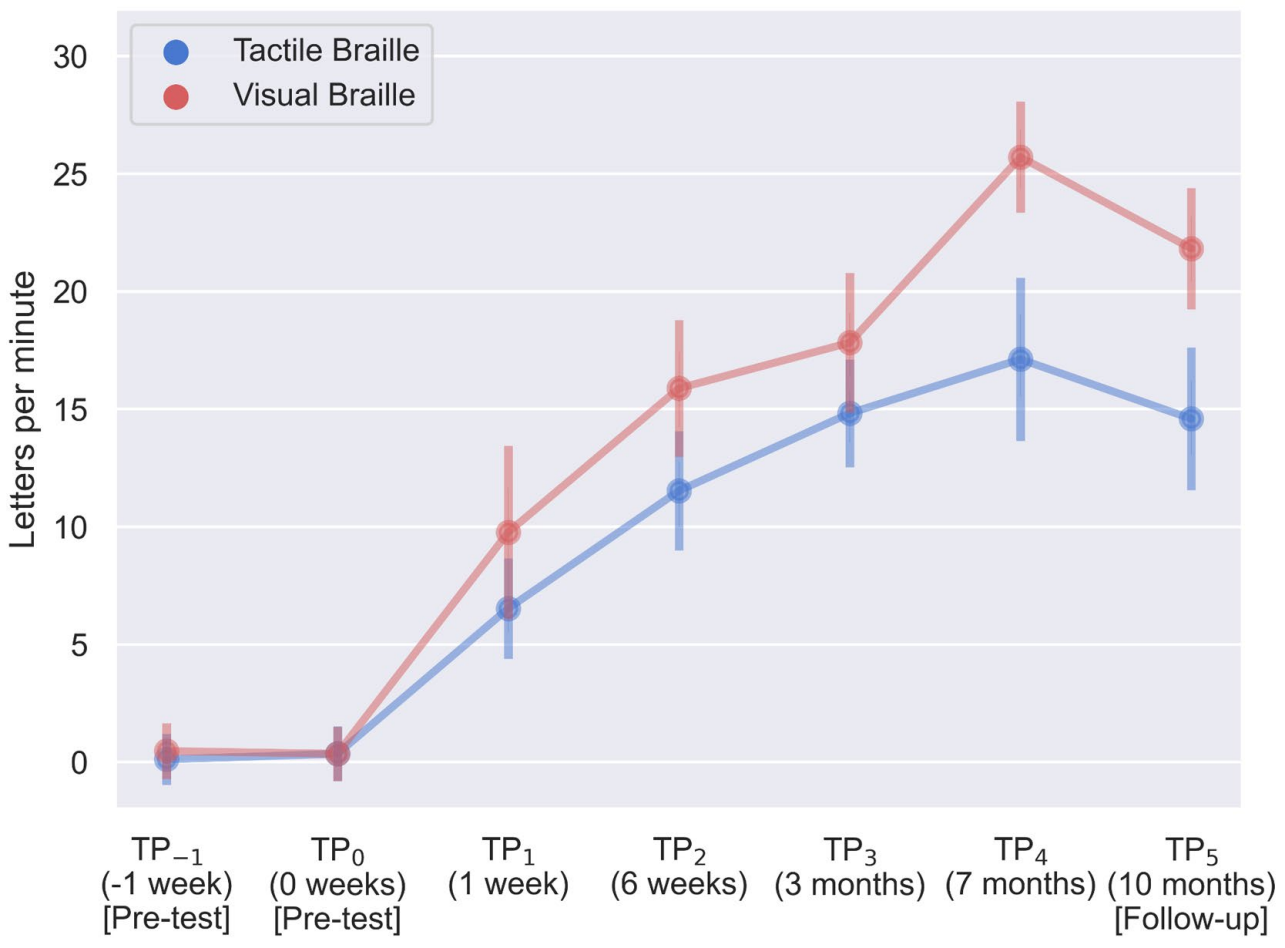
Visual and tactile Braille letters reading speed during training. Error bars represent standard deviations adjusted for within-subject designs (Cousineau, 2005); TP, time point.

During our assessment of Braille reading proficiency, participants were evaluated across both visual and tactile domains at different time intervals. In the visual Braille letter reading assessment, a one-way rmANOVA revealed a significant main effect of time, *F* (6, 96) = 263.43; *p* < 0.001; *eta_p_^2^* = 0.94. All post-hoc comparisons were significant except for TP_−1_ and TP_0_, TP_2_ and TP_3_, and TP_3_ and TP_5_. Notably, performance improved consistently from TP_1_ to TP_4_ but declined at TP_5_ after a 3-month break (Table 2; Figure 6).

**Table 2:** Visual Braille letters reading speed: post-hoc comparisons between time points.

Time point	*M*	*SD*	TP_−1_	TP_0_	TP_1_	TP_2_	TP_3_	TP_4_
TP_−1_	0.47	0.87						
TP_0_	0.35	0.61	1					
TP_1_	9.76	4.10	< 0.001	< 0.001				
TP_2_	15.88	3.37	< 0.001	< 0.001	< 0.001			
TP_3_	17.82	2.98	< 0.001	< 0.001	< 0.001	1		
TP_4_	25.71	2.71	< 0.001	< 0.001	< 0.001	< 0.001	< 0.001	
TP_5_	21.82	2.94	< 0.001	< 0.001	< 0.001	0.001	0.053	< 0.001

TP_−1_, Pre-test (1 week before the course); TP_0_, Pre-test (On the day of the course); TP_1_, 2 week into the course; TP_2_, 6 weeks into; TP_3_, 3 months into; TP_4_, 7 months into; TP_5_, Follow-up (3 months after the end of the course); M, Mean; SD, Standard deviation. p values were corrected with Bonferroni correction for multiple comparisons.

In the tactile Braille letter reading assessment, a main effect of time was observed, *F* (6, 96) = 153.53; *p* < 0.001; *eta_p_^2^* = 0.91. All comparisons were significant, with the exceptions of TP_−1_ and TP_0_, TP_2_ and TP_5_, TP_3_ and TP_4_, TP_3_ and TP_5_, and TP_4_ and TP_5_ (Table 3; Figure 6).

**Table 3:** Tactile Braille letters reading speed: post-hoc comparisons between time points.

Time point	*M*	*SD*	TP_−1_	TP_0_	TP_1_	TP_2_	TP_3_	TP_4_
TP_−1_	0.12	0.33						
TP_0_	0.35	0.61	1					
TP_1_	6.53	2.60	< 0.001	< 0.001				
TP_2_	11.53	2.92	< 0.001	< 0.001	< 0.001			
TP_3_	14.82	2.32	< 0.001	< 0.001	< 0.001	0.01		
TP_4_	17.12	3.95	< 0.001	< 0.001	< 0.001	< 0.001	0.54	
TP_5_	14.59	3.45	< 0.001	< 0.001	< 0.001	0.11	1	0.38

TP_−1_, Pre-test (1 week before the course); TP_0_, Pre-test (On the day of the course); TP_1_, 1 week into the course; TP_2_, 6 weeks into; TP_3_, 3 months into; TP_4_, 7 months into; TP_5_, Follow-up (3 months after the end of the course); M, Mean; SD, Standard deviation. *p* values were corrected with Bonferroni correction for multiple comparisons.

**Figure 7:**
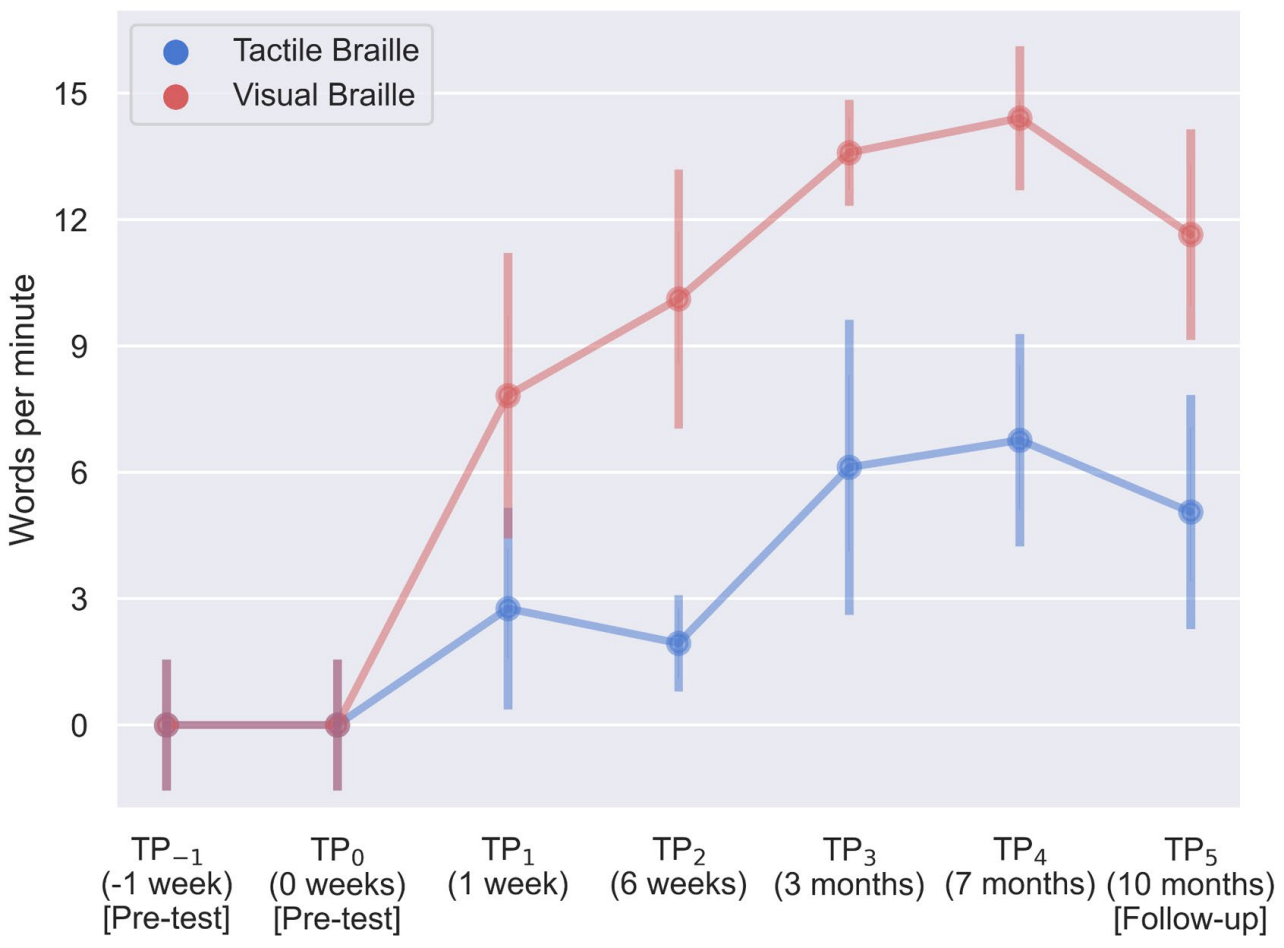
Visual and tactile Braille word reading speed during training. Error bars represent standard deviations adjusted for within-subject designs (Cousineau, 2005); TP, time point.

The visual Braille word reading analysis revealed a significant main effect of time, *F* (6, 96) = 115.43; *p* < 0.001; *eta_p_^2^* = 0.88. All comparisons were significant, except for TP_−1_ and TP_0_, TP_1_ and TP_2_, TP_2_ and TP_5_, TP_3_ and TP_4_, TP_3_ and TP_5_, and TP_4_ and TP_5_ (Table 4; Figure 7).

**Table 4:** Visual Braille words reading speed: post-hoc comparisons between time points.

Time point	*M*	*SD*	TP_−1_	TP_0_	TP_1_	TP_2_	TP_3_	TP_4_
TP_−1_	0.00	0.00						
TP_0_	0.00	0.00	1					
TP_1_	7.82	4.11	< 0.001	< 0.001				
TP_2_	10.12	3.52	< 0.001	< 0.001	1			
TP_3_	13.59	1.91	< 0.001	< 0.001	0.001	0.004		
TP_4_	14.41	0.87	< 0.001	< 0.001	< 0.001	0.001	1	
TP_5_	11.65	3.44	< 0.001	< 0.001	0.03	1	0.18	0.12

TP_−1_, Pre-test (1 week before the course); TP_0_, Pre-test (On the day of the course); TP_1_, 1 week into the course; TP_2_, 6 weeks into; TP_3_, 3 months into; TP_4_, 7 months into; TP_5_, Follow-up (3 months after the end of the course). *p* values were corrected with Bonferroni correction for multiple comparisons. No *p*-value was calculated for the TP_−1_ and TP_0_ comparison due to a lack of any variability of the results—we filled the missing value with a *p*-value of 1.

Lastly, results were significant in the tactile word reading domain with *F* (6, 96) = 23.61; *p* < 0.001; *eta_p_^2^* = 0.60. All post-hoc tests were significant, except for TP_−1_ and TP_0_, TP_1_ and TP_2_, TP_1_ and TP_3_, TP_1_ and TP_5_, TP_3_ and TP_4_, and TP_3_ and TP_5_ (Table 5; Figure 7).

**Table 5:** Tactile Braille words reading speed: post-hoc comparisons between time points.

Time point	*M*	*SD*	TP_−1_	TP_0_	TP_1_	TP_2_	TP_3_	TP_4_
TP_−1_	0.00	0.00						
TP_0_	0.00	0.00	1					
TP_1_	2.76	2.95	0.03	0.03				
TP_2_	1.94	1.85	0.01	0.01	1			
TP_3_	6.12	4.55	0.001	0.001	0.15	0.01		
TP_4_	6.76	3.75	< 0.001	< 0.001	0.006	< 0.001	1	
TP_5_	5.06	4.04	0.002	0.002	0.65	0.02	1	0.01

TP_−1_, Pre-test (1 week before the course); TP_0_, Pre-test (On the day of the course); TP_1_, 1 week into the course; TP_2_, 6 weeks into; TP_3_, 3 months into; TP_4_, 7 months into; TP_5_, Follow-up (3 months after the end of the course). p values were corrected with Bonferroni correction for multiple comparisons. *p* value was not calculated for the TP_−1_ and TP_0_ comparison due to a lack of any variability of the results—we filled the missing value with a *p*-value of 1.

### 3.2 Lexical decision task

We employed a 7 (time) x 2 (condition) rmANOVA to analyze the behavioral data for each Lexical Decision Task.

**Figure 8:**
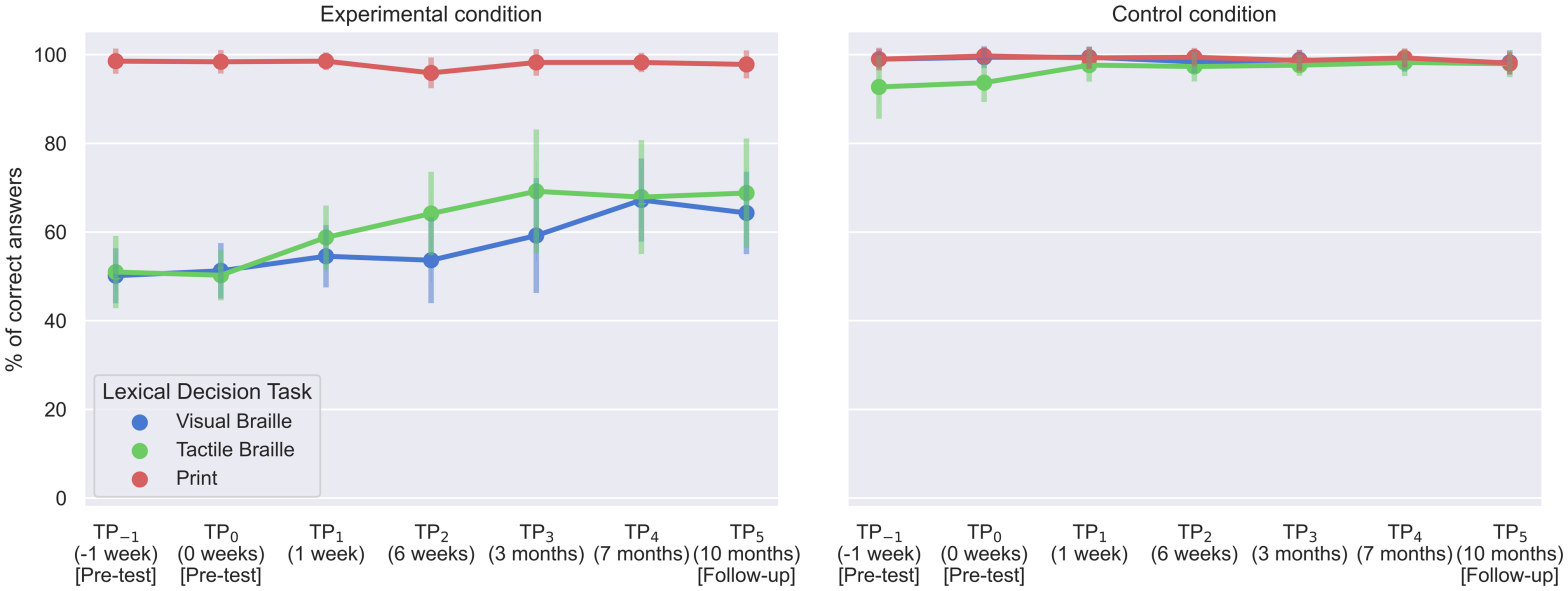
Accuracy of experimental group responses in Lexical Decision Tasks. Error bars represent standard deviations adjusted for within-subject designs (Cousineau, 2005); TP, time point.

For the Print LDT, a significant interaction between time and condition was observed [*F* (6, 96) = 3.70; *p* = 0.002]. Bonferroni pairwise comparison indicated that the experimental block correctness at TP_2_ was significantly lower than TP_−1_ and TP_5_ (Table 6; Figure 8). However, performance was at the ceiling level in all TPs, ranging from 95.88 to 98.53%. The control condition showed no significant time differences.

**Table 6:** Print Lexical Decision Task accuracy in the experimental condition: post-hoc comparisons between time points.

Time point	*M*	*SD*	TP_−1_	TP_0_	TP_1_	TP_2_	TP_3_	TP_4_
TP_−1_	98.53	2.18						
TP_0_	98.38	1.75	1					
TP_1_	98.53	1.78	1	1				
TP_2_	95.88	3.64	0.02	0.35	0.08			
TP_3_	98.24	2.30	1	1	1	0.65		
TP_4_	98.24	2.12	1	1	1	0.14	1	
TP_5_	97.79	3.29	1	1	1	0.005	1	1

TP_−1_, Pre-test (1 week before the course); TP_0_, Pre-test (On the day of the course); TP_1_, 1 week into the course; TP_2_, 6 weeks into; TP_3_, 3 months into; TP_4_, 7 months into; TP_5_, Follow-up (3 months after the end of the course). p values were corrected with Bonferroni correction for multiple comparisons.

For the Visual Braille LDT, the interaction was also significant [*F* (6, 96) = 9.45; *p* < 0.001]. The experimental task correctness was notably higher at TP_4_ than at TP_−1_, TP_0_, TP_1_, and TP_2_ and increased further at TP_5_ compared to TP_−1_ and TP_0_ (Table 7; Figure 8). Again, the control condition showed consistent performance across time points.

**Table 7:** Visual Braille Lexical Decision Task accuracy in the experimental condition: post-hoc comparisons between time points.

Time point	*M*	*SD*	TP_−1_	TP_0_	TP_1_	TP_2_	TP_3_	TP_4_
TP_−1_	50.14	5.37						
TP_0_	51.26	5.12	1					
TP_1_	54.53	7.53	1	1				
TP_2_	53.63	10.57	1	1	1			
TP_3_	59.19	14.06	0.61	1	1	1		
TP_4_	67.21	10.30	< 0.001	0.001	0.015	0.03	0.13	
TP_5_	64.29	9.60	0.006	0.004	0.25	0.09	1	1

TP_−1_, Pre-test (1 week before the course); TP_0_, Pre-test (On the day of the course); TP_1_, 1 week into the course; TP_2_, 6 weeks into; TP_3_, 3 months into; TP_4_, 7 months into; TP_5_, Follow-up (3 months after the end of the course). *p* values were corrected with Bonferroni correction for multiple comparisons.

Finally, in the Tactile Braille LDT, the interaction effect was significant [*F* (2.65, 42.33) = 5.67; *p* = 0.003]. The experimental task showed decreased correctness at TP_−1_ compared to TP_2_, TP_3_, TP_4_, and TP_5_. Additionally, TP_0_ had lower correctness than TP_1_, TP_2_, TP_3_, TP_4_, and TP_5_. In contrast, the control condition presented higher correctness at TP_4_ than at TP_0_ (Tables 8, 9; Figure 8).

**Table 8:** Tactile Braille Lexical Decision Task accuracy in the experimental condition: post-hoc comparisons between time points.

Time point	*M*	*SD*	TP_−1_	TP_0_	TP_1_	TP_2_	TP_3_	TP_4_
TP_−1_	50.96	8.41						
TP_0_	50.25	5.69	1					
TP_1_	58.77	7.92	0.10	0.007				
TP_2_	64.16	10.27	0.01	0.01	1			
TP_3_	69.18	15.46	0.02	0.006	0.21	1		
TP_4_	67.87	14.64	0.01	0.003	0.42	1	1	
TP_5_	68.79	13.74	0.02	0.002	0.21	1	1	1

TP_−1_, Pre-test (1 week before the course); TP_0_, Pre-test (On the day of the course); TP_1_, 1 week into the course; TP_2_, 6 weeks into; TP_3_, 3 months into; TP_4_, 7 months into; TP_5_, Follow-up (3 months after the end of the course). *p* values were corrected with Bonferroni correction for multiple comparisons.

**Table 9:** Tactile Braille Lexical Decision Task accuracy in the control condition: post-hoc comparisons between time points.

Time point	*M*	*SD*	TP_−1_	TP_0_	TP_1_	TP_2_	TP_3_	TP_4_
TP_−1_	92.71	7.87						
TP_0_	93.67	4.85	1					
TP_1_	97.63	2.74	0.40	0.36				
TP_2_	97.32	3.66	0.14	0.09	1			
TP_3_	97.62	2.13	0.39	0.17	1	1		
TP_4_	98.23	1.73	0.29	0.04	1	1	1	
TP_5_	97.93	2.39	0.24	0.09	1	1	1	1

TP_−1_, Pre-test (1 week before the course); TP_0_, Pre-test (On the day of the course); TP_1_, 1 week into the course; TP_2_, 6 weeks into; TP_3_, 3 months into; TP_4_, 7 months into; TP_5_, Follow-up (3 months after the end of the course). *p* values were corrected with Bonferroni correction for multiple comparisons.

### 3.3 6-dots detection task

We employed a 6 (time) x 2 (group) mixed ANOVA to analyze the behavioral data (number of correctly detected 6-dots) in the DD6 task.

Only the effect of time has reached statistical significance, *F* (2.4, 81.54) = 7.12; *p* < 0.001.

We observed a statistically significant increase in correctly detected 6-dots in the DD6 task. The experimental task showed increased correctness at TP_1_, TP_2_, TP_3_, TP_4_, and TP_5_ compared to TP_0_. Additionally, TP_3_ had higher correctness than TP_1_ (Table 10). We observed no significant effect of group [*F* (1, 34) = 3.20; *p* = 0.08] and group and time interaction [*F* (2.4, 81.54) = 0.75; *p* = 0.50]. These results indicate no behavioral differences in the DD6 between the Braille learning group and passive controls.

**Table 10:** Post-hoc comparisons between time points in the number of correctly detected stimuli in the 6-dots detection task.

Time point	*M*	*SD*	TP_0_	TP_1_	TP_2_	TP_3_	TP_4_
TP_0_	81.28	14.46					
TP_1_	88.36	11.39	0.002				
TP_2_	89.29	9.37	< 0.001	0.64			
TP_3_	91.78	8.26	< 0.001	0.003	0.40		
TP_4_	88.89	9.44	0.002	1	1	0.24	
TP_5_	87.85	11.93	0.03	1	1	0.25	1

TP_0_, Pre-test (On the day of the course); TP_1_, 1 week into the course; TP_2_, 6 weeks into; TP_3_, 3 months into; TP_4_, 7 months into; TP_5_, Follow-up (3 months after the end of the course). *p* values were corrected with Bonferroni correction for multiple comparisons.

### 3.4 Cognitive tasks

First, we computed a 2 (group) x 2 (condition) x 3 (time) mixed ANOVA for the Stroop task. Only the effect of time has reached statistical significance, *F* (2, 68) = 3.52; *p* = 0.035. We observed a statistically significant decrease in general accuracy in the Stroop task (Table 11). Accuracy was lower at TP_5_ (*M* = 0.972; *SD* = 0.04) than at TP_0_ (*M* = 0.98; *SD* = 0.02). The effects of group [*F* (1, 34) = 0.57; *p* = 0.46], block (ink, color name) [*F* (1, 34) = 1.03; *p* = 0.32], group and time interaction [*F* (2, 68) = 0.12; *p* = 0.89], group and block interaction [*F* (1, 34) = 0.05; *p* = 0.83], time and block interaction [*F* (1.69, 57.5) = 0.21; *p* = 0.77] and group, time and block interaction [*F* (1.69, 57.5) = 1.78; *p* = 0.18] were not significant. For detailed descriptive statistics, please see Supplementary Table S1.

**Table 11:** Stroop task general accuracy: post-hoc comparisons between time points.

Time point	*M*	*SD*	TP_0_	TP_4_
TP_0_	0.982	0.02		
TP_4_	0.978	0.02	0.70	
TP_5_	0.972	0.04	0.03	0.30

TP_0_, Pre-test (On the day of the course); TP_1_, 1 week into the course; TP_4_, 7 months into; TP_5_, Follow-up (3 months after the end of the course). *p* values were corrected with Bonferroni correction for multiple comparisons.

Next, we computed a 2 (group) x 3 (condition) x 3 (time) mixed ANOVA for the n-back task. Only two effects have reached statistical significance: Expectedly, in the effect of the condition [*F* (1.68, 50.38) = 225.05; *p* < 0.001], the performance was the highest in the 1-back task (*M* = 0.97; *SD* = 0.08). It was significantly lower in both 2-back (*M* = 0.76; *SD* = 0.18) and 3-back tasks (*M* = 0.50; *SD = 0.21*) (Table 12). In the effect of time [*F* (2, 60) = 6.21; *p* = 0.004], the performance was higher in TP_5_ (*M* = 0.76; *SD* = 0.23) and TP_4_ (*M* = 0.76; *SD* = 0.25) than in TP_0_ (*M* = 0.70; *SD* = 0.27) (Table 13). The effects of the group [*F* (1, 30) = 1.42; *p* = 0.24], group and condition interaction [*F* (1.68, 50.38) = 0.45; *p* = 0.61], group and time interaction [*F* (2, 60) = 0.19; *p* = 0.83], condition and time interaction [*F* (4, 120) = 1.33; *p* = 0.26], and group, condition and time interaction [*F* (4, 120) = 0.55; *p* = 0.70] were not statistically significant. For detailed descriptive statistics, please see Supplementary Table S2.

**Table 12:** N-back task general accuracy: post-hoc comparisons between conditions.

Condition	*M*	*SD*	1-back	2-back
1-back	0.96	0.08		
2-back	0.75	0.17	< 0.001	
3-back	0.50	0.21	< 0.001	< 0.001

*p values were corrected with Bonferroni correction for multiple comparisons.*

**Table 13:** N-back task general accuracy: post-hoc comparisons between time points.

Time point	*M*	*SD*	TP_0_	TP_4_
TP_0_	0.70	0.27		
TP_4_	0.76	0.25	0.007	
TP_5_	0.76	0.23	0.001	1

TP_0_, Pre-test (On the day of the course); TP_1_, 1 week into the course; TP_4_, 7 months into; TP_5_, Follow-up (3 months after the end of the course). *p* values were corrected with Bonferroni correction for multiple comparisons.

## 4 fMRI results

### 4.1 Lexical decision task

#### 4.1.1 Task repetition effect

Separately, for every LDT, we computed a paired *t*-test comparison of contrasts for the two pre-training time points (TP_0_ > TP_−1_) to control the repetition effect without learning. The analysis revealed no significant activity differences between the time points in any of the Lexical Decision Tasks, regardless of modality and alphabet.

#### 4.1.2 Main effect of time

##### 4.1.2.1 Print

We calculated a one-way rmANOVA to control the repetition effect of performing the Print LDT during the learning process. The analysis revealed no significant effect of time.

Next, we checked the main effects of time and pairwise comparisons in Braille LDTs to examine at which stage the reading network becomes involved in visual and tactile Braille reading.

##### 4.1.2.2 Visual Braille

**Figure 9:**
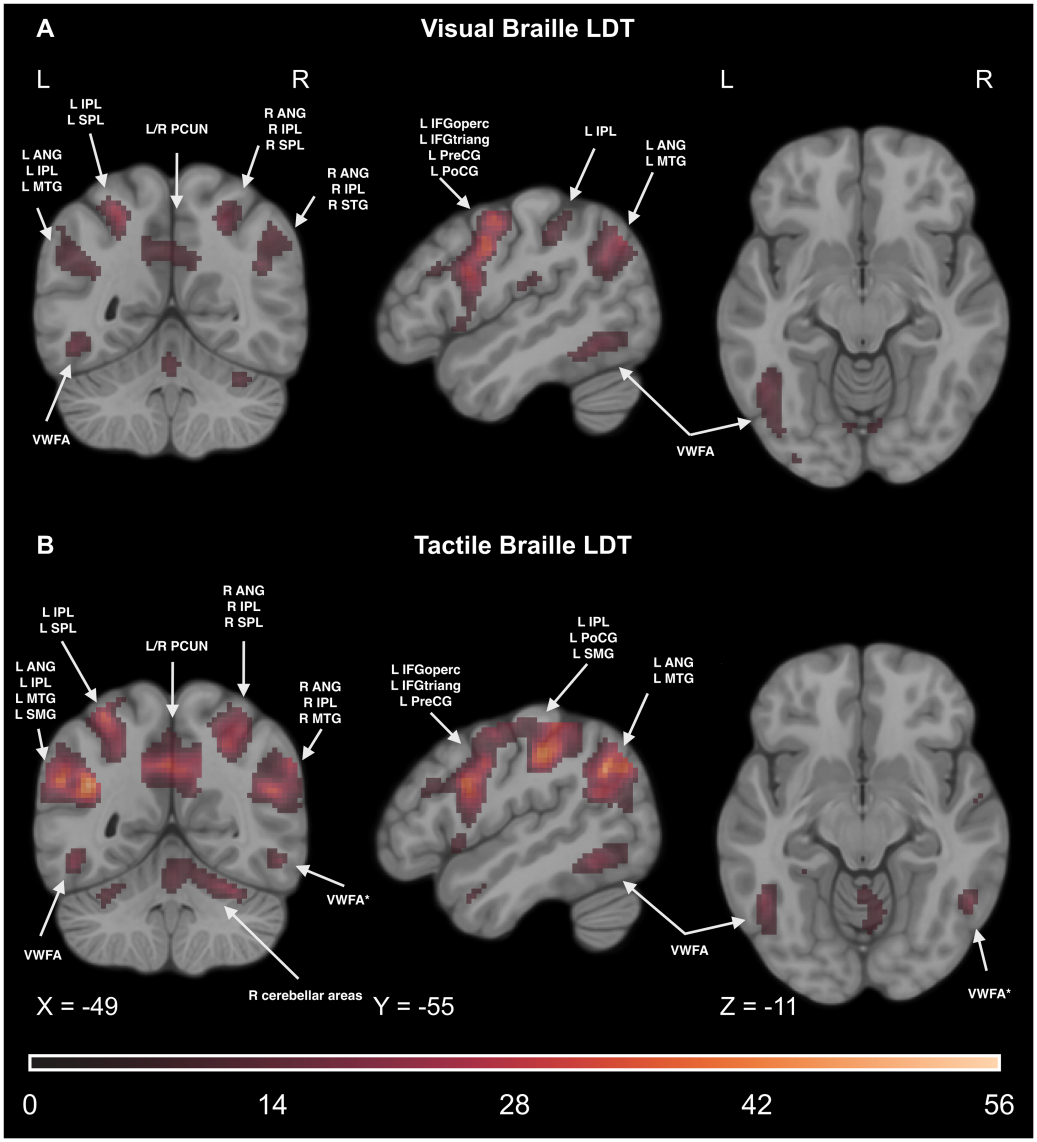
Statistical map of the main effect of time in the experimental > control comparison of the **(A)** visual Braille Lexical Decision Task (LDT) and **(B)** tactile Braille Lexical Decision Task. The colormap represents the *F-*statistic range. We used the same coordinates to visualize and facilitate comparison between images. Results were thresholded at a voxel level with a Family-Wise Error (FWE) comparisons correction with a *p*-value of 0.05 and a cluster extent of 20 voxels. ANG, Angular Gyrus; IPL, Inferior Parietal Lobule; MTG, Middle Temporal Gyrus; SMG, Supramarginal Gyrus; SPL, Superior Parietal Lobule; PCUN, Precuneus; VWFA, Visual Word Form Area; VWFA*, VWFA’s anatomical equivalent in the right hemisphere; IFGoperc, Pars Opercularis of the Inferior Frontal Gyrus; IFGtriang, Pars Triangularis of the Inferior Frontal Gyrus; PreCG, Precentral Gyrus; PoCG, Postcentral Gyrus.

**Figure 10:**
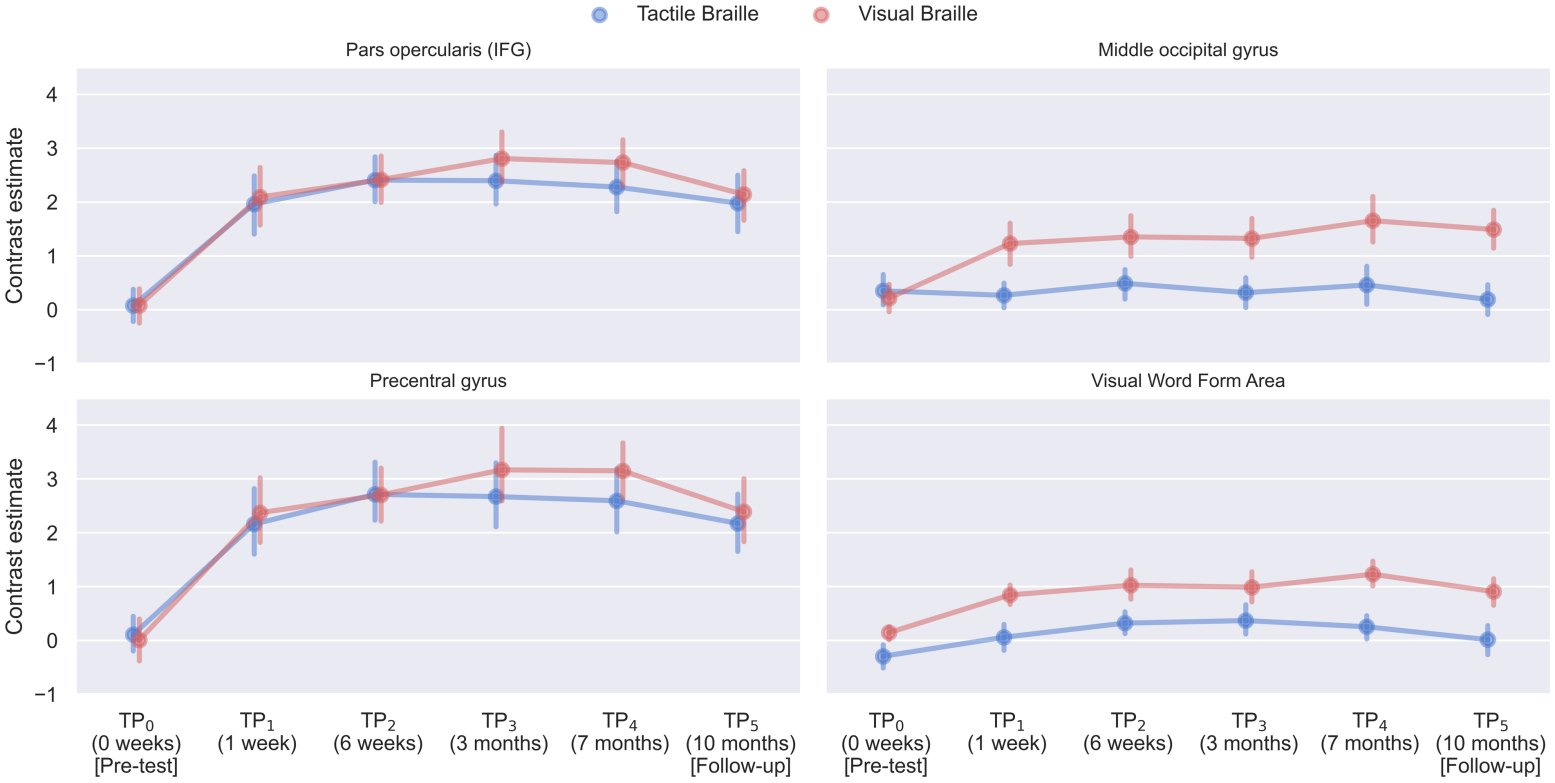
The line plots visualize the time course of activity in four peak voxels - a conjunction of activity in the main effect of condition (experimental > control) in Visual and Tactile Lexical Decision Tasks; TP, time point; L, left.

We computed a one-way rmANOVA on the group level to test which regions changed the activity level throughout the course during the visual reading task. We observed significant effects in the reading network (including the IFG and VWFA), motor network [including the supplementary motor area (SMA)], and parietal network [including the angular gyrus (ANG) and superior parietal lobule (SPL)]. For detailed results, please see Figures 9, 10 and Supplementary Table S3.

##### 4.1.2.3 Tactile Braille

We computed a one-way repeated measures ANOVA on the group level to test which regions changed the activity level throughout the course during the tactile reading task. We observed significant effects in the somatosensory network (including the postcentral gyrus (PoCG) and SPL), reading network (including the IFG and VWFA), motor network (including the SMA), and cerebellar network [including lobules VI (CER6) and VIIB (CER7B)]. Please see Figures 9, 10 and Supplementary Table S4 for detailed results.

#### 4.1.3 Pairwise comparisons between time points

Since our main interest revolved around the earliest stages of training, to test which regions changed the activity level during the Braille reading tasks after 7 days and 6 weeks of learning Braille, we computed paired t-tests using the TP1 > TP0 and TP2 > TP0 contrasts, respectively.

##### 4.1.3.1 Visual Braille

In the Visual Braille Lexical Decision Task (LDT), after 7 days of learning Braille, we observed a significant increase in brain activity in various networks, such as the reading network (comprising the IFG and VWFA), the motor network [including the SMA and precentral gyrus (PreCG)], and the parietal network [encompassing the SPL and inferior parietal lobule (IPL)]. Additionally, there was increased activity in the cerebellar network (CER6) and the insula (INS).

**Figure 11:**
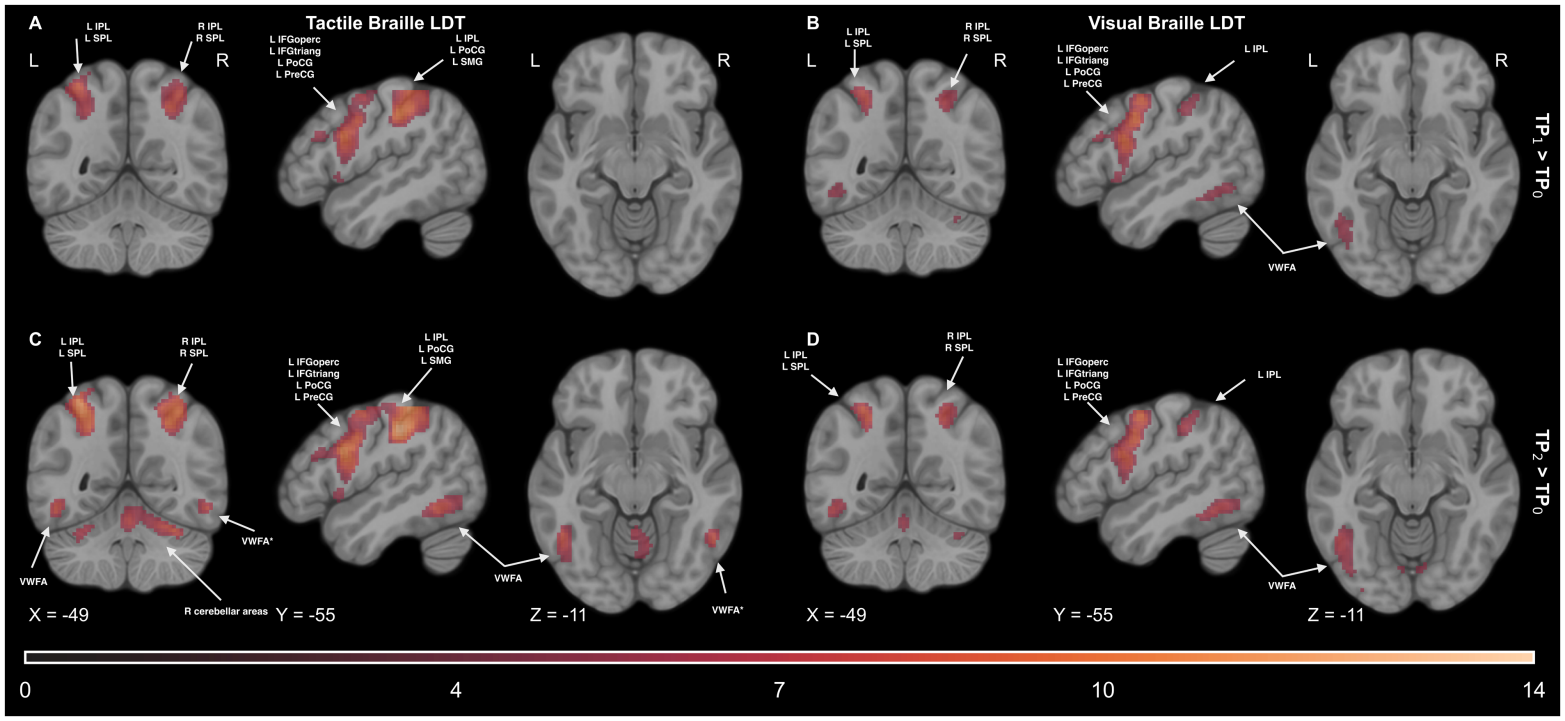
Statistical map of the experimental > control comparison of the **(A)** tactile Braille Lexical Decision Task (LDT) after 7 days of learning (TP_1_ > TP_0_), **(B)** visual Braille Lexical Decision Task (LDT) after 7 days of learning (TP_1_ > TP_0_), **(C)** tactile Braille Lexical Decision Task (LDT) after 6 weeks of learning (TP_2_ > TP_0_) and **(D)** visual Braille Lexical Decision Task (LDT) after 6 weeks of learning (TP_2_ > TP_0_). The colormap represents the *t-*statistic range. We used the same coordinates to visualize and facilitate comparison between images. Results were thresholded at a voxel level with a Family-Wise Error (FWE) comparisons correction with a p-value of 0.05 and a cluster extent of 20 voxels. IPL, Inferior Parietal Lobule; MTG, Middle Temporal Gyrus; SMG, Supramarginal Gyrus; SPL, Superior Parietal Lobule; VWFA, Visual Word Form Area; VWFA*, VWFA’s anatomical equivalent in the right hemisphere; IFGoperc, Pars Opercularis of the Inferior Frontal Gyrus; IFGtriang, Pars Triangularis of the Inferior Frontal Gyrus; PreCG, Precentral Gyrus; PoCG, Postcentral Gyrus.

After 6 weeks, a similar pattern was observed with significant effects in the calcarine cortex (CAL), motor network (SMA and PreCG), parietal network (IPL and SPL), reading network (IFG and VWFA), and cerebellar network [including lobules IV (CER4), V (CER5), VI (CER6), and VIII (CER8)]. Please see Figure 11 and Supplementary Tables S5, S6 for detailed results. For other comparisons between the remaining time points, please see Supplementary Tables S7–S9.

##### 4.1.3.2 Tactile Braille

In the Tactile Braille LDT, 7 days of learning Braille resulted in significant changes in activity within the somatosensory network (including the PoCG and SPL), the motor network (including the SMA and PreCG), and the cerebellar network (CER6). Additional significant activations were observed in the thalamus (THA) and INS.

After 6 weeks, significant effects expanded to include the caudate (CAU), somatosensory network, motor network (SMA and PreCG), parietal network (IPL and SPL), reading network (including the VWFA in the left hemisphere), and cerebellar network (including CER4, CER5, CER6, and CER8). For detailed results, please see Figure 11 and Supplementary Tables S10, S11. For other comparisons between the remaining time points, please see Supplementary Tables S12–S14.

#### 4.1.4 Braille-general and modality-specific activations

In the next series of analyses, we wanted to verify the specific brain networks engaged in tactile and visual Braille reading and which regions are involved in Braille word processing regardless of the presentation domain.

##### 4.1.4.1 Braille-general activations

**Figure 12:**
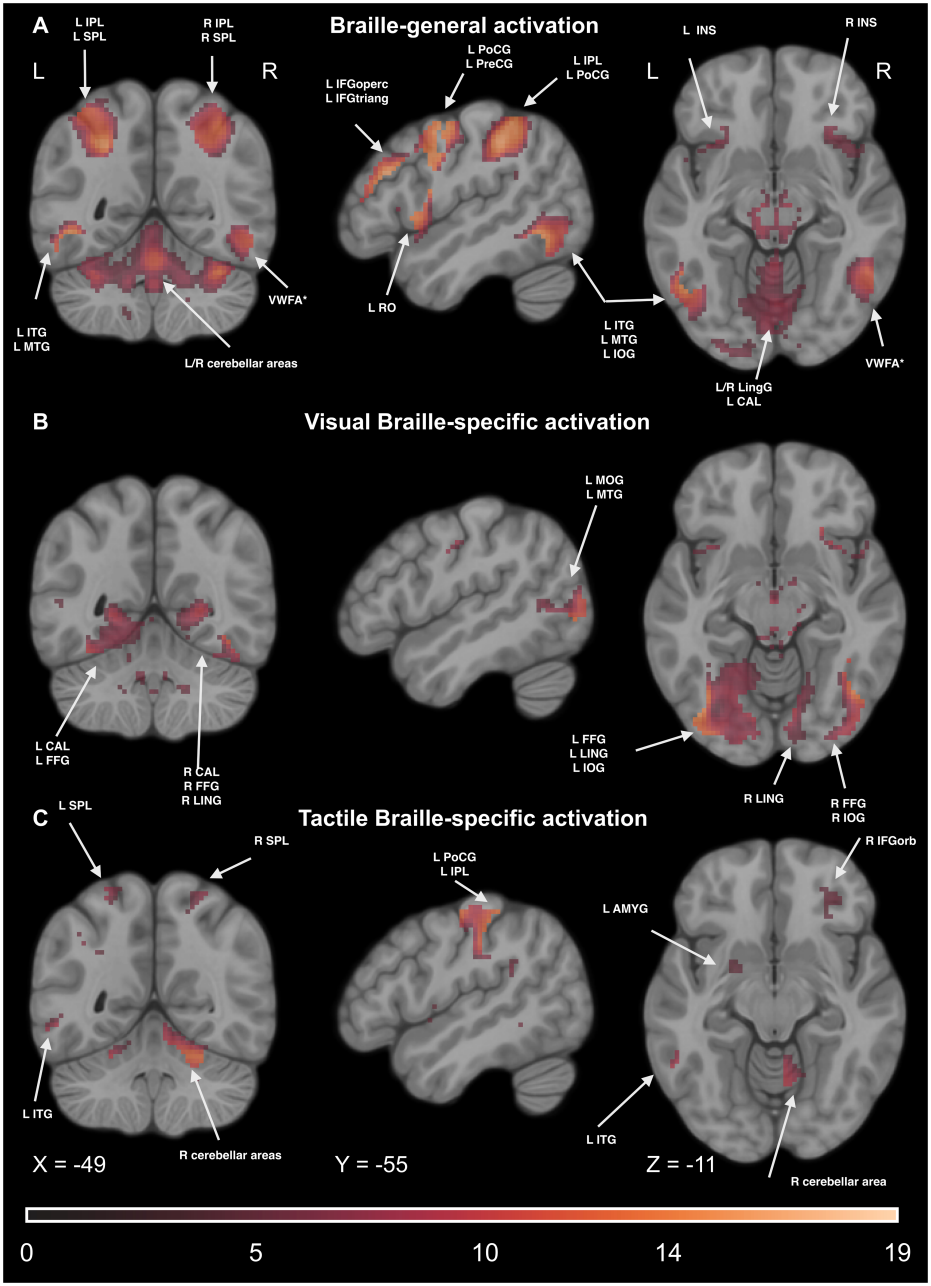
Statistical map of **(A)** Braille-general, **(B)** Visual Braille-specific, **(C)** Tactile Braille-specific activations in the Lexical Decision Tasks in the main effect of condition (experimental > control). The color map represents the *t-*statistic range. We used the same coordinates to visualize and facilitate comparison between images. Results were thresholded at a voxel level with a Family-Wise Error (FWE) comparisons correction with a p-value of 0.05 and a cluster extent of 20 voxels. IPL, Inferior Parietal Lobule; ITG, Inferior Temporal Gyrus; MTG, Middle Temporal Gyrus; SMG, Supramarginal Gyrus; SPL, Superior Parietal Lobule; VWFA, Visual Word Form Area; VWFA*, VWFA’s anatomical equivalent in the right hemisphere; IFGoperc, Pars Opercularis of the Inferior Frontal Gyrus; IFGorb, Pars Orbitalis of the Inferior Frontal Gyrus; IFGtriang, Pars Triangularis of the Inferior Frontal Gyrus; PreCG, Precentral Gyrus; PoCG, Postcentral Gyrus; RO, Rolandic Operculum; INS, Insula; Ling, Lingual Gyrus; FFG, Fusiform Gyrus; IOG, Inferior Occipital Gyrus; ANG, Angular Gyrus; AMYG, Amygdala.

We computed the conjunction of the main effects of the condition in Tactile and Visual Braille LDT to check which regions activate during the Braille tasks. We extracted voxels specific to Print reading by excluding any active voxels from the Print LDT. We observed significant effects in the motor network (including the SMA and PreCG), reading network [including the IFG, VWFA, and inferior temporal gyrus (ITG)], parietal network (including the IPL, SPL, supramarginal gyrus (SMG), and ANG), somatosensory network (including the PoCG), and cerebellar network (including CER6 and CER8). Additionally, significant effects were observed in other regions, such as the INS, THA, CAU, and anterior cingulate gyrus (ACG). For detailed results, please see Figure 12 and Supplementary Table S15.

##### 4.1.4.2 Visual Braille

We computed the main effect of the condition to check which regions activate during the Visual Braille task. We extracted voxels specific to visual Braille reading by excluding active voxels from the Print or the Tactile Braille LDT. We observed significant effects in the visual network [including the middle occipital gyrus (MOG), inferior occipital gyrus (IOG), CAL, and fusiform gyrus (FFG)], the reading network (including the IFG, VWFA, and ITG), the motor network (including the SMA and PreCG), and the cerebellar network [including lobule III (CER3), CER7, and lobule IX (CER9) of the cerebellar hemisphere, and vermis (VER)]. Additionally, significant effects were observed in the parietal network (including the SPL) and other regions, such as the INS, THA, CAU, and ACG. For detailed results, please see Figure 12 and Supplementary Table S16.

##### 4.1.4.3 Tactile Braille

Analogously, in the Tactile Braille Lexical Decision Task (LDT), we computed the main effect of the condition excluding voxels active in Visual Braille and Print LDTs. We observed significant effects in the somatosensory network (including the PoCG and SPL), motor network (including the SMA and PreCG), parietal network (including the IPL and SMG), reading network (including the IFG and ITG), and cerebellar network (including CER6, CER9, and VER). Additionally, significant effects were observed in other regions, such as the INS, THA, CAU, putamen (PUT), pallidum (PAL), and ACG. For detailed results, please see Figure 12 and Supplementary Table S17.

### 4.2 6-dots detection task

**Figure 13:**
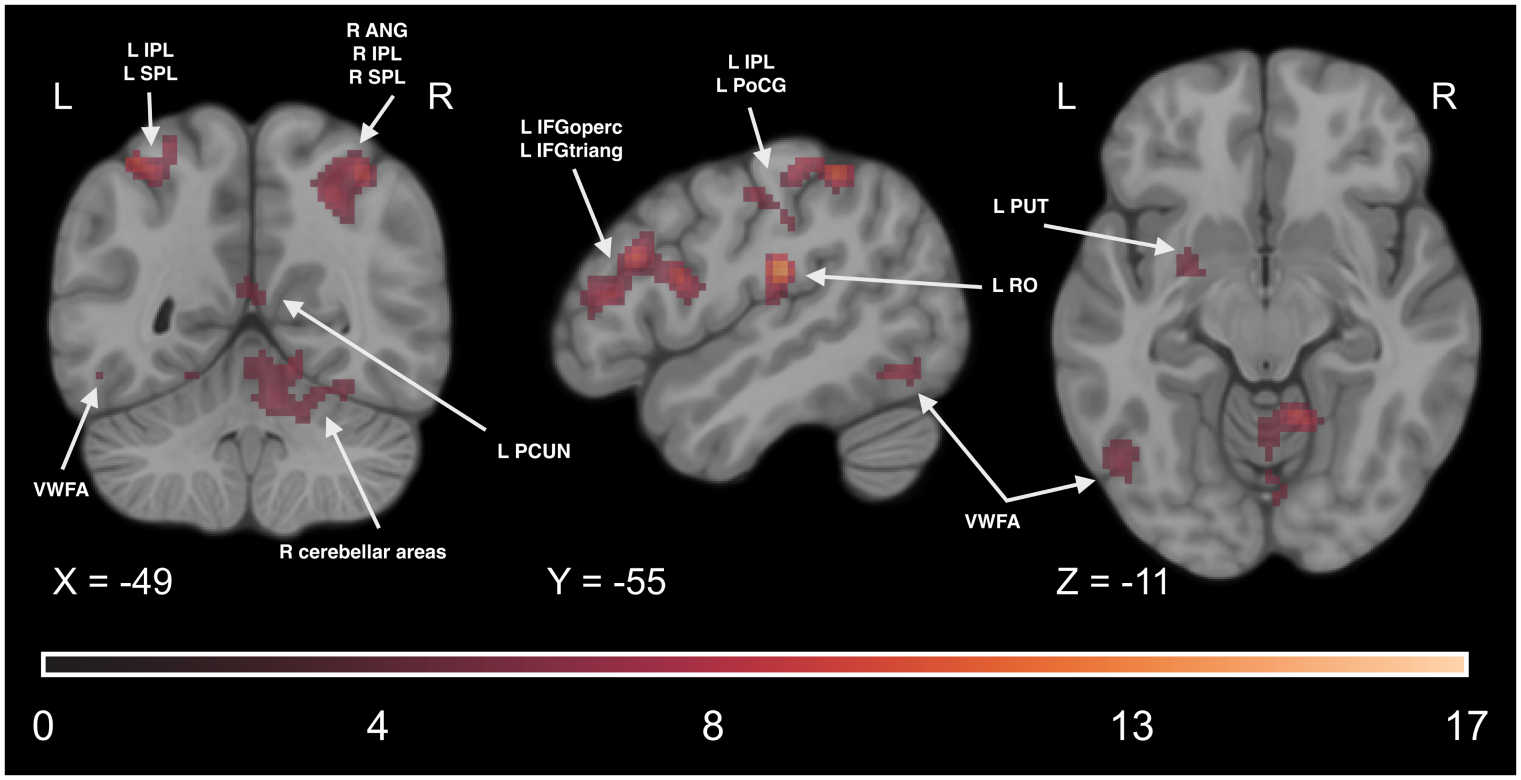
Statistical map of the main effect of group (experimental > control contrast) in the DD6 task. The colormap represents the *t-*statistic range. We used the coordinates of the peak activity in Braille Lexical Decision tasks to visualize and facilitate comparison between images. Results were thresholded at a voxel level with a Family-Wise Error (FWE) comparisons correction with a p-value of 0.05 and a cluster extent of 20 voxels. ANG, Angular Gyrus; IPL, Inferior Parietal Lobule; SPL, Superior Parietal Lobule; VWFA, Visual Word Form Area; IFGoperc, Pars Opercularis of the Inferior Frontal Gyrus; IFGtriang, Pars Triangularis of the Inferior Frontal Gyrus; PoCG, Postcentral Gyrus; RO, Rolandic Operculum; PUT, Putamen; PCUN, Precuneus.

Finally, to examine if the involvement of the reading network is observed during implicit Braille reading, we conducted a series of analyses of fMRI data collected during the DD6 task. We computed a paired t-test comparison of the experimental condition between experimental and control groups averaged across all TPs. We observed a statistically significant higher activity level in the experimental group in several regions. These regions include the motor network (such as the PreCG), reading network (including the IFG and VWFA), parietal network (including the SMG, IPL, and SPL), somatosensory network (including the PoCG), and cerebellar network (including CER4, CER5, CER6). Additionally, significant effects were observed in other regions such as the INS, PUT, middle frontal gyrus (MFG), superior frontal gyrus (SFG), superior temporal gyrus (STG), and THA. For detailed results, please see Figure 13 and Supplementary Table S18.

We found no significant main effect of time or time-by-group interaction.

Even though the group and time interaction effect was not significant, we decided to conduct two additional analyses to see potential differences in activity between the experimental and control groups: one before the learning (at TP_0_) and one throughout the learning process by pooling together all timepoints where the experimental group underwent the training (from TP_1_ to TP_4_). Before the training, the only regions with higher activity in the experimental group compared to controls were the PoCG and SMG in the right hemisphere. For detailed results, please see Supplementary Table S19.

**Figure 14:**
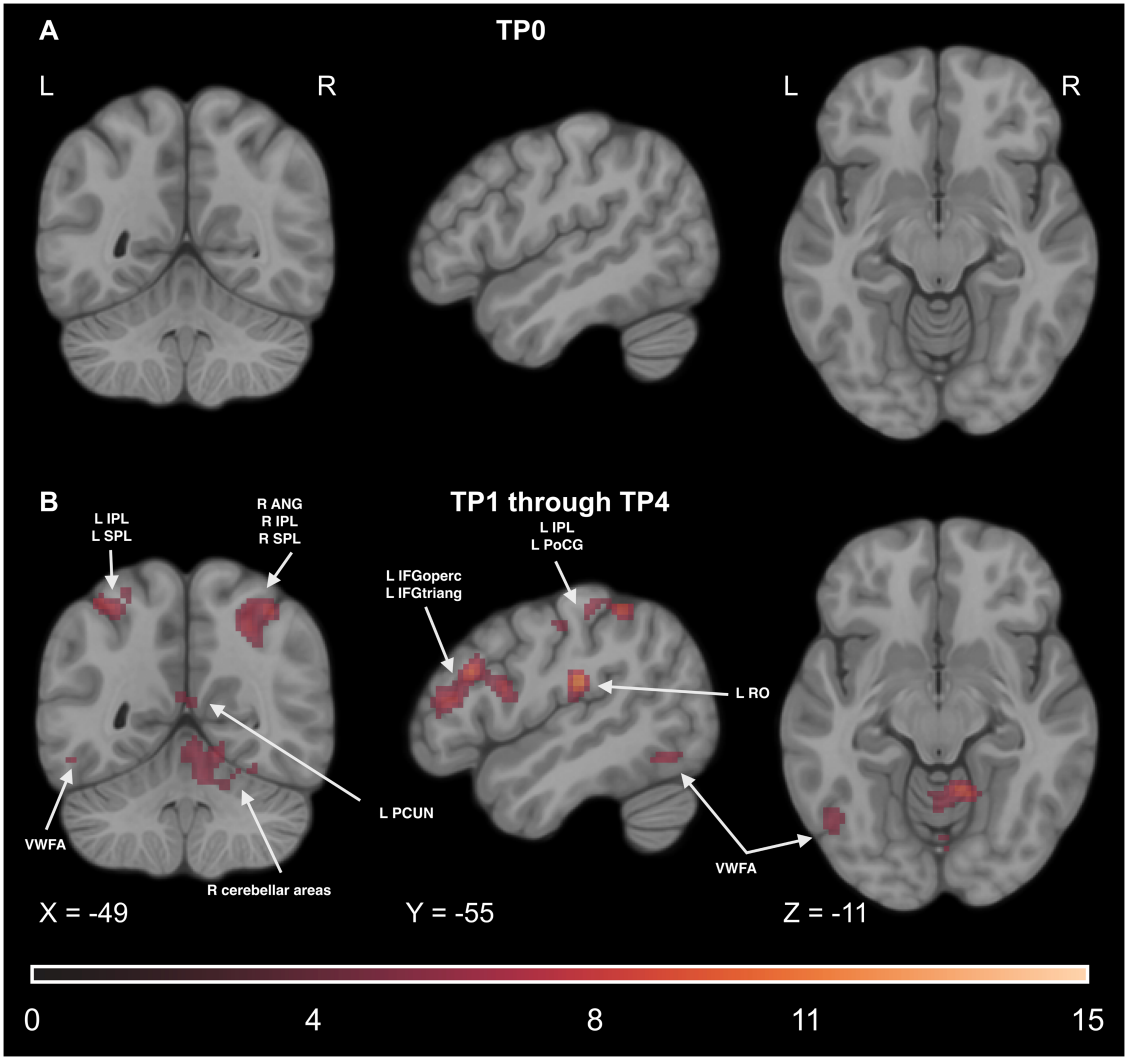
Statistical maps of the experimental > control groups comparison **(A)** before the learning process began in the experimental group (at TP_0_), **(B)** throughout the learning process, at all time points between TP_1_ and TP_4_ pooled together. The color map represents the *t-*statistic range. We used the coordinates of the peak activity in Braille Lexical Decision tasks to visualize and facilitate comparison between images. Results were thresholded at a voxel level with a Family-Wise Error (FWE) comparisons correction with a p-value of 0.05 and a cluster extent of 20 voxels. ANG, Angular Gyrus, IPL, Inferior Parietal Lobule, SPL, Superior Parietal Lobule, VWFA, Visual Word Form Area, IFGoperc, Pars Opercularis of the Inferior Frontal Gyrus, IFGtriang, Pars Triangularis of the Inferior Frontal Gyrus, PoCG, Postcentral Gyrus, RO, Rolandic Operculum, PCUN, Precuneus.

The comparison of all the training time points pooled together revealed significant effects in the motor network (including the PreCG), reading network (including the IFG and the VWFA), parietal network (including the IPL, SPL, and SMG), somatosensory network (including the PoCG), and cerebellar network (including CER4, CER5, and CER6). Additional significant effects were observed in regions such as the INS, PUT, MFG, SFG, STG, FFG, paracentral lobule (PCL), and the THA. For detailed results, please see Figure 14 and Supplementary Table S20.

## 5 Discussion

The current research builds upon prior studies that have demonstrated functional reorganization of the reading network, including the VWFA, during tactile Braille learning in proficient visual Braille users (Siuda-Krzywicka et al., 2016; Matuszewski et al., 2021). Uniquely, this study is one of the first attempts to understand the early stage of functional neuroplasticity in individuals without any previous visual or tactile Braille experience. Here, we demonstrate that functional reorganization related to Braille learning in sighted individuals can occur within the first week of learning.

### 5.1 Early signs of neuroplasticity in reading and visual networks

Our research explored the neural mechanisms involved in Braille reading and detection tasks among sighted individuals, focusing on integrating tactile and higher-order cognitive processes. Using functional magnetic resonance imaging (fMRI), we revealed distinct activation patterns across various brain regions of reading and visual networks, such as the inferior frontal gyrus (IFG), middle occipital gyrus (MOG), and visual word form area (VWFA), emphasizing the complexity and specificity of sensory and cognitive integration required for tactile reading. To answer our first research question—at which stage does the reading network become involved in visual and tactile Braille reading?—we introduced two main repeated measures ANOVA models for each Braille Lexical Decision Task (Visual and Tactile). We used experimental > control condition contrast to find regions that increase the activity attributed to lexical reading throughout the Braille learning process.

#### 5.1.1 Inferior frontal gyrus (IFG)

We found significant increases in activity within the opercular and triangular parts of the IFG during both Visual and Tactile Braille Lexical Decision Tasks (LDT). The left IFG_operc_ showed increased activity in both tasks after 7 days of Braille learning, persisting throughout the study and at a three-month follow-up. Interestingly, while the right IFG_operc_ also showed increased activity during the Tactile Braille LDT, its engagement did not continue during the Visual Braille LDT at the follow-up. The behavioral results show a decline in both letter and word reading skills after a break from learning, indicating that the neural mechanisms supporting these skills may also exhibit reduced activation after a period of non-use.

In the left hemisphere, the IFG_operc_ primarily involves phonological processing and detailed linguistic control (Vigneau et al., 2006). The IFG_triang_, particularly its dorsal part, is associated with phonological working memory, while its ventral part is engaged in semantic processing and integration of complex auditory and linguistic information (Poldrack et al., 2001; Vigneau et al., 2006). These regions integrate complex auditory and linguistic information, facilitating dynamic auditory processing and phonological discrimination. A rapid increase of activity in both those areas in visual and tactile Braille underscores the left hemisphere’s specialization in language, phonological processing, and cognitive control, which is crucial for interpreting any script, regardless of kind and presentation domain (Cornelissen et al., 2009; Kinno et al., 2014).

The activity of the right IFG_operc_ in visual and tactile Braille reading could be explained by a mechanism similar to one in young children. During the early stages of reading acquisition, a compensation mechanism appears, and increased activity in the right hemisphere can be observed during reading (Margolis et al., 2020). Unlike the right IFG_operc_’s compensatory role in unskilled readers, the right IFG_triang_ is often involved in higher-order language processing, such as syntactic and semantic tasks (Sinha et al., 2024). The LDT used in this study was a reasonably straightforward task that checked the ability to distinguish real words and pseudowords correctly. As other analyses in this study indicate, this area is active in both visual and tactile Braille Lexical Decision Tasks (Supplementary Table S15). However, no increase in activity in this region throughout the training is not surprising, as no complex syntactic or semantic processing was required here.

#### 5.1.2 Middle occipital gyrus (MOG)

We observed the first brain activity reorganization related to cross-modal plasticity in the middle occipital gyrus (MOG) within the first week of Braille learning. This region is structurally linked with the IFG through the inferior frontal-occipital fasciculus (Sarubbo et al., 2013). In both hemispheres, the MOG showed increased activity during both tactile and visual Braille LDTs throughout the study, including the follow-up after a 3-month-long break from learning Braille. Previous Braille studies with sighted people have produced conflicting results regarding the involvement of the MOG during Braille reading, with one study showing no significant increase in activity during a tactile LDT in the occipital areas (Matuszewski et al., 2021) and another showing an increase in activity only in the right MOG, and only during tactile Braille reading (Siuda-Krzywicka et al., 2016). These findings are in contrast with our results, which show a bilateral, rapid, and lasting increase in activity in this area.

One possible explanation is that our study employed naive adults without any previous Braille experience, visual or tactile. In contrast, earlier studies recruited either professionals working with visual Braille daily or students in special education aiming to work with the blind in the future, all of whom were skilled in reading visual Braille. The different levels of Braille experience, or in the current study’s case, lack thereof, suggest that a representation of Braille script in the visual cortex appears within the first 7 days of tactile Braille learning. The MOG is crucial for visual processing, particularly at the early stages of visual information and spatial awareness. The increased activity in both visual and tactile Braille LDTs suggests high responsiveness to novel visual stimuli and an ability to adapt to tactile stimuli, potentially indicating cross-modal plasticity where visual areas are recruited for tactile processing (Sadato et al., 1996).

Previous research on congenitally blind adults showed that the occipital cortex, typically involved in visual processing, can be recruited for language tasks with increased functional connectivity with traditional language areas (Bedny et al., 2011). Moreover, activity in the lateral occipital complex during tactile object recognition in both blind and sighted individuals indicates that the activation of the occipital areas is not merely a result of visual imagery but can represent genuine cross-modal plasticity (Amedi et al., 2010).

#### 5.1.3 Visual word form area (VWFA)

The visual word form area (VWFA) exhibited differential activation patterns in the context of tactile and visual Braille Lexical Decision Tasks. In the left hemisphere, increased activity was observed in the visual reading task as early as after 7 days of learning and remained heightened throughout the entire study. In the tactile task, the first increase in activity could be observed after 6 weeks of Braille learning and remained throughout the entire study. In the anatomical equivalent of the VWFA in the right hemisphere (VWFA*) (Yeatman et al., 2013; Cohen et al., 2003), no activity was observed at any stage during the visual Braille task. In the tactile task, once again, activity increased after 6 weeks. However, it was only present throughout the learning phase, with no increase in activity observed in the follow-up session 3 months after completing the Braille course.

Multiple studies with blind people have shown that the VWFA is active during tactile reading (Ptito et al., 2012; Reich et al., 2011; Striem-Amit et al., 2012). Moreover, previous studies on sighted people learning tactile Braille also showed an increase in activity in this area during tactile reading (Siuda-Krzywicka et al., 2016; Matuszewski et al., 2021). Our study adds to existing evidence by showing a more detailed description of the time course of plasticity within this area. The simultaneous activation of the VWFA in both hemispheres during the tactile Braille task suggests that both hemispheres are involved in processing tactile Braille during the active learning phase. The activity of R VWFA* has yet to be interpreted. However, it has previously been reported as active in both blind and sighted people in a shape recognition task (Ptito et al., 2012), in blind people during an auditory task (Striem-Amit et al., 2012), and in sighted people reading visually false fonts (Vinckier et al., 2007). One of the previous Braille studies with sighted learners reported in the R VWFA* a significant peak of activity in tactile Braille modulated by tactile letter recognition skills (Siuda-Krzywicka et al., 2016). The involvement of the right VWFA* may reflect the additional sensory and spatial processing demands of tactile reading, which require a broader network of neural resources (Striem-Amit et al., 2012). The lack of the right VWFA* activity after the break from learning suggests that its role during the learning phase depends on continuous practice and engagement with Braille. The right VWFA*’s involvement may rely more on active engagement with the task, and its lack of increased activity post-break indicates that it only maintains its function with regular practice.

Another possibility would be an increase in activity compensation mechanism similar to the one mentioned in the right IFG_operc_. However, it does not seem probable because while IFG_operc_ was active in both tactile and visual Braille LDTs, R VWFA* was only engaged in the tactile LDT.

### 5.2 Braille-general and modality-specific activity

Our research investigates the neural foundations of Braille reading, concentrating on both general and modality-specific brain activities. We identified distinct activation patterns using fMRI across several brain regions engaged in visual and tactile Braille reading. To address our second research question—what specific brain networks are engaged in tactile and visual Braille reading?—we developed a single repeated measures ANOVA (rmANOVA) model, incorporating the Lexical Decision Task (Print, Visual Braille, and Tactile Braille [TP0 - TP5 pooled together]). To identify regions active in Braille reading regardless of the domain, we computed the conjunction of the conjunction of activity (experimental > control contrast) in Tactile and Visual Braille LDT, excluding any active voxels in the Print LDT. For regions activated specifically by tactile Braille reading, we analyzed the main effect present in the Tactile LDT, excluding voxels active in Visual Braille or Print LDT. Similarly, to pinpoint regions specific to visual Braille reading, we examined the effect in the Visual Braille LDT, excluding voxels active in Tactile Braille or Print LDT.

#### 5.2.1 Rolandic operculum (ROL)

The Rolandic operculum exhibited specific activity in both hemispheres during tactile Braille tasks, indicating its significant role in tactile processing and sensory integration necessary for reading Braille. This region is part of the secondary somatosensory cortex (S2), involved in higher-order processing of somatosensory information and coordinating motor actions. Its activation during tactile Braille tasks underscores its role in processing tactile stimuli and integrating sensory inputs with motor responses required for reading Braille by touch (Eickhoff et al., 2006).

#### 5.2.2 Medial cingulate gyrus (MedCG)

The medial cingulate gyrus, part of the cingulate cortex, exhibited specific activity in the right hemisphere during visual Braille tasks, suggesting a role in visual processing and higher-order cognitive functions associated with the task of reading Braille. The cingulate gyrus is involved in various cognitive processes, including attention, error detection, and the integration of sensory information. Its activation during visual Braille tasks highlights its role in visual attention and processing (Bush et al., 2000). The right hemisphere’s specific involvement may reflect the lateralization of spatial attention and visual processing, which is crucial for interpreting visually presented Braille characters (Corbetta and Shulman, 2002). The cingulate gyrus integrates visual inputs with higher-order cognitive functions, supporting the complex task of reading Braille visually.

#### 5.2.3 Inferior parietal lobule (IPL)

The inferior parietal lobule (IPL) exhibited Braille-general activity in both hemispheres and specific tactile activity in the left hemisphere. This pattern suggests a multifaceted role in visual and tactile processing, reflecting its involvement in integrating sensory information and coordinating complex cognitive tasks. The IPL is part of the sensory processing network. It is known for integrating multimodal sensory information, spatial orientation, and attention (Culham and Kanwisher, 2001). The specific activation in the left hemisphere during tactile Braille tasks underscores the IPL’s role in somatosensory processing and spatial attention. This region is crucial for processing tactile stimuli and integrating them with spatial and motor functions necessary for reading Braille by touch (Binkofski et al., 1999).

### 5.3 DD6: implicit reading task

Another important aspect of this study is including a passive control group to increase the reliability of our implicit reading results and make them attributable specifically to learning Braille. The DD6 task was developed to compare Braille learners with individuals who lacked knowledge of the Braille alphabet. With its linguistic component, this task was sensible for the learning group and solvable for those without Braille knowledge. Behaviorally, we observed no significant group effect and group and time interaction, indicating that the experimental group of Braille learners and the control group managed to detect 6-dots on a similar level, regardless of time. However, the fMRI results of the DD6 task revealed pronounced differences in brain activation patterns between the two groups that were not present before the learning onset. This is the first study to use a tactile implicit reading task meaningful for Braille learners and doable by people without Braille knowledge. The activation patterns observed in the experimental group emphasize the adaptability of the human brain to process written language implicitly, even using touch. These results should, however, be interpreted with some caution as no significant fMRI results in the interaction effect could be reported in our study.

#### 5.3.1 Inferior frontal gyrus (IFG) and visual word form area (VWFA)

We found a higher level of bilateral activity within the opercular and triangular parts of the IFG in the experimental group compared to passive controls. A considerable degree of similarity between the activity in the implicit reading task and activity in tactile Braille LDT, which was an explicit reading task, indicates that in sighted people learning to read Braille, tactile Braille reading, implicit or explicit, employs similar neural mechanisms as ones mentioned at the beginning of the discussion (please see Supplementary Tables S15, 17 for additional results from the Braille LDTs).

The experimental group also exhibited higher activity than the control group in the VWFA. Interestingly, no difference in activity could be observed in the anatomical equivalent of the VWFA in the right hemisphere (VWFA*). As mentioned above, while some studies report significant activity in this area in both hemispheres, the role of R VWFA* has yet to be discussed. While previous studies focusing on visual implicit reading indicated significant activity in the VWFA (Thuy et al., 2004; Price and Devlin, 2011), none reported any activity in its counterpart in the right hemisphere. The results of the VWFA/R VWFA* reported in this study align with those of previous studies. It is possible that while R VWFA* plays an active role in explicit reading in the tactile domain (such as the Lexical Decision Task) and accommodates additional sensory and spatial processing demands of conscious tactile reading, in implicit reading (such as the 6-dot Detection Task) there is no demand for support, thus the lack of difference in activity between Braille readers and passive controls.

### 5.4 Far transfer of skills

Additionally, we investigated a possible far transfer of skills in the study. We noted a significant increase in accuracy in the n-back task, while accuracy in the Stroop task decreased over time, albeit reaching nearly a ceiling effect. However, we found neither group nor group and time interaction effects in either task, leading us to conclude that the improvement is likely related to the exposure to the task itself, and no far transfer effects could be observed due to tactile training. These findings align with numerous studies and meta-analyses that have reported negligible far-transfer effects at most when the learned skill has little connection to other cognitive tasks (Sala et al., 2019).

### 5.5 Recruitment and participant engagement

Participants in the experimental group were recruited from those pursuing a degree in typhlopedagogy, a special branch of education for people working with visually impaired individuals. By recruiting such a specific group we wanted to ensure their motivation to complete the training course. In contrast, the control group was recruited more broadly without this restriction. This difference was necessary due to several practical constraints. First of all, the ongoing COVID-19 pandemic significantly impacted our ability to recruit an adequate number of participants for the experimental group. Expanding recruitment to a more diverse demographic without compromising the study’s primary focus was not feasible. To prevent potential biases, recruitment for the control group was not restricted to the pedagogical university. This strategy minimized the likelihood that control participants would become aware of their comparative role in a study related to Braille, which could influence their participation or responses.

While the experimental group’s background might have influenced their engagement and motivation, we found no significant behavioral differences between the experimental and control groups in tasks such as the 6-dot-detection task (DD6) or other out-of-scanner cognitive tasks. This suggests that engagement levels were comparable across groups, thus supporting the reliability of our findings.

Participants in both the experimental and control groups were university students who continued their regular academic activities throughout the study. This similarity in their academic engagement helps mitigate potential confounds related to differences in cognitive stimulation during the study period. Moreover, the primary objective of our research was to observe functional neuroplastic changes associated with learning Braille. Regular academic activities would not necessarily mimic the specific cognitive and sensory engagement required by Braille learning, which was the focus of our experimental intervention.

Engaging the control group in unrelated learning activities could introduce additional variables that obscure the specific learning effects we aimed to investigate. Therefore, our focus was on the specific neuroplastic changes induced by Braille learning, thereby minimizing the impact of other potential variables.

## 6 Limitations

There are some limitations to this study. The primary limitation is the small sample size, with only 17 participants in the experimental group and 19 in the passive control group. Our original plan was to recruit 30 participants for the learning group and 25 for the passive group. However, the recruitment process coincided with the global SARS-CoV-2 pandemic, which hindered our ability to fill the slots due to potential participants’ health concerns and unwillingness to participate in scientific experiments during this period. The initial sample of 21 individuals in each group was already below our intention, and not all could participate in the study, further reducing our sample size. However, using a very conservative family-wise error (FWE) correction, combined with the cluster-size correction for a minimum of 20 voxels, is more likely to result in type II errors than false positive findings. As such, it is possible that certain effects were not detected in our analyses, but it is unlikely that the small sample size resulted in a chance of a false-positive outcome.

## 7 Conclusion

The findings from the current study offer a nuanced perspective on the early stages of functional neuroplasticity during tactile Braille learning. Our data indicate that sighted people unfamiliar with visual or tactile Braille can show significant functional reorganization within the reading network after just a week of instruction, underscoring the rapid onset of cross-modal plasticity. This rapid neuroplasticity highlights the brain’s adaptability and readiness to process unfamiliar symbols as linguistic stimuli almost immediately after learning begins.

Our research further emphasizes the dynamic nature of Braille reading skills, demonstrating continuous learning advancements with an observable plateau in brain activation following the initial phases. Training enables tactile Braille to engage in typical reading areas even without explicit instruction, indicating the underlying readiness of this network to process stimuli in an atypical sensory modality. Moreover, our investigation into the potential far-transfer effects of Braille learning aligns with previous research, suggesting no effects when the learned skill does not intersect with other cognitive domains.

These insights enhance our understanding of brain plasticity and set the stage for future research. Future studies should aim to gain more knowledge in implicit tactile reading. Moreover, the functional neuroplasticity in naive adults without any Braille knowledge should be examined in an fMRI setting daily within the initial learning period to capture the rapid and dynamic changes in neural activity.

In conclusion, our research highlights the brain’s extraordinary ability to adapt to new sensory experiences through cross-modal plasticity. The observed neural changes during tactile Braille learning underscore the human brain’s flexibility.

## Data Availability

The behavioral data and thresholded statistical maps from all whole brain analyses are available in the Open Science Framework project: https://osf.io/t4652/.

## Footnotes

^1^ https://www.neurobs.com/menu_presentation/menu_teaching/pack_list
