# Supplementary material for: How learning to read Braille in visual and tactile domains reorganizes the sighted brain
Source: Front Neurosci. 2025 Jan 6;18:1297344. doi: 10.3389/fnins.2024.1297344 (PMC11744719; doi:10.3389/fnins.2024.1297344)
Supplement: Supplementary file 1 [file Data_Sheet_1.docx]

How learning to read Braille in visual and tactile domains reorganizes the sighted brain - supplementary materials

# 1 Behavioural tasks

**​​Table S1.** Descriptive statistics for task accuracy in the Stroop task, divided by group, time point and block type.

| *Group* | *Time point* | *Block* | | | |
| --- | --- | --- | --- | --- | --- |
|  |  | *Color name* | | *Ink* | |
|  |  | *M* | *SD* | *M* | *SD* |
| Experimental | TP_0_ | 0.99 | 0.02 | 0.98 | 0.02 |
|  | TP_4_ | 0.99 | 0.02 | 0.98 | 0.03 |
|  | TP_5_ | 0.97 | 0.04 | 0.97 | 0.02 |
| Control | TP_0_ | 0.98 | 0.02 | 0.98 | 0.02 |
|  | TP_4_ | 0.97 | 0.02 | 0.98 | 0.02 |
|  | TP_5_ | 0.98 | 0.02 | 0.97 | 0.05 |

**​​Table S2.** Descriptive statistics for task accuracy in the n-back task, divided by group, time point and block type.

| *Group* | *Time point* | *1-back* | | *2-back* | | *3-back* | |
| --- | --- | --- | --- | --- | --- | --- | --- |
|  |  | *M* | *SD* | *M* | *SD* | *M* | *SD* |
| Experimental | TP0 | 0.95 | 0.10 | 0.65 | 0.16 | 0.44 | 0.19 |
|  | TP4 | 0.97 | 0.10 | 0.78 | 0.20 | 0.45 | 0.21 |
|  | TP5 | 0.96 | 0.07 | 0.80 | 0.18 | 0.48 | 0.16 |
| Control | TP0 | 0.97 | 0.10 | 0.76 | 0.14 | 0.48 | 0.25 |
|  | TP4 | 0.99 | 0.03 | 0.82 | 0.13 | 0.56 | 0.22 |
|  | TP5 | 0.97 | 0.05 | 0.77 | 0.16 | 0.58 | 0.22 |

# 2 Lexical Decision Task - main effect of time

**Table S3.** The main effect of time in the Visual Braille Lexical Decision Task. All anatomical structures were labeled with the Automated Anatomical Labelling (AAL) atlas [(Rolls et al., 2020)](https://paperpile.com/c/XavzTt/10gsu). L = left, R = right.

| *Region Label* | *Cluster extent* | *F* | *x* | *y* | *z* |
| --- | --- | --- | --- | --- | --- |
| L Supplementary motor area | 618 | 41.30 | -4 | 2 | 62 |
| R Supplementary motor area |  | 21.72 | 6 | 12 | 49 |
| R Medial cingulate gyrus |  | 15.92 | 11 | 20 | 34 |
| L Precentral gyrus | 1423 | 40.95 | -46 | -2 | 36 |
| L Opercular part of the inferior frontal gyrus |  | 30.62 | -49 | 8 | 24 |
| L Insula |  | 25.80 | -32 | 28 | 4 |
| L Triangular part of the inferior frontal gyrus |  | 14.51 | -39 | 28 | 22 |
| L Angular gyrus | 543 | 39.65 | -42 | -72 | 46 |
| L Superior parietal lobule | 1256 | 29.67 | -26 | -62 | 56 |
| L Middle occipital gyrus |  | 24.12 | -29 | -75 | 24 |
| L Inferior parietal lobule |  | 20.78 | -39 | -45 | 46 |
| L Inferior occipital gyrus |  | 11.87 | -36 | -90 | -8 |
| R Lobule VI of cerebellar hemisphere | 499 | 26.95 | 28 | -68 | -24 |
| L Lobule VI of cerebellar hemisphere |  | 16.80 | -9 | -75 | -16 |
| L Lobule VII of vermis |  | 13.71 | -2 | -75 | -26 |
| R Angular gyrus | 443 | 23.72 | 44 | -68 | 36 |
| R Inferior parietal lobule |  | 14.88 | 54 | -58 | 44 |
| R Insula | 308 | 18.25 | 34 | 18 | 9 |
| R Superior parietal lobule | 466 | 21.02 | 24 | -65 | 56 |
| R Superior occipital gyrus |  | 17.54 | 28 | -78 | 32 |
| L Medial cingulate gyrus | 554 | 20.25 | -4 | -48 | 34 |
| R Medial cingulate gyrus |  | 15.17 | 8 | -48 | 36 |
| L Precuneus |  | 11.71 | -4 | -68 | 36 |
| R Calcarine cortex | 631 | 18.29 | 11 | -75 | 14 |
| L Calcarine cortex |  | 16.76 | -6 | -82 | 6 |
| L Inferior temporal gyrus (incl. VWFA) | 254 | 17.45 | -46 | -60 | -8 |
| L Inferior occipital gyrus |  | 12.28 | -42 | -75 | -8 |
| R Superior frontal gyrus | 300 | 16.96 | 26 | 0 | 56 |
| R Middle frontal gyrus |  | 13.77 | 46 | 0 | 52 |
| R Precentral gyrus |  | 13.59 | 48 | 5 | 34 |
| R Superior frontal gyrus | 136 | 16.95 | 24 | 30 | 52 |
| R Middle frontal gyrus |  | 10.45 | 36 | 20 | 49 |
| L Thalamus | 49 | 15.94 | -12 | -15 | 6 |
| R Insula | 66 | 14.96 | 36 | -15 | 19 |
| R Rolandic operculum |  | 9.94 | 46 | -10 | 19 |
| L Superior frontal gyrus | 115 | 14.94 | -19 | 30 | 59 |
| L Middle frontal gyrus |  | 12.12 | -24 | 25 | 52 |
| L Lobule IV, V of vermis | 105 | 14.55 | -2 | -50 | -21 |
| L Lobule VIII of vermis |  | 13.34 | -2 | -60 | -28 |
| L Middle frontal gyrus | 37 | 14.35 | -39 | 15 | 46 |
| L Rolandic operculum | 86 | 13.44 | -46 | -20 | 19 |
| L Insula |  | 13.01 | -36 | -18 | 22 |
| L Supramarginal gyrus |  | 11.03 | -52 | -28 | 22 |
| L Lobule VI of cerebellar hemisphere | 93 | 13.00 | -32 | -70 | -21 |
| R Supramarginal gyrus | 76 | 12.53 | 44 | -35 | 44 |
| R Inferior parietal lobule |  | 9.55 | 34 | -45 | 42 |
| L Medial part of the superior frontal gyrus | 43 | 12.11 | -9 | 62 | 19 |
| L Superior frontal gyrus |  | 9.86 | -16 | 58 | 16 |
| R Medial orbital part of the superior frontal gyrus | 39 | 11.73 | 1 | 60 | -4 |
| L Medial part of the superior frontal gyrus |  | 10.22 | -9 | 55 | -1 |

Note. Local peaks from a contrast represent the effect of time (TP_0_ - TP_5_). X, y, and z are MNI coordinates.

**Table S4.** The main effect of time in the Tactile Braille Lexical Decision Task.

| *Region Label* | *Cluster extent* | *F* | *x* | *y* | *z* |
| --- | --- | --- | --- | --- | --- |
| L Angular gyrus | 938 | 55.97 | -42 | -58 | 26 |
| L Medial cingulate gyrus | 1423 | 54.14 | -14 | -42 | 36 |
| R Precuneus |  | 45.38 | 14 | -50 | 39 |
| L Precuneus |  | 33.32 | -2 | -55 | 36 |
| R Medial cingulate gyrus |  | 14.39 | 1 | -22 | 44 |
| R Inferior parietal lobule | 1666 | 50.56 | 44 | -35 | 46 |
| R Postcentral gyrus |  | 47.35 | 48 | -32 | 54 |
| R Superior occipital gyrus |  | 30.76 | 26 | -65 | 46 |
| R Superior parietal lobule |  | 29.84 | 26 | -60 | 59 |
| R Supramarginal gyrus |  | 18.57 | 58 | -18 | 29 |
| L Inferior parietal lobule | 4101 | 47.26 | -52 | -28 | 42 |
| L Postcentral gyrus |  | 42.52 | -44 | -32 | 44 |
| L Opercular part of the inferior frontal gyrus |  | 39.07 | -52 | 8 | 29 |
| L Superior parietal lobule |  | 36.51 | -34 | -52 | 62 |
| L Middle frontal gyrus |  | 33.98 | -26 | -8 | 52 |
| L Precentral gyrus |  | 33.79 | -56 | 0 | 44 |
| L Middle occipital gyrus |  | 28.79 | -29 | -72 | 32 |
| L Superior frontal gyrus |  | 27.99 | -32 | -8 | 66 |
| L Insula |  | 25.65 | -34 | 20 | 4 |
| R Superior frontal gyrus | 381 | 44.24 | 24 | -5 | 54 |
| R Middle frontal gyrus |  | 11.23 | 46 | 0 | 56 |
| R Angular gyrus | 851 | 42.58 | 54 | -62 | 26 |
| R Inferior parietal lobule |  | 29.91 | 56 | -58 | 39 |
| L Supplementary motor area | 800 | 41.29 | -4 | 2 | 56 |
| R Supplementary motor area |  | 30.00 | 4 | 15 | 49 |
| R Precentral gyrus | 606 | 31.01 | 54 | 8 | 36 |
| R Opercular part of the inferior frontal gyrus |  | 25.08 | 54 | 5 | 24 |
| R Insula |  | 23.35 | 34 | 20 | 9 |
| R Lobule VI of cerebellar hemisphere | 841 | 30.80 | 28 | -68 | -24 |
| R Lobule IV, V of vermis |  | 16.75 | 1 | -50 | -21 |
| R Crus II of cerebellar hemisphere |  | 13.16 | 6 | -78 | -34 |
| R Lobule IV, V of cerebellar hemisphere |  | 13.16 | 8 | -60 | -14 |
| R Lobule VI of vermis |  | 12.75 | 4 | -65 | -18 |
| R Lobule VIII of vermis |  | 11.47 | 1 | -70 | -38 |
| L Lobule VIII of vermis |  | 10.76 | -2 | -62 | -31 |
| L Thalamus | 136 | 29.79 | -14 | -15 | 9 |
| R Rolandic operculum | 55 | 27.98 | 41 | -15 | 22 |
| L Lobule VI of cerebellar hemisphere | 319 | 26.54 | -26 | -70 | -24 |
| L Middle frontal gyrus | 477 | 22.48 | -39 | 18 | 44 |
| L Superior frontal gyrus |  | 16.57 | -14 | 30 | 56 |
| R Thalamus | 136 | 21.46 | 11 | -12 | 4 |
| R Caudate |  | 11.72 | 11 | 10 | 2 |
| R Superior frontal gyrus | 264 | 20.66 | 16 | 30 | 56 |
| R Middle frontal gyrus |  | 14.67 | 38 | 20 | 52 |
| R Fusiform gyrus | 189 | 20.47 | 26 | -35 | -18 |
| L Inferior temporal gyrus (incl. VWFA) | 162 | 20.25 | -49 | -55 | -11 |
| R Inferior temporal gyrus | 49 | 18.49 | 54 | -55 | -11 |
| L Anterior cingulate gyrus | 240 | 17.46 | -12 | 50 | -1 |
| R Medial part of the superior frontal gyrus |  | 12.62 | 4 | 60 | 12 |
| R Inferior temporal gyrus | 210 | 16.72 | 44 | -5 | -36 |
| R Middle temporal gyrus |  | 14.28 | 58 | -2 | -16 |
| R Temporal pole (middle) |  | 12.71 | 48 | 8 | -28 |
| L Middle temporal gyrus | 56 | 16.20 | -46 | 5 | -31 |
| L Inferior temporal gyrus |  | 13.78 | -39 | 5 | -36 |
| L Putamen | 159 | 15.92 | -19 | 10 | 6 |
| L Caudate |  | 12.57 | -16 | 0 | 16 |
| L Middle temporal gyrus | 22 | 15.21 | -64 | -18 | -6 |
| L Hippocampus | 66 | 14.48 | -26 | -20 | -18 |
| L Fusiform gyrus |  | 13.65 | -26 | -32 | -21 |
| L Insula | 20 | 13.19 | -39 | -20 | 22 |

Note. Local peaks from a contrast represent the effect of time (TP_0_ - TP_5_) in a repeated measures ANOVA model. X, y, and z are MNI coordinates.

##

For each Braille Lexical Decision Task, whole-brain pairwise comparisons were performed between all pairs of TPs. As we did not have any hypothesis regarding the decrease in activity over time, we tested only comparisons in one direction (for example, TP_1_ > TP_0_ but not TP_0_ > TP_1_).

# 3 Visual Braille Lexical Decision Task - pairwise comparisons

**Table S5.** The comparison of TP_1_ > TP_0_ in the visual Braille Lexical Decision Task. All anatomical structures were labeled with the Automated Anatomical Labelling (AAL) atlas [(Rolls et al., 2020)](https://paperpile.com/c/XavzTt/10gsu). L = left, R = right.

| *Region Label* | *Cluster extent* | *t* | *x* | *y* | *z* |
| --- | --- | --- | --- | --- | --- |
| L Supplementary motor area | 583 | 10,90 | -6 | 5 | 59 |
| R Supplementary motor area |  | 8,99 | 4 | 15 | 49 |
| R Medial cingulate gyrus |  | 7,92 | 11 | 20 | 34 |
| L Precentral gyrus | 1219 | 10,43 | -46 | -2 | 36 |
| L Insula |  | 9,75 | -32 | 25 | 6 |
| L Opercular part of the inferior frontal gyrus |  | 9,61 | -49 | 8 | 24 |
| L Precentral gyrus |  | 9,17 | -49 | -5 | 52 |
| L Middle frontal gyrus |  | 8,66 | -26 | -2 | 49 |
| L Triangular part of the inferior frontal gyrus |  | 7,20 | -39 | 28 | 22 |
| R Insula | 302 | 8,84 | 34 | 28 | 2 |
| L Superior parietal lobule | 956 | 8,48 | -29 | -62 | 54 |
| L Inferior parietal lobule |  | 8,14 | -39 | -45 | 46 |
| L Middle occipital gyrus |  | 7,56 | -29 | -75 | 24 |
| L Superior occipital gyrus |  | 7,01 | -26 | -75 | 39 |
| R Superior frontal gyrus | 265 | 7,65 | 26 | 0 | 56 |
| R Precentral gyrus |  | 6,62 | 48 | 5 | 34 |
| R Superior parietal lobule | 331 | 7,54 | 21 | -65 | 56 |
| R Middle occipital gyrus |  | 5,99 | 31 | -75 | 29 |
| R Superior occipital gyrus |  | 5,83 | 26 | -65 | 39 |
| R Lobule VI of cerebellar hemisphere | 122 | 7,23 | 28 | -68 | -24 |
| R Supramarginal gyrus | 59 | 6,74 | 44 | -38 | 44 |
| L Inferior temporal gyrus | 97 | 6,44 | -46 | -62 | -8 |
| R Lobule VII of vermis | 41 | 6,02 | 6 | -75 | -26 |
| L Lobule VI of cerebellar hemisphere | 33 | 5,46 | -32 | -62 | -28 |

Note. Results thresholded using the FWE correction (p < 0.05) together with a cluster extent of 20 voxels and masked with significant voxels from the main effect of time. X, Y, and Z are MNI coordinates.


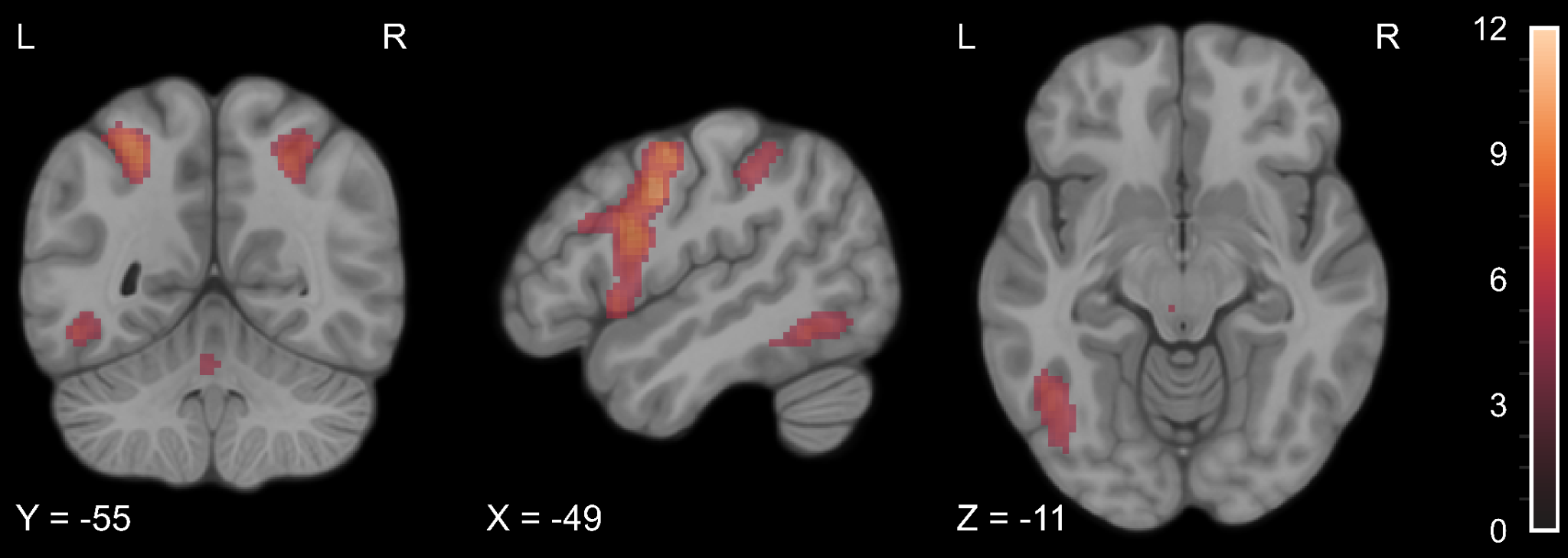


**Figure S1.** Statistical map of the paired t-test comparison of TP_1_ > TP_0_ for experimental > control contrast in the visual Braille Lexical Decision Task. The colourmap represents *t-*statistic range. We used the same coordinates to visualize and facilitate comparison between images.

**Table S6.** The comparison of TP_2_ > TP_0_ in the visual Braille Lexical Decision Task. All anatomical structures were labeled with the Automated Anatomical Labelling (AAL) atlas [(Rolls et al., 2020)](https://paperpile.com/c/XavzTt/10gsu). L = left, R = right.

| *Region Label* | *Cluster extent* | *t* | *x* | *y* | *z* |
| --- | --- | --- | --- | --- | --- |
| L Superior parietal lobule | 1097 | 10,15 | -26 | -65 | 59 |
| L Superior occipital gyrus |  | 8,92 | -26 | -75 | 39 |
| L Inferior parietal lobule |  | 8,41 | -39 | -45 | 44 |
| L Middle occipital gyrus |  | 8,34 | -29 | -75 | 29 |
| L Inferior occipital gyrus |  | 6,04 | -36 | -90 | -8 |
| L Precentral gyrus | 791 | 10,13 | -46 | -2 | 36 |
| L Opercular part of the inferior frontal gyrus |  | 8,84 | -49 | 8 | 24 |
| L Supplementary motor area | 446 | 9,80 | -6 | 2 | 59 |
| R Supplementary motor area |  | 7,19 | 4 | 15 | 49 |
| R Medial cingulate gyrus |  | 6,43 | 8 | 18 | 42 |
| R Lobule VI of cerebellar hemisphere | 446 | 8,85 | 28 | -68 | -24 |
| L Lobule VI of cerebellar hemisphere |  | 7,31 | -9 | -75 | -16 |
| R Superior parietal lobule | 465 | 8,29 | 24 | -62 | 54 |
| R Superior occipital gyrus |  | 7,90 | 26 | -72 | 39 |
| R Inferior parietal lobule |  | 7,89 | 26 | -55 | 52 |
| R Middle occipital gyrus |  | 7,69 | 31 | -75 | 29 |
| L Insula | 209 | 7,91 | -29 | 28 | 4 |
| L Inferior temporal gyrus | 246 | 7,59 | -46 | -60 | -8 |
| L Inferior occipital gyrus |  | 6,33 | -42 | -75 | -8 |
| R Superior frontal gyrus | 219 | 7,09 | 24 | 0 | 56 |
| R Precentral gyrus |  | 6,13 | 44 | 0 | 49 |
| R Opercular part of the inferior frontal gyrus |  | 5,92 | 48 | 10 | 29 |
| L Lobule VIII of vermis | 88 | 6,76 | -2 | -60 | -34 |
| L Lobule IV, V of vermis |  | 6,68 | -2 | -48 | -21 |
| R Supramarginal gyrus | 69 | 6,21 | 44 | -35 | 44 |
| R Inferior parietal lobule |  | 6,19 | 34 | -48 | 42 |
| R Postcentral gyrus |  | 5,52 | 51 | -30 | 46 |
| L Calcarine cortex | 261 | 6,18 | -6 | -82 | 6 |
| R Calcarine cortex |  | 6,15 | 11 | -75 | 14 |
| R Cuneus |  | 5,77 | 1 | -88 | 19 |
| L Triangular part of the inferior frontal gyrus | 22 | 6,00 | -39 | 30 | 19 |
| L Crus I of cerebellar hemisphere | 50 | 5,96 | -32 | -62 | -31 |
| L Lobule VI of cerebellar hemisphere |  | 5,95 | -32 | -72 | -21 |

Note. Results thresholded using the FWE correction (p < 0.05) together with a cluster extent of 20 voxels and masked with significant voxels from the main effect of time. X, Y, and Z are MNI coordinates.


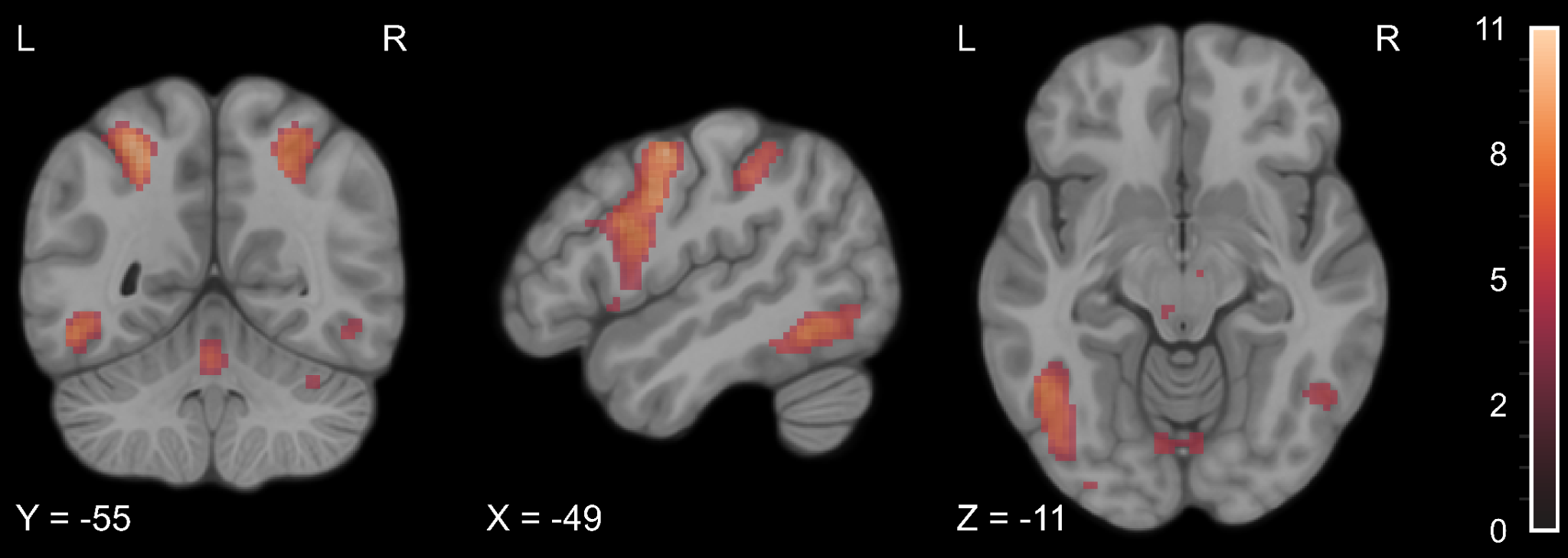


**Figure S2.** Statistical map of the paired t-test comparison of TP_2_ > TP_0_ for experimental > control contrast in the visual Braille Lexical Decision Task. The colourmap represents *t-*statistic range. We used the same coordinates to visualize and facilitate comparison between images.

**Table S7.** The comparison of TP_3_ > TP_0_ in the visual Braille Lexical Decision Task. All anatomical structures were labeled with the Automated Anatomical Labelling (AAL) atlas [(Rolls et al., 2020)](https://paperpile.com/c/XavzTt/10gsu). L = left, R = right.

| *Region Label* | *Cluster extent* | *t* | *x* | *y* | *z* |
| --- | --- | --- | --- | --- | --- |
| L Precentral gyrus | 1391 | 11,83 | -46 | -2 | 36 |
| L Opercular part of the inferior frontal gyrus |  | 9,97 | -49 | 8 | 24 |
| L Insula |  | 9,40 | -32 | 28 | 4 |
| L Triangular part of the inferior frontal gyrus |  | 7,33 | -39 | 28 | 22 |
| L Superior frontal gyrus |  | 6,60 | -22 | 5 | 62 |
| L Supplementary motor area | 598 | 11,11 | -4 | 2 | 62 |
| R Supplementary motor area |  | 8,66 | 6 | 12 | 49 |
| R Medial cingulate gyrus |  | 6,86 | 11 | 20 | 34 |
| R Insula | 282 | 7,13 | 36 | 18 | 6 |
| L Superior parietal lobule | 1169 | 8,77 | -26 | -62 | 56 |
| L Inferior parietal lobule |  | 8,77 | -26 | -55 | 52 |
| L Middle occipital gyrus |  | 8,61 | -29 | -75 | 24 |
| L Inferior occipital gyrus |  | 6,44 | -34 | -90 | -8 |
| R Lobule VI of cerebellar hemisphere | 373 | 8,58 | 28 | -68 | -24 |
| L Lobule VI of cerebellar hemisphere |  | 6,43 | -9 | -75 | -16 |
| L Crus I of cerebellar hemisphere |  | 6,29 | -4 | -75 | -26 |
| R Superior parietal lobule | 369 | 8,19 | 24 | -65 | 56 |
| R Middle occipital gyrus |  | 6,80 | 31 | -78 | 32 |
| R Inferior parietal lobule |  | 5,42 | 36 | -50 | 54 |
| L Inferior temporal gyrus | 227 | 7,72 | -49 | -55 | -11 |
| R Superior frontal gyrus | 282 | 7,47 | 24 | 0 | 54 |
| R Precentral gyrus |  | 7,20 | 48 | 5 | 34 |
| L Thalamus | 47 | 7,38 | -12 | -15 | 6 |
| R Calcarine cortex | 467 | 7,05 | 11 | -75 | 14 |
| L Calcarine cortex |  | 6,97 | -4 | -78 | 9 |
| L Lobule IV, V of vermis | 101 | 6,86 | -2 | -52 | -18 |
| R Lobule VIII of vermis |  | 6,70 | 1 | -60 | -26 |
| R Inferior parietal lobule | 68 | 6,32 | 44 | -35 | 46 |
| R Supramarginal gyrus |  | 6,29 | 51 | -30 | 44 |
| L Lobule VI of cerebellar hemisphere | 74 | 6,04 | -32 | -65 | -26 |

Note. Results thresholded using the FWE correction (p < 0.05) together with a cluster extent of 20 voxels and masked with significant voxels from the main effect of time. X, Y, and Z are MNI coordinates.


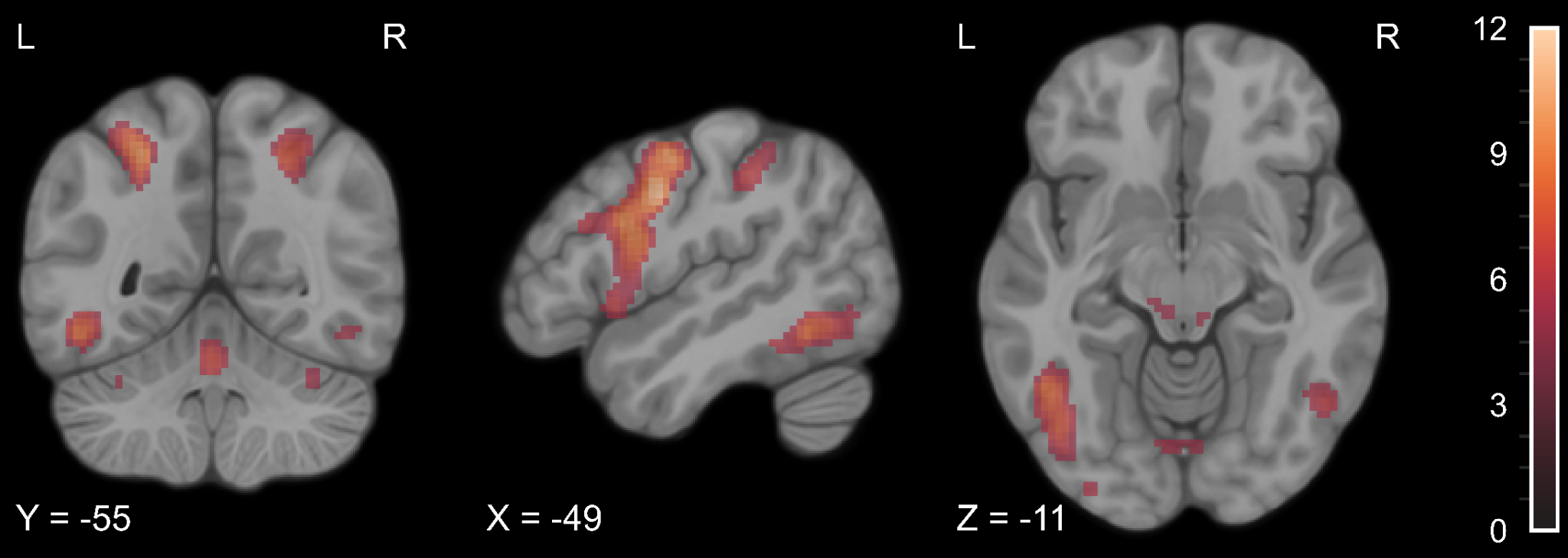


**Figure S3.** Statistical map of the paired t-test comparison of TP_3_ > TP_0_ for experimental > control contrast in the visual Braille Lexical Decision Task. The colourmap represents *t-*statistic range. We used the same coordinates to visualize and facilitate comparison between images.

**Table S8.** The comparison of TP_4_ > TP_0_ in the visual Braille Lexical Decision Task. All anatomical structures were labeled with the Automated Anatomical Labelling (AAL) atlas [(Rolls et al., 2020)](https://paperpile.com/c/XavzTt/10gsu). L = left, R = right.

| *Region Label* | *Cluster extent* | *t* | *x* | *y* | *z* |
| --- | --- | --- | --- | --- | --- |
| L Supplementary motor area | 548 | 11,73 | -4 | 2 | 62 |
| R Supplementary motor area |  | 7,40 | 6 | 12 | 49 |
| R Medial cingulate gyrus |  | 7,14 | 11 | 18 | 39 |
| L Precentral gyrus | 1301 | 11,53 | -46 | -2 | 36 |
| L Postcentral gyrus |  | 10,79 | -49 | -8 | 49 |
| L Opercular part of the inferior frontal gyrus |  | 9,72 | -49 | 8 | 26 |
| L Superior frontal gyrus |  | 8,58 | -26 | -5 | 59 |
| L Insula |  | 7,98 | -32 | 25 | 6 |
| L Triangular part of the inferior frontal gyrus |  | 5,38 | -39 | 28 | 22 |
| L Superior parietal lobule | 1131 | 10,41 | -26 | -62 | 56 |
| L Middle occipital gyrus |  | 9,49 | -29 | -75 | 24 |
| L Inferior parietal lobule |  | 7,51 | -39 | -42 | 46 |
| R Lobule VI of cerebellar hemisphere | 496 | 10,25 | 28 | -68 | -24 |
| L Lobule VI of cerebellar hemisphere |  | 8,25 | -9 | -75 | -16 |
| L Lobule VII of vermis |  | 7,29 | -2 | -75 | -26 |
| R Calcarine cortex | 631 | 8,69 | 18 | -68 | 6 |
| L Calcarine cortex |  | 8,12 | -4 | -78 | 9 |
| R Superior parietal lobule | 439 | 8,30 | 21 | -65 | 56 |
| R Middle occipital gyrus |  | 7,60 | 28 | -75 | 29 |
| R Superior occipital gyrus |  | 6,76 | 24 | -65 | 39 |
| R Insula | 289 | 7,58 | 36 | 18 | 9 |
| R Opercular part of the inferior frontal gyrus |  | 6,45 | 44 | 12 | 4 |
| L Inferior temporal gyrus | 254 | 7,85 | -44 | -42 | -16 |
| L Inferior occipital gyrus |  | 6,76 | -42 | -78 | -8 |
| L Thalamus | 49 | 7,76 | -12 | -18 | 6 |
| R Precentral gyrus | 270 | 7,70 | 51 | 0 | 49 |
| R Superior frontal gyrus |  | 6,79 | 26 | -2 | 56 |
| L Lobule IV, V of vermis | 105 | 7,67 | -2 | -50 | -21 |
| L Lobule VIII of vermis |  | 7,09 | -2 | -60 | -28 |
| L Lobule VI of cerebellar hemisphere | 92 | 7,40 | -32 | -70 | -21 |
| R Supramarginal gyrus | 56 | 6,34 | 44 | -35 | 44 |

Note. Results thresholded using the FWE correction (p < 0.05) together with a cluster extent of 20 voxels and masked with significant voxels from the main effect of time. X, Y, and Z are MNI coordinates.


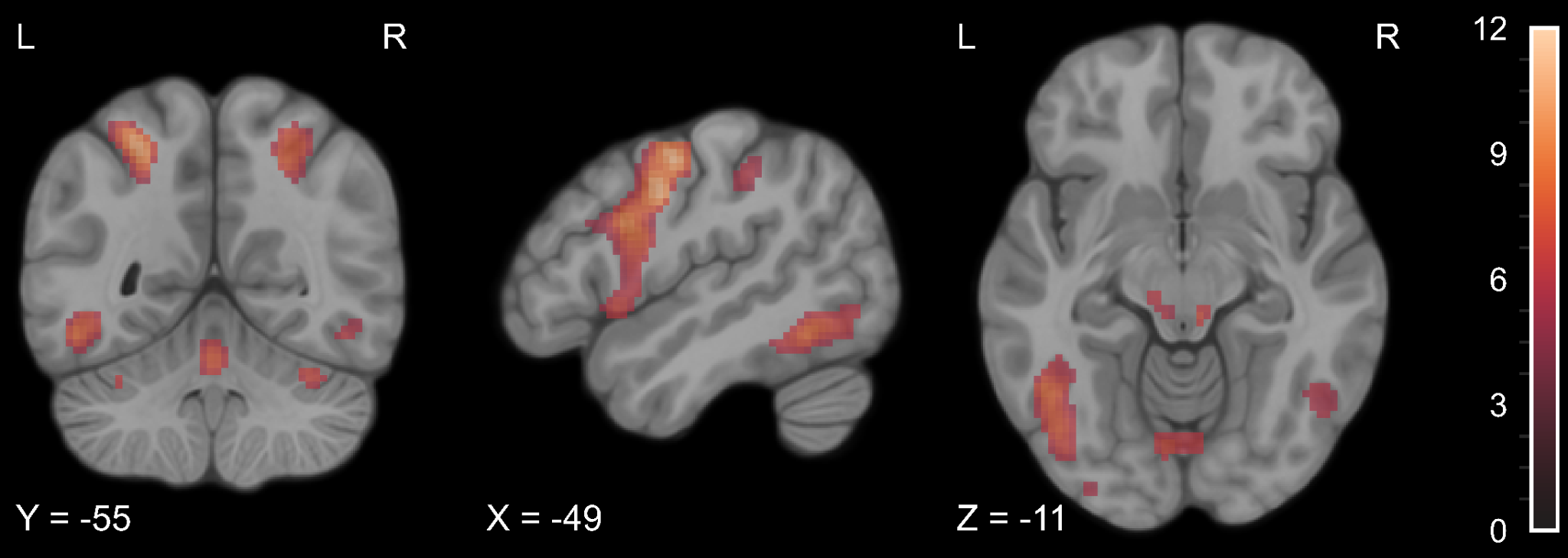


**Figure S4.** Statistical map of the paired t-test comparison of TP_4_ > TP_0_ for experimental > control contrast in the visual Braille Lexical Decision Task. The colourmap represents *t-*statistic range. We used the same coordinates to visualize and facilitate comparison between images.

**Table S9.** The comparison of TP_5_ > TP_0_ in the visual Braille Lexical Decision Task. All anatomical structures were labeled with the Automated Anatomical Labelling (AAL) atlas [(Rolls et al., 2020)](https://paperpile.com/c/XavzTt/10gsu). L = left, R = right.

| *Region Label* | *Cluster extent* | *t* | *x* | *y* | *z* |
| --- | --- | --- | --- | --- | --- |
| L Supplementary motor area | 486 | 11,64 | -4 | 2 | 59 |
| R Supplementary motor area |  | 7,07 | 6 | 12 | 49 |
| R Medial cingulate gyrus |  | 6,28 | 11 | 18 | 39 |
| L Precentral gyrus | 1212 | 11,21 | -54 | 0 | 46 |
| L Opercular part of the inferior frontal gyrus |  | 9,71 | -49 | 8 | 24 |
| L Insula |  | 7,89 | -32 | 28 | 4 |
| L Triangular part of the inferior frontal gyrus |  | 5,86 | -39 | 28 | 22 |
| L Superior parietal lobule | 992 | 9,33 | -26 | -62 | 56 |
| L Middle occipital gyrus |  | 8,04 | -29 | -75 | 24 |
| L Inferior parietal lobule |  | 7,40 | -39 | -42 | 46 |
| R Lobule VI of cerebellar hemisphere | 168 | 8,48 | 28 | -68 | -24 |
| R Superior occipital gyrus | 301 | 7,39 | 28 | -78 | 32 |
| R Superior parietal lobule |  | 7,35 | 21 | -65 | 56 |
| R Insula | 180 | 7,35 | 34 | 28 | 2 |
| R Calcarine cortex | 461 | 7,16 | 18 | -70 | 9 |
| L Calcarine cortex |  | 7,13 | -6 | -82 | 6 |
| L Thalamus | 42 | 6,78 | -12 | -15 | 9 |
| L Fusiform gyrus | 157 | 6,69 | -42 | -52 | -11 |
| L Inferior occipital gyrus |  | 5,67 | -42 | -78 | -8 |
| R Superior frontal gyrus | 56 | 6,47 | 26 | -2 | 54 |
| R Lobule VI of cerebellar hemisphere | 69 | 5,98 | 8 | -75 | -18 |
| R Lobule VII of vermis |  | 5,65 | 4 | -75 | -26 |
| R Precentral gyrus | 29 | 5,75 | 51 | 0 | 49 |

Note. Results thresholded using the FWE correction (p < 0.05) together with a cluster extent of 20 voxels and masked with significant voxels from the main effect of time. X, Y, and Z are MNI coordinates.

​​


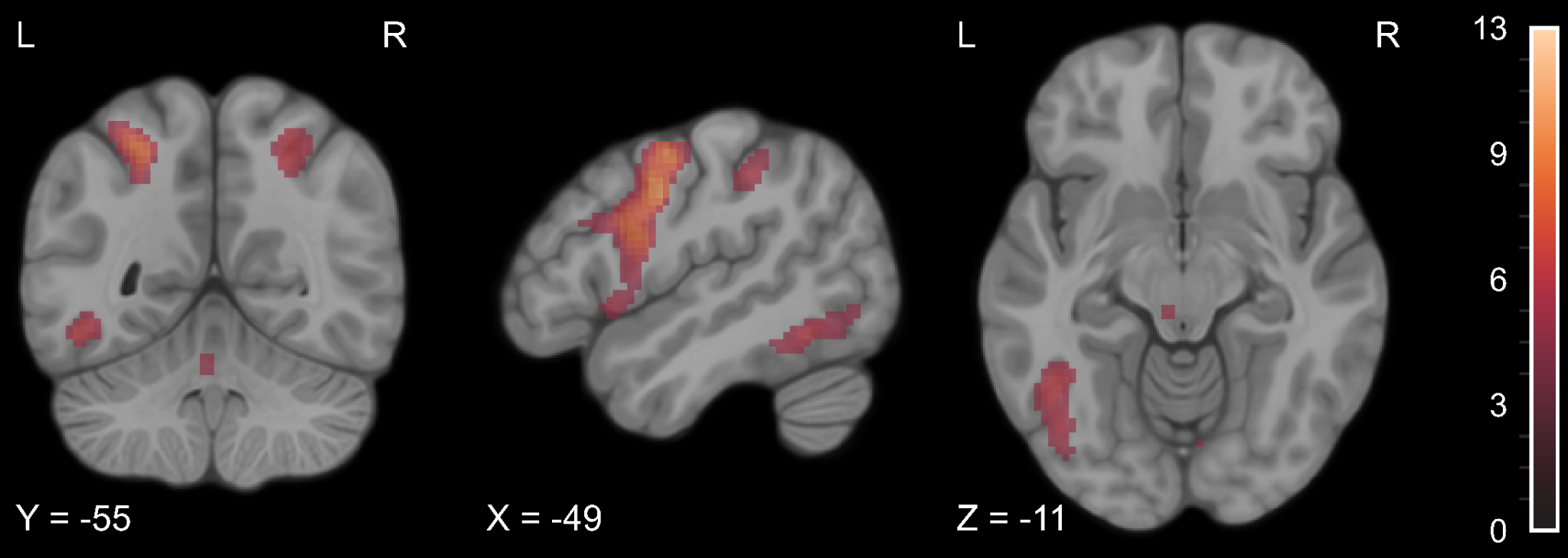


**Figure S5.** Statistical map of the paired t-test comparison of TP_5_ > TP_0_ for experimental > control contrast in the visual Braille Lexical Decision Task. The colourmap represents *t-*statistic range. We used the same coordinates to visualize and facilitate comparison between images.

## The comparison of all time points during learning and at follow-up

We computed a series of paired t-test comparisons for every two time points after the Braille course began (TP_2_ > TP_1_; TP_3_ > TP_1_; TP_4_ > TP_1_; TP_5_ > TP_1_; TP_3_ > TP_2_; TP_4_ > TP_2_; TP_5_ > TP_2_; TP_4_ > TP_3_; TP_5_ > TP_3_; TP_5_ > TP_4_). The analysis revealed no significant activity differences between any of the time points.

# 4 Tactile Braille Lexical Decision Task - pairwise comparisons

**Table S10.** The comparison of TP_1_ > TP_0_ in the tactile Braille Lexical Decision Task. All anatomical structures were labeled with the Automated Anatomical Labelling (AAL) atlas [(Rolls et al., 2020)](https://paperpile.com/c/XavzTt/10gsu). L = left, R = right.

| *Region Label* | *Cluster extent* | *t* | *x* | *y* | *z* |
| --- | --- | --- | --- | --- | --- |
| R Inferior parietal lobule | 1359 | 10,89 | 44 | -35 | 46 |
| R Postcentral gyrus |  | 10,70 | 48 | -32 | 54 |
| R Superior occipital gyrus |  | 8,70 | 31 | -70 | 44 |
| R Angular gyrus |  | 8,50 | 28 | -55 | 46 |
| R Superior parietal lobule |  | 7,83 | 26 | -60 | 62 |
| R Supramarginal gyrus |  | 7,01 | 58 | -15 | 29 |
| L Inferior parietal lobule | 1688 | 10,45 | -52 | -28 | 42 |
| L Postcentral gyrus |  | 10,17 | -44 | -32 | 44 |
| L Superior parietal lobule |  | 8,88 | -29 | -65 | 52 |
| L Superior occipital gyrus |  | 8,54 | -26 | -72 | 39 |
| L Middle occipital gyrus |  | 7,72 | -29 | -70 | 32 |
| L Supplementary motor area | 714 | 10,07 | -4 | 2 | 56 |
| R Supplementary motor area |  | 9,93 | 4 | 15 | 49 |
| R Medial cingulate gyrus |  | 5,97 | 8 | 20 | 32 |
| L Opercular part of the inferior frontal gyrus | 1192 | 9,73 | -52 | 10 | 29 |
| L Precentral gyrus |  | 9,08 | -46 | 0 | 36 |
| L Superior frontal gyrus |  | 7,76 | -32 | -8 | 66 |
| R Superior frontal gyrus | 304 | 9,62 | 26 | -5 | 54 |
| R Precentral gyrus | 327 | 8,20 | 54 | 5 | 39 |
| R Opercular part of the inferior frontal gyrus |  | 8,16 | 54 | 8 | 24 |
| L Insula | 259 | 8,11 | -34 | 20 | 4 |
| L Thalamus | 88 | 7,66 | -12 | -15 | 6 |
| R Lobule VI of cerebellar hemisphere | 116 | 7,63 | 28 | -70 | -24 |
| L Lobule VI of cerebellar hemisphere | 87 | 7,43 | -26 | -70 | -26 |
| R Thalamus | 51 | 6,53 | 11 | -12 | 4 |
| L Triangular part of the inferior frontal gyrus | 87 | 6,27 | -52 | 28 | 29 |
| L Putamen | 22 | 5,44 | -22 | -2 | 4 |

Note. Results thresholded using the FWE correction (p < 0.05) together with a cluster extent of 20 voxels and masked with significant voxels from the main effect of time. X, Y, and Z are MNI coordinates.


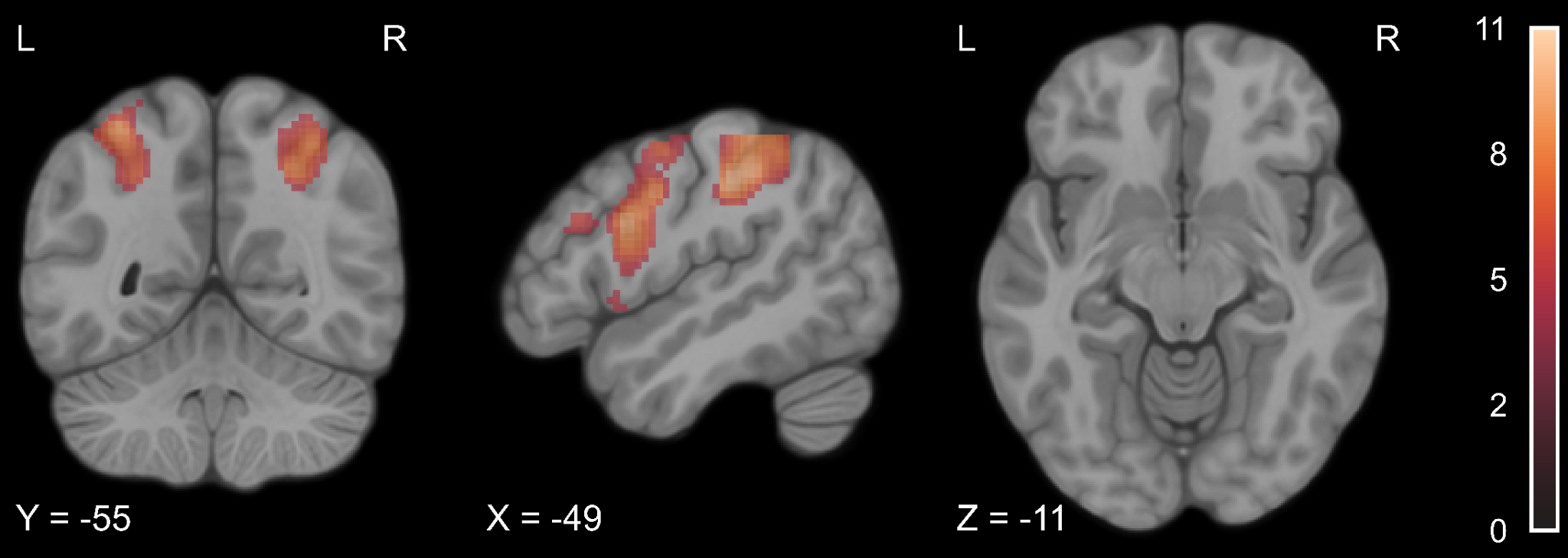


**Figure S6.** Statistical map of the paired t-test comparison of TP_1_ > TP_0_ for experimental > control contrast in the tactile Lexical Decision Task. The colourmap represents *t-*statistic range. We used the same coordinates to visualize and facilitate comparison between images.

**Table S11.** The comparison of TP_2_ > TP_0_ in the tactile Braille Lexical Decision Task. All anatomical structures were labeled with the Automated Anatomical Labelling (AAL) atlas [(Rolls et al., 2020)](https://paperpile.com/c/XavzTt/10gsu). L = left, R = right.

| *Region Label* | *Cluster extent* | *t* | *x* | *y* | *z* |
| --- | --- | --- | --- | --- | --- |
| R Supramarginal gyrus | 1665 | 13,33 | 41 | -35 | 44 |
| R Postcentral gyrus |  | 12,99 | 48 | -32 | 54 |
| R Inferior parietal lobule |  | 11,96 | 38 | -45 | 54 |
| R Superior occipital gyrus |  | 10,77 | 26 | -65 | 46 |
| R Superior parietal lobule |  | 10,66 | 26 | -60 | 59 |
| L Inferior parietal lobule | 4006 | 13,18 | -52 | -28 | 42 |
| L Postcentral gyrus |  | 12,60 | -42 | -32 | 44 |
| L Superior parietal lobule |  | 11,97 | -29 | -65 | 56 |
| L Opercular part of the inferior frontal gyrus |  | 11,16 | -52 | 10 | 29 |
| L Superior frontal gyrus |  | 10,85 | -26 | -8 | 59 |
| L Middle occipital gyrus |  | 10,33 | -29 | -72 | 32 |
| L Precentral gyrus |  | 10,16 | -54 | 0 | 46 |
| L Insula |  | 9,37 | -29 | 25 | 4 |
| L Superior frontal gyrus |  | 7,77 | -22 | 2 | 62 |
| L Triangular part of the inferior frontal gyrus |  | 6,17 | -42 | 28 | 24 |
| R Superior frontal gyrus | 369 | 12,46 | 24 | -5 | 54 |
| R Middle frontal gyrus |  | 5,96 | 48 | 2 | 54 |
| L Supplementary motor area | 750 | 11,23 | -6 | 5 | 54 |
| R Supplementary motor area |  | 9,55 | 4 | 15 | 49 |
| R Medial cingulate gyrus |  | 5,64 | 11 | 20 | 36 |
| R Lobule VI of cerebellar hemisphere | 840 | 10,69 | 28 | -68 | -24 |
| L Lobule IV, V of vermis |  | 8,14 | -2 | -50 | -21 |
| R Crus I of cerebellar hemisphere |  | 7,80 | 8 | -75 | -24 |
| R Crus II of cerebellar hemisphere |  | 7,19 | 6 | -78 | -31 |
| L Lobule VIII of vermis |  | 6,43 | -2 | -65 | -31 |
| R Lobule VI of vermis |  | 6,42 | 4 | -65 | -21 |
| R Lobule IV, V of cerebellar hemisphere |  | 6,42 | 8 | -60 | -11 |
| R Lobule VIII of vermis |  | 5,90 | 1 | -70 | -38 |
| L Lobule VI of cerebellar hemisphere | 319 | 9,88 | -26 | -70 | -24 |
| R Precentral gyrus | 560 | 9,41 | 54 | 8 | 36 |
| R Insula |  | 8,68 | 34 | 20 | 9 |
| R Opercular part of the inferior frontal gyrus |  | 8,26 | 54 | 5 | 22 |
| L Thalamus | 131 | 9,16 | -14 | -15 | 9 |
| L Inferior temporal gyrus | 162 | 8,41 | -49 | -55 | -11 |
| R Inferior temporal gyrus | 49 | 8,35 | 54 | -55 | -11 |
| R Thalamus | 125 | 8,20 | 14 | -12 | 6 |
| R Caudate |  | 6,23 | 14 | 2 | 14 |
| L Putamen | 152 | 7,26 | -22 | 10 | 6 |
| L Caudate |  | 6,50 | -16 | 0 | 16 |

Note. Results thresholded using the FWE correction (p < 0.05) together with a cluster extent of 20 voxels and masked with significant voxels from the main effect of time. X, Y, and Z are MNI coordinates.


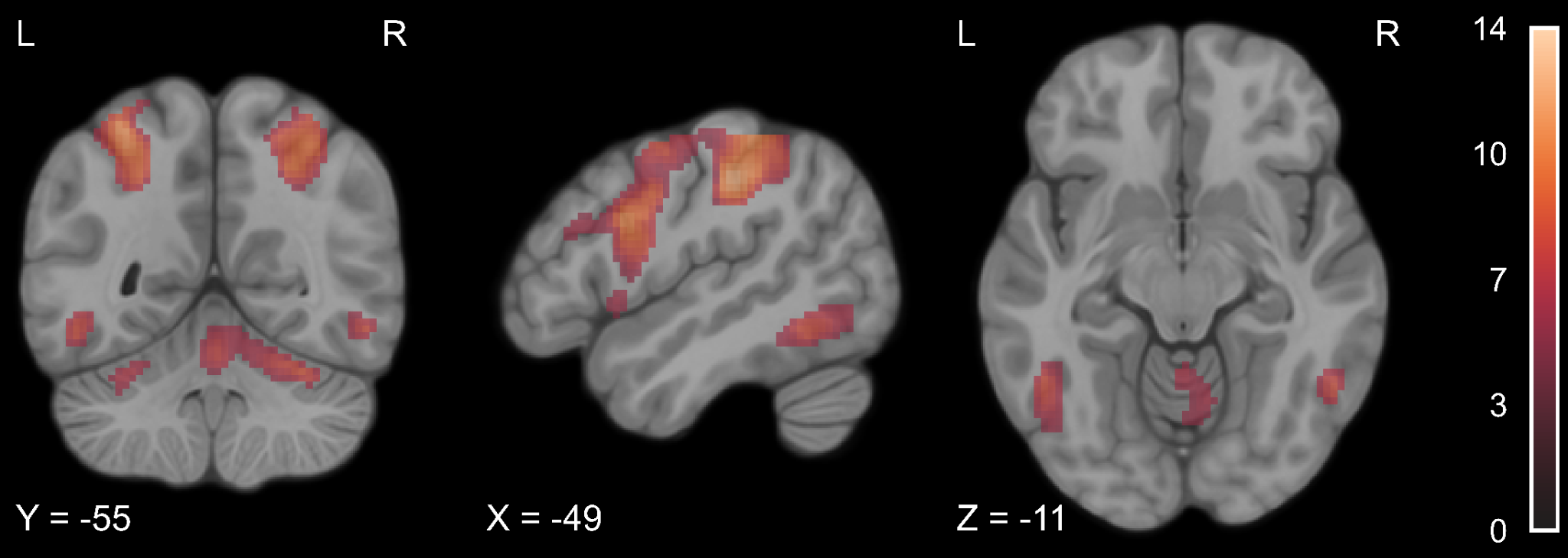


**Figure S7.** Statistical map of the paired t-test comparison of TP_2_ > TP_0_ for experimental > control contrast in the tactile Lexical Decision Task. The colourmap represents *t-*statistic range. We used the same coordinates to visualize and facilitate comparison between images.

**Table S12.** The comparison of TP_3_ > TP_0_ in the tactile Braille Lexical Decision Task. All anatomical structures were labeled with the Automated Anatomical Labelling (AAL) atlas [(Rolls et al., 2020)](https://paperpile.com/c/XavzTt/10gsu). L = left, R = right.

| *Region Label* | *Cluster extent* | *t* | *x* | *y* | *z* |
| --- | --- | --- | --- | --- | --- |
| R Postcentral gyrus | 1580 | 12,34 | 41 | -32 | 46 |
| R Inferior parietal lobule |  | 10,41 | 38 | -45 | 49 |
| R Superior occipital gyrus |  | 9,70 | 26 | -65 | 46 |
| R Angular gyrus |  | 8,98 | 28 | -55 | 46 |
| R Superior parietal lobule |  | 8,88 | 24 | -68 | 54 |
| R Supramarginal gyrus |  | 7,45 | 56 | -18 | 29 |
| L Opercular part of the inferior frontal gyrus | 3876 | 11,78 | -52 | 8 | 29 |
| L Inferior parietal lobule |  | 11,49 | -52 | -30 | 42 |
| L Precentral gyrus |  | 10,87 | -46 | -2 | 36 |
| L Superior parietal lobule |  | 10,18 | -22 | -65 | 42 |
| L Middle frontal gyrus |  | 10,00 | -26 | -8 | 52 |
| L Middle occipital gyrus |  | 9,28 | -29 | -72 | 32 |
| L Insula |  | 8,84 | -34 | 20 | 4 |
| L Triangular part of the inferior frontal gyrus |  | 8,12 | -34 | 30 | 2 |
| L Postcentral gyrus |  | 6,61 | -64 | -18 | 19 |
| R Superior frontal gyrus | 337 | 11,44 | 26 | -5 | 54 |
| R Middle frontal gyrus |  | 5,35 | 48 | 2 | 54 |
| L Supplementary motor area | 653 | 10,97 | -4 | 2 | 56 |
| R Supplementary motor area |  | 9,00 | 4 | 12 | 52 |
| R Medial cingulate gyrus |  | 5,54 | 8 | 20 | 32 |
| R Precentral gyrus | 373 | 10,15 | 54 | 8 | 36 |
| R Opercular part of the inferior frontal gyrus |  | 9,46 | 54 | 5 | 24 |
| L Thalamus | 126 | 10,02 | -12 | -15 | 6 |
| R Insula | 191 | 8,17 | 34 | 20 | 9 |
| R Lobule VI of cerebellar hemisphere | 514 | 8,90 | 28 | -70 | -24 |
| R Crus I of cerebellar hemisphere |  | 6,61 | 8 | -75 | -24 |
| R Crus II of cerebellar hemisphere |  | 6,46 | 6 | -78 | -34 |
| L Lobule IV, V of vermis |  | 6,27 | -2 | -50 | -21 |
| R Lobule VI of vermis |  | 5,85 | 4 | -65 | -21 |
| R Lobule VIII of vermis |  | 5,54 | 1 | -60 | -34 |
| R Lobule IV, V of cerebellar hemisphere |  | 5,39 | 8 | -60 | -11 |
| L Lobule VI of cerebellar hemisphere | 239 | 8,37 | -26 | -70 | -24 |
| L Crus II of cerebellar hemisphere |  | 5,94 | -6 | -78 | -28 |
| R Thalamus | 129 | 8,27 | 11 | -12 | 4 |
| R Caudate |  | 6,22 | 11 | 10 | 2 |
| L Inferior temporal gyrus | 143 | 7,95 | -49 | -52 | -11 |
| R Inferior temporal gyrus | 49 | 7,66 | 54 | -55 | -11 |
| L Putamen | 142 | 6,91 | -22 | 5 | 6 |
| L Caudate |  | 6,19 | -16 | 0 | 16 |

Note. Results thresholded using the FWE correction (p < 0.05) together with a cluster extent of 20 voxels and masked with significant voxels from the main effect of time. X, Y, and Z are MNI coordinates.


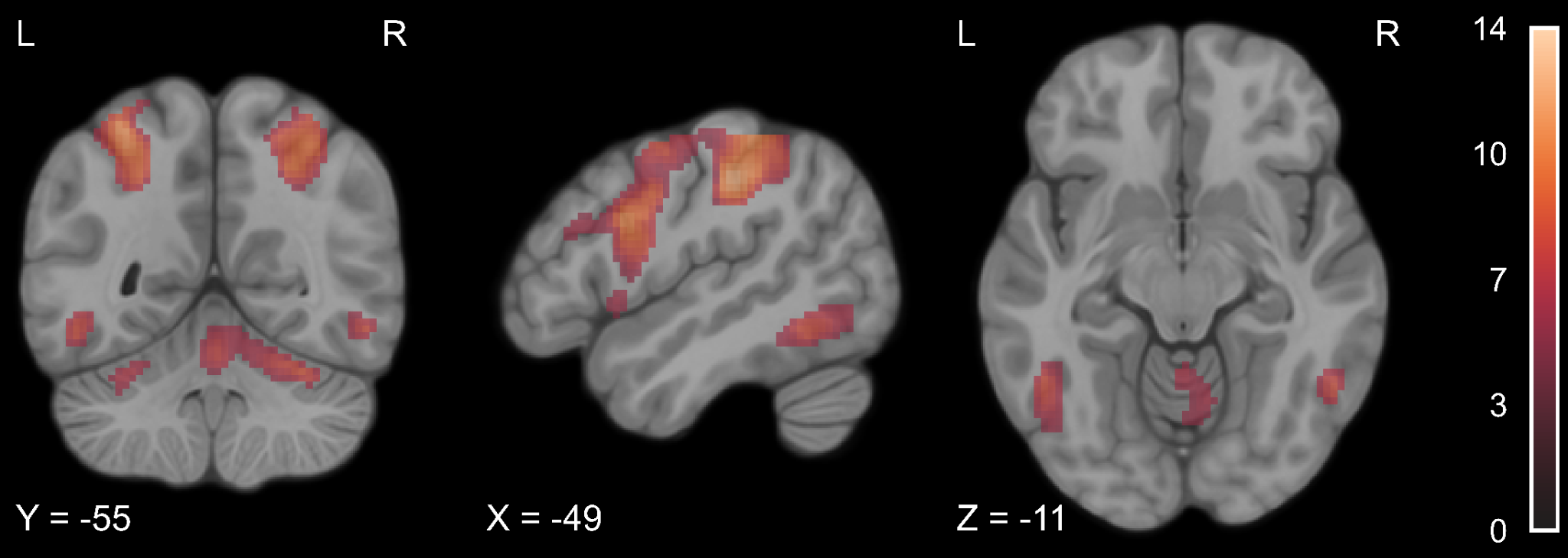


**Figure S8.** Statistical map of the paired t-test comparison of TP_3_ > TP_0_ for experimental > control contrast in the tactile Lexical Decision Task. The colourmap represents *t-*statistic range. We used the same coordinates to visualize and facilitate comparison between images.

**Table S13.** The comparison of TP_4_ > TP_0_ in the tactile Braille Lexical Decision Task. All anatomical structures were labeled with the Automated Anatomical Labelling (AAL) atlas [(Rolls et al., 2020)](https://paperpile.com/c/XavzTt/10gsu). L = left, R = right.

| *Region Label* | *Cluster extent* | *t* | *x* | *y* | *z* |
| --- | --- | --- | --- | --- | --- |
| R Inferior parietal lobule | 1633 | 12,98 | 44 | -35 | 46 |
| R Superior parietal lobule |  | 10,29 | 28 | -52 | 49 |
| R Angular gyrus |  | 9,82 | 26 | -62 | 46 |
| R Superior occipital gyrus |  | 8,97 | 28 | -70 | 44 |
| R Supramarginal gyrus |  | 7,84 | 56 | -18 | 29 |
| R Superior frontal gyrus | 380 | 12,50 | 26 | -5 | 54 |
| R Middle frontal gyrus |  | 6,90 | 46 | 0 | 56 |
| L Inferior parietal lobule | 3973 | 12,33 | -52 | -28 | 42 |
| L Postcentral gyrus |  | 11,26 | -44 | -32 | 44 |
| L Superior parietal lobule |  | 11,11 | -39 | -50 | 62 |
| L Opercular part of the inferior frontal gyrus |  | 10,91 | -52 | 10 | 29 |
| L Superior frontal gyrus |  | 10,58 | -26 | -8 | 59 |
| L Precentral gyrus |  | 10,56 | -56 | 0 | 44 |
| L Insula |  | 9,45 | -32 | 22 | 4 |
| L Middle occipital gyrus |  | 9,28 | -29 | -72 | 32 |
| L Supplementary motor area | 795 | 12,02 | -4 | 2 | 59 |
| R Supplementary motor area |  | 10,13 | 4 | 15 | 49 |
| R Medial cingulate gyrus |  | 6,72 | 11 | 15 | 39 |
| R Precentral gyrus | 603 | 10,58 | 54 | 8 | 36 |
| R Opercular part of the inferior frontal gyrus |  | 9,34 | 54 | 5 | 24 |
| R Insula |  | 9,27 | 34 | 22 | 6 |
| R Lobule VI of cerebellar hemisphere | 816 | 10,42 | 28 | -68 | -24 |
| R Crus I of cerebellar hemisphere |  | 7,42 | 38 | -72 | -26 |
| L Lobule IV, V of vermis |  | 7,30 | -2 | -52 | -24 |
| R Lobule IV, V of vermis |  | 6,73 | 6 | -62 | -14 |
| R Lobule VIII of vermis |  | 6,30 | 4 | -70 | -36 |
| L Thalamus | 136 | 10,41 | -14 | -15 | 9 |
| L Lobule VI of cerebellar hemisphere | 316 | 9,68 | -26 | -70 | -24 |
| R Thalamus | 136 | 9,22 | 11 | -12 | 4 |
| R Caudate |  | 6,67 | 11 | 10 | 2 |
| L Inferior temporal gyrus | 153 | 8,07 | -49 | -58 | -8 |
| L Putamen | 159 | 7,58 | -19 | 10 | 6 |
| L Caudate |  | 6,52 | -16 | 0 | 16 |
| R Inferior temporal gyrus | 46 | 7,47 | 54 | -58 | -11 |

Note. Results thresholded using the FWE correction (p < 0.05) together with a cluster extent of 20 voxels and masked with significant voxels from the main effect of time. X, Y, and Z are MNI coordinates.


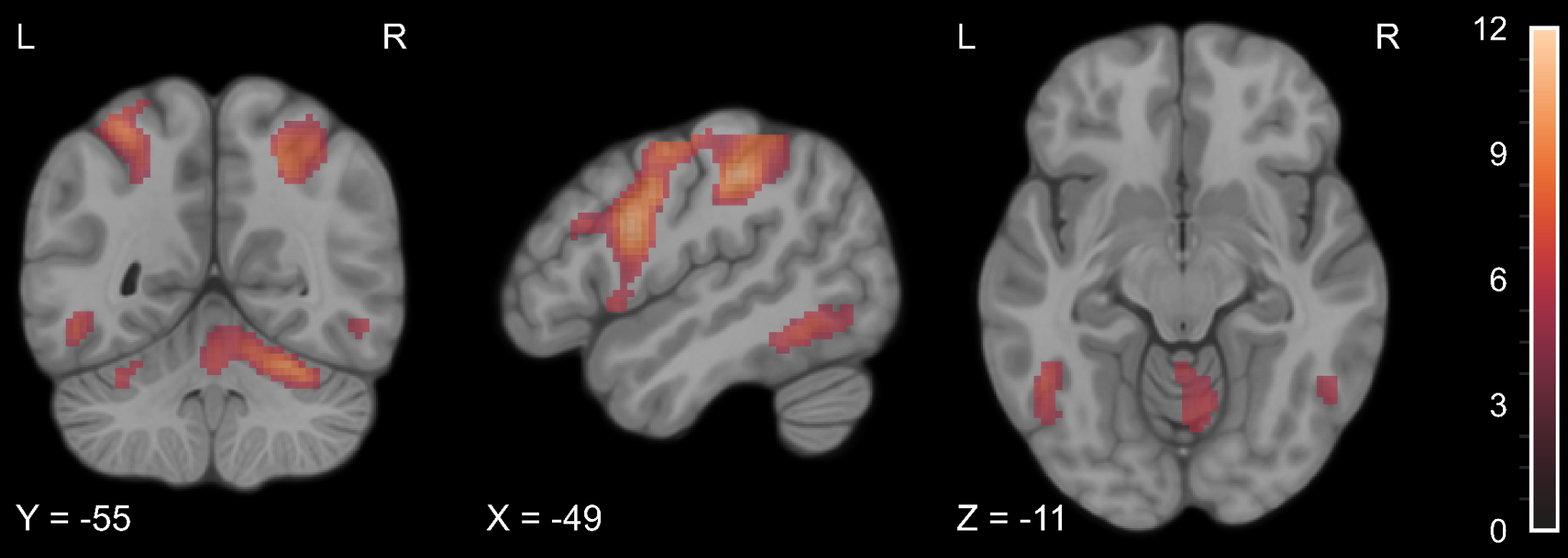


**Figure S9.** Statistical map of the paired t-test comparison of TP_4_ > TP_0_ for experimental > control contrast in the tactile Lexical Decision Task. The colourmap represents *t-*statistic range. We used the same coordinates to visualize and facilitate comparison between images.

**Table S14.** The comparison of TP_5_ > TP_0_ in the tactile Braille Lexical Decision Task. All anatomical structures were labeled with the Automated Anatomical Labelling (AAL) atlas [(Rolls et al., 2020)](https://paperpile.com/c/XavzTt/10gsu). L = left, R = right.

| *Region Label* | *Cluster extent* | *t* | *x* | *y* | *z* |
| --- | --- | --- | --- | --- | --- |
| L Supplementary motor area | 419 | 11,13 | -6 | 2 | 56 |
| R Supplementary motor area |  | 7,57 | 6 | 5 | 59 |
| R Postcentral gyrus | 1335 | 11,05 | 41 | -32 | 46 |
| R Inferior parietal lobule |  | 9,71 | 38 | -45 | 54 |
| R Superior parietal lobule |  | 8,31 | 28 | -52 | 49 |
| R Angular gyrus |  | 8,17 | 26 | -62 | 46 |
| R Superior occipital gyrus |  | 7,50 | 28 | -70 | 39 |
| R Supramarginal gyrus |  | 5,97 | 56 | -18 | 29 |
| L Inferior parietal lobule | 3212 | 10,91 | -52 | -28 | 42 |
| L Superior parietal lobule |  | 10,46 | -39 | -50 | 62 |
| L Opercular part of the inferior frontal gyrus |  | 10,28 | -52 | 8 | 29 |
| L Precentral gyrus |  | 10,27 | -56 | 0 | 44 |
| L Middle frontal gyrus |  | 10,05 | -26 | -8 | 52 |
| L Postcentral gyrus |  | 9,42 | -44 | -35 | 54 |
| L Middle occipital gyrus |  | 8,86 | -29 | -70 | 32 |
| L Supramarginal gyrus |  | 7,95 | -62 | -20 | 42 |
| R Superior frontal gyrus | 256 | 10,10 | 26 | -5 | 54 |
| L Thalamus | 94 | 8,76 | -12 | -15 | 6 |
| R Precentral gyrus | 191 | 8,66 | 54 | 8 | 36 |
| R Opercular part of the inferior frontal gyrus |  | 7,32 | 54 | 5 | 24 |
| R Lobule VI of cerebellar hemisphere | 245 | 8,38 | 26 | -68 | -24 |
| R Crus I of cerebellar hemisphere |  | 5,50 | 38 | -72 | -26 |
| L Inferior temporal gyrus | 79 | 7,38 | -49 | -55 | -14 |
| L Lobule VI of cerebellar hemisphere | 99 | 7,11 | -26 | -70 | -24 |
| L Insula | 154 | 7,11 | -32 | 22 | 4 |
| R Insula | 97 | 6,58 | 34 | 20 | 9 |
| R Thalamus | 35 | 6,55 | 11 | -12 | 4 |

Note. Results thresholded using the FWE correction (p < 0.05) together with a cluster extent of 20 voxels and masked with significant voxels from the main effect of time. X, Y, and Z are MNI coordinates.

​​


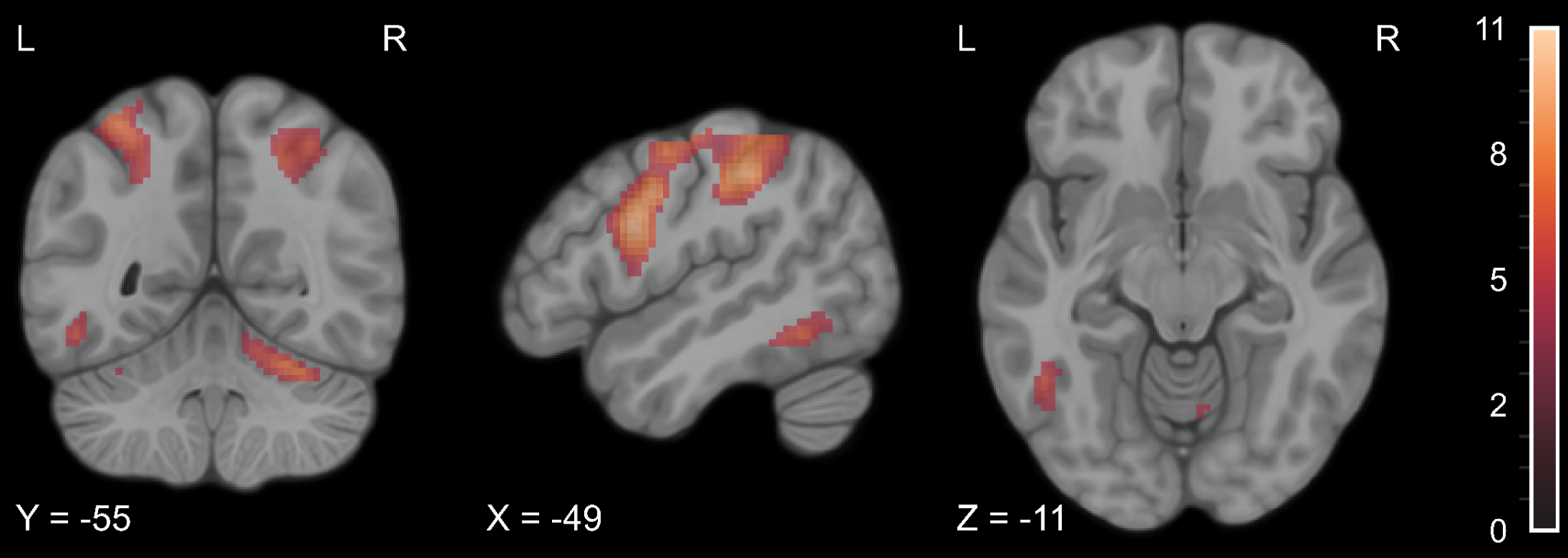


**Figure S10.** Statistical map of the paired t-test comparison of TP_5_ > TP_0_ for experimental > control contrast in the tactile Lexical Decision Task. The colourmap represents *t-*statistic range. We used the same coordinates to visualize and facilitate comparison between images.

## The comparison of all time points during learning and at follow-up

We computed a series of paired t-test comparisons for every two time points after the Braille course began (TP_2_ > TP_1_; TP_3_ > TP_1_; TP_4_ > TP_1_; TP_5_ > TP_1_; TP_3_ > TP_2_; TP_4_ > TP_2_; TP_5_ > TP_2_; TP_4_ > TP_3_; TP_5_ > TP_3_; TP_5_ > TP_4_). The analysis revealed no significant activity differences between any of the time points.

# 5 Braille-general and Modality-specific activations in the Lexical Decision Tasks

**Table S15.** The Braille-general activity in Lexical Decision Task.

| *Region Label* | *Cluster extent* | *t* | *x* | *y* | *z* |
| --- | --- | --- | --- | --- | --- |
| L Supplementary motor area | 10903 | 18.20 | -4 | 2 | 59 |
| R Lobule VI of cerebellar hemisphere |  | 17.15 | 31 | -70 | -26 |
| L Triangular part of the inferior frontal gyrus |  | 16.57 | -46 | 28 | 24 |
| L Precentral gyrus |  | 16.55 | -54 | 0 | 46 |
| L Inferior temporal gyrus |  | 15.47 | -49 | -60 | -8 |
| L Postcentral gyrus |  | 14.99 | -49 | -8 | 52 |
| L Opercular part of the inferior frontal gyrus |  | 14.19 | -49 | 12 | 2 |
| L Thalamus |  | 14.04 | -14 | -15 | 9 |
| R Lobule VI of cerebellar hemisphere |  | 13.77 | 14 | -75 | -24 |
| L Insula |  | 13.75 | -29 | 22 | 6 |
| L Lobule VI of cerebellar hemisphere |  | 13.30 | -26 | -70 | -26 |
| L Caudate |  | 13.23 | -16 | 5 | 14 |
| L Anterior cingulate gyrus |  | 12.99 | -6 | 5 | 29 |
| L Inferior parietal lobule | 2156 | 16.49 | -29 | -75 | 42 |
| L Superior parietal lobule |  | 13.59 | -26 | -65 | 62 |
| R Opercular part of the inferior frontal gyrus | 2025 | 14.63 | 51 | 8 | 26 |
| R Precentral gyrus |  | 13.86 | 54 | 12 | 36 |
| R Superior frontal gyrus |  | 11.99 | 24 | -2 | 54 |
| R Triangular part of the inferior frontal gyrus |  | 11.08 | 48 | 30 | 26 |
| R Middle frontal gyrus |  | 10.89 | 41 | 32 | 19 |
| R Insula |  | 9.13 | 34 | 18 | -6 |
| R Orbital part of the inferior frontal gyrus |  | 7.85 | 31 | 25 | -11 |
| R Putamen |  | 6.79 | 34 | 2 | -1 |
| R Inferior parietal lobule | 1710 | 14.45 | 38 | -45 | 49 |
| R Supramarginal gyrus |  | 13.93 | 44 | -35 | 42 |
| R Superior occipital gyrus |  | 13.37 | 28 | -65 | 36 |
| R Angular gyrus |  | 12.22 | 28 | -58 | 44 |
| R Superior parietal lobule |  | 11.71 | 21 | -68 | 54 |
| L Lobule VIII of cerebellar hemisphere | 40 | 8.80 | -14 | -62 | -48 |
| L Supramarginal gyrus | 70 | 7.62 | -62 | -42 | 24 |

Note. Local peaks from a contrast represent the main effect of condition in the visual and tactile Braille LDT. The voxels active in the Print LDT are excluded. X, y, and z are MNI coordinates.

**Table S16.** The activity specific for visual Braille reading in Lexical Decision Task.

| *Region Label* | *Cluster extent* | *t* | *x* | *y* | *z* |
| --- | --- | --- | --- | --- | --- |
| L Middle occipital gyrus | 5488 | 18.85 | -29 | -80 | 19 |
| R Middle occipital gyrus |  | 16.19 | 31 | -78 | 26 |
| L Inferior occipital gyrus |  | 16.07 | -36 | -88 | -6 |
| R Calcarine cortex |  | 15.79 | 14 | -70 | 12 |
| L Calcarine cortex |  | 15.08 | -2 | -78 | 9 |
| L Fusiform gyrus |  | 14.52 | -39 | -70 | -14 |
| R Superior occipital gyrus |  | 14.20 | 26 | -80 | 36 |
| R Inferior occipital gyrus |  | 13.96 | 44 | -65 | -11 |
| R Inferior temporal gyrus |  | 12.32 | 51 | -70 | -8 |
| R Superior frontal gyrus | 713 | 15.68 | 16 | 8 | 64 |
| R Middle frontal gyrus |  | 12.77 | 36 | 35 | 34 |
| R Precentral gyrus |  | 12.25 | 36 | 2 | 32 |
| R Supplementary motor area |  | 11.48 | 8 | 15 | 62 |
| R Precentral gyrus |  | 9.95 | 41 | -2 | 36 |
| R Triangular part of the inferior frontal gyrus |  | 9.83 | 36 | 28 | 26 |
| R Supplementary motor area |  | 8.55 | 6 | 22 | 64 |
| R Medial part of the superior frontal gyrus |  | 6.95 | 8 | 25 | 56 |
| R Opercular part of the inferior frontal gyrus | 152 | 12.27 | 41 | 12 | 4 |
| R Insula |  | 11.12 | 36 | 15 | 12 |
| R Orbital part of the inferior frontal gyrus |  | 8.16 | 48 | 20 | -8 |
| R Triangular part of the inferior frontal gyrus |  | 7.43 | 56 | 20 | 9 |
| R Lobule III of vermis | 531 | 8.55 | 6 | -38 | -18 |
| R Lobule IX of vermis |  | 7.26 | 1 | -52 | -34 |
| L Lobule III of vermis |  | 7.20 | -2 | -42 | -14 |
| L Lobule VIII of cerebellar hemisphere |  | 7.10 | -14 | -60 | -38 |
| L Lobule III of cerebellar hemisphere |  | 7.04 | -4 | -40 | -21 |
| R Lobule IX of cerebellar hemisphere |  | 7.03 | 4 | -45 | -44 |
| L Insula | 33 | 11.02 | -36 | 12 | 12 |
| L Opercular part of the inferior frontal gyrus |  | 5.31 | -44 | 8 | 12 |
| L Precentral gyrus | 26 | 10.52 | -39 | -10 | 44 |
| L Postcentral gyrus |  | 8.31 | -46 | -8 | 36 |
| R Medial cingulate gyrus | 197 | 10.39 | 8 | 35 | 34 |
| R Anterior cingulate gyrus |  | 8.75 | 11 | 38 | 24 |
| R Medial part of the superior frontal gyrus |  | 8.75 | 11 | 30 | 44 |
| L Anterior cingulate gyrus |  | 6.52 | -6 | 25 | 24 |
| L Superior frontal gyrus | 44 | 10.01 | -16 | 10 | 62 |
| L Middle frontal gyrus |  | 8.02 | -24 | 8 | 52 |
| L Crus II of cerebellar hemisphere | 25 | 9.62 | -39 | -62 | -46 |
| L Crus I of cerebellar hemisphere |  | 8.27 | -46 | -65 | -36 |
| L Middle frontal gyrus | 128 | 9.06 | -34 | 40 | 24 |
| L Triangular part of the inferior frontal gyrus |  | 8.73 | -36 | 32 | 26 |
| L Insula | 25 | 7.82 | -36 | 15 | -11 |
| R Caudate | 38 | 6.40 | 14 | 20 | 2 |

Note. Local peaks from a contrast represent the main effect of condition in the visual Braille LDT. The voxels active in other LDTs are excluded. X, y, and z are MNI coordinates.

**Table S17.** The activity specific for tactile reading in Lexical Decision Task.

| *Region Label* | *Cluster extent* | *t* | *x* | *y* | *z* |
| --- | --- | --- | --- | --- | --- |
| R Postcentral gyrus | 665 | 14.30 | 51 | -22 | 46 |
| R Supramarginal gyrus |  | 9.59 | 51 | -30 | 39 |
| R Superior parietal lobule |  | 8.17 | 24 | -58 | 66 |
| R Precentral gyrus |  | 6.51 | 56 | -8 | 46 |
| L Postcentral gyrus | 1596 | 13.66 | -46 | -35 | 56 |
| L Precentral gyrus |  | 13.46 | -44 | -12 | 59 |
| L Inferior parietal lobule |  | 13.44 | -52 | -28 | 46 |
| L Precentral gyrus |  | 11.01 | -29 | -12 | 59 |
| L Superior parietal lobule |  | 10.02 | -34 | -52 | 64 |
| L Supplementary motor area |  | 9.96 | -4 | -5 | 54 |
| L Supramarginal gyrus |  | 8.31 | -54 | -28 | 32 |
| R Supplementary motor area |  | 7.76 | 4 | -5 | 62 |
| L Superior frontal gyrus |  | 7.52 | -16 | -10 | 76 |
| R Posterior cingulate gyrus |  | 7.07 | 6 | -38 | 26 |
| L Medial cingulate gyrus |  | 6.99 | -4 | -8 | 32 |
| R Lobule VI of cerebellar hemisphere | 252 | 13.20 | 26 | -55 | -24 |
| R Lobule IV, V of vermis |  | 7.64 | 4 | -50 | -6 |
| R Superior frontal gyrus | 151 | 11.92 | 31 | -10 | 59 |
| L Putamen | 284 | 11.30 | -26 | -5 | 2 |
| L Insula |  | 8.51 | -39 | -2 | 9 |
| L Lobule VI of cerebellar hemisphere | 52 | 10.29 | -29 | -52 | -26 |
| L Fusiform gyrus |  | 5.69 | -32 | -40 | -24 |
| L Inferior temporal gyrus | 50 | 9.67 | -52 | -50 | -8 |
| L Triangular part of the inferior frontal gyrus | 54 | 8.89 | -46 | 45 | 2 |
| L Middle frontal gyrus |  | 6.93 | -39 | 50 | 6 |
| L Inferior parietal lobule | 48 | 8.76 | -34 | -72 | 39 |
| L Angular gyrus |  | 7.52 | -36 | -62 | 42 |
| L Thalamus | 52 | 8.75 | -16 | -25 | 4 |
| R Triangular part of the inferior frontal gyrus | 47 | 8.67 | 51 | 35 | 6 |
| R Middle frontal gyrus |  | 5.02 | 36 | 42 | 6 |
| R Precentral gyrus | 20 | 8.63 | 61 | 5 | 29 |
| R Rolandic operculum |  | 6.53 | 61 | 10 | 12 |
| R Putamen | 141 | 8.51 | 26 | 5 | 4 |
| R Pallidum |  | 7.34 | 24 | -5 | 6 |
| R Insula |  | 6.07 | 38 | 0 | 6 |
| R Orbital part of the middle frontal gyrus | 55 | 7.57 | 28 | 40 | -16 |
| R Crus I of cerebellar hemisphere | 39 | 7.35 | 16 | -78 | -28 |
| R Crus II of cerebellar hemisphere |  | 6.60 | 38 | -68 | -41 |
| L Medial part of the superior frontal gyrus | 27 | 6.83 | -6 | 35 | 34 |
| L Orbital part of the inferior frontal gyrus | 29 | 6.79 | -26 | 28 | -18 |

Note. Local peaks from a contrast represent the main effect of condition in the tactile Braille LDT. The voxels active in other LDTs are excluded. X, y, and z are MNI coordinates.

# 6 6-dots Detection Task

**Table S18.** The main effect of the group in the 6-dots Detection Task (experimental > control).

| *Region Label* | *Cluster extent* | *t* | *x* | *y* | *z* |
| --- | --- | --- | --- | --- | --- |
| R Postcentral gyrus | 327 | 16,91 | 66 | -12 | 24 |
| R Supramarginal gyrus |  | 9.21 | 61 | -22 | 32 |
| R Rolandic operculum |  | 7.04 | 51 | -22 | 22 |
| R Superior temporal gyrus |  | 6.81 | 64 | -30 | 19 |
| L Supramarginal gyrus | 1089 | 15.19 | -52 | -25 | 22 |
| L Precentral gyrus |  | 12.68 | -62 | 0 | 32 |
| L Postcentral gyrus |  | 12.20 | -62 | -15 | 34 |
| L Insula |  | 11.77 | -39 | -8 | 12 |
| L Triangular part of the inferior frontal gyrus |  | 10.03 | -49 | 25 | 26 |
| L Opercular part of the inferior frontal gyrus |  | 9.50 | -49 | 10 | 19 |
| L Inferior parietal lobule |  | 6.68 | -44 | -28 | 36 |
| L Putamen |  | 6.11 | -26 | -2 | -4 |
| L Superior parietal lobule | 1307 | 14.43 | -24 | -65 | 54 |
| L Paracentral lobule |  | 14.41 | -14 | -22 | 76 |
| L Postcentral gyrus |  | 13.98 | -22 | -25 | 72 |
| L Inferior parietal lobule |  | 12.94 | -32 | -68 | 42 |
| L Precentral gyrus |  | 8.07 | -22 | -28 | 56 |
| L Middle frontal gyrus |  | 5.42 | -24 | 8 | 54 |
| R Inferior parietal lobule | 473 | 11.59 | 38 | -52 | 54 |
| R Superior occipital gyrus |  | 8.82 | 26 | -68 | 39 |
| R Superior parietal lobule |  | 8.60 | 28 | -60 | 62 |
| R Angular gyrus |  | 8.02 | 28 | -55 | 46 |
| R Postcentral gyrus | 60 | 9.76 | 34 | -32 | 42 |
| R Supplementary motor area | 67 | 9.67 | 11 | 0 | 62 |
| R Superior frontal gyrus |  | 6.78 | 21 | -2 | 54 |
| R Lobule IV, V of cerebellar hemisphere | 663 | 9.39 | 8 | -50 | -8 |
| R Lobule VI of vermis |  | 7.84 | 4 | -62 | -24 |
| R Crus II of cerebellar hemisphere |  | 6.85 | 8 | -78 | -36 |
| R Fusiform gyrus |  | 6.74 | 31 | -52 | -18 |
| R Lobule VI of cerebellar hemisphere |  | 6.33 | 18 | -70 | -26 |
| L Lobule IV, V of cerebellar hemisphere |  | 5.10 | -4 | -52 | -21 |
| R Middle occipital gyrus | 73 | 9.23 | 41 | -78 | 12 |
| L Paracentral lobule | 107 | 7.94 | -4 | -20 | 62 |
| L Medial cingulate gyrus |  | 7.15 | -12 | -20 | 46 |
| R Insula | 47 | 7.81 | 38 | -8 | 16 |
| R Postcentral gyrus | 33 | 7.11 | 48 | -20 | 54 |
| L Inferior occipital gyrus | 50 | 7.02 | -49 | -62 | -14 |
| R Opercular part of the inferior frontal gyrus | 51 | 7.00 | 54 | 15 | 26 |
| R Triangular part of the inferior frontal gyrus |  | 5.49 | 46 | 18 | 22 |
| R Medial cingulate gyrus | 34 | 6.98 | 14 | -18 | 44 |
| L Middle frontal gyrus | 23 | 6.66 | -39 | 58 | 4 |
| L Orbital part of the middle frontal gyrus |  | 5.32 | -44 | 50 | -6 |
| L Middle frontal gyrus | 27 | 6.32 | -29 | 38 | 44 |
| R Superior frontal gyrus | 25 | 6.25 | 26 | 38 | 42 |
| R Superior temporal gyrus | 25 | 6.24 | 61 | -2 | 2 |
| L Thalamus | 22 | 5.99 | -9 | -25 | 2 |
| R Calcarine cortex | 86 | 5.98 | 1 | -58 | 14 |
| L Precuneus |  | 5.61 | -9 | -42 | 6 |
| R Precuneus |  | 5.24 | 4 | -68 | 26 |
| L Posterior cingulate gyrus |  | 5.04 | -4 | -48 | 19 |
| L Fusiform gyrus | 44 | 5.88 | -26 | -42 | -21 |
| L Lobule IV, V of cerebellar hemisphere |  | 5.87 | -22 | -38 | -26 |
| L Crus I of cerebellar hemisphere | 26 | 5.72 | -14 | -68 | -28 |
| R Caudate | 20 | 5.43 | 8 | 8 | -4 |

Note. Local peaks from a contrast represent the comparison of experimental > control groups. X, y, and z are MNI coordinates.

**Table S19.** The comparison of groups before the learning (TP_0_) in the 6-dots Detection Task (experimental > control).

| *Region Label* | *Cluster extent* | *t* | *x* | *y* | *z* |
| --- | --- | --- | --- | --- | --- |
| R Postcentral gyrus | 34 | 6.62 | 66 | -15 | 24 |
| R Supramarginal gyrus |  | 5.39 | 64 | -20 | 32 |

Note. Local peaks from a contrast represent the comparison of experimental > control groups at TP_0_. X, y, and z are MNI coordinates.

**Table S20.** The comparison of groups throughout the learning phase in the 6-dots Detection Task (experimental > control).

| *Region Label* | *Cluster extent* | *t* | *x* | *y* | *z* |
| --- | --- | --- | --- | --- | --- |
| R Postcentral gyrus | 210 | 14.81 | 66 | -12 | 24 |
| R Supramarginal gyrus |  | 7.85 | 64 | -22 | 32 |
| R Rolandic operculum |  | 6.11 | 51 | -22 | 22 |
| R Superior temporal gyrus |  | 5.77 | 64 | -30 | 22 |
| L Superior parietal lobule | 596 | 13.33 | -24 | -65 | 54 |
| L Inferior parietal lobule |  | 12.61 | -32 | -68 | 42 |
| L Postcentral gyrus |  | 6.22 | -49 | -30 | 56 |
| L Precentral gyrus | 461 | 13.21 | -22 | -25 | 69 |
| L Paracentral lobule |  | 12.67 | -16 | -22 | 76 |
| L Postcentral gyrus |  | 12.00 | -29 | -38 | 69 |
| L Supramarginal gyrus | 321 | 12.43 | -52 | -25 | 22 |
| L Precentral gyrus |  | 11.56 | -62 | 0 | 32 |
| L Postcentral gyrus |  | 10.92 | -62 | -15 | 34 |
| R Inferior parietal lobule | 420 | 10.50 | 38 | -50 | 54 |
| R Superior parietal lobule |  | 7.70 | 28 | -60 | 62 |
| R Superior occipital gyrus |  | 7.50 | 24 | -70 | 42 |
| R Angular gyrus |  | 6.58 | 31 | -62 | 39 |
| L Rolandic operculum | 85 | 9.76 | -36 | -8 | 14 |
| L Insula |  | 5.84 | -44 | -10 | 2 |
| R Middle occipital gyrus | 96 | 9.52 | 41 | -78 | 12 |
| L Triangular part of the inferior frontal gyrus | 359 | 9.50 | -49 | 25 | 26 |
| L Opercular part of the inferior frontal gyrus |  | 7.82 | -49 | 10 | 19 |
| R Lobule IV, V of cerebellar hemisphere | 574 | 9.28 | 8 | -50 | -8 |
| R Lobule VI of vermis |  | 7.53 | 4 | -62 | -24 |
| R Crus II of cerebellar hemisphere |  | 6.37 | 8 | -75 | -36 |
| R Fusiform gyrus |  | 5.86 | 31 | -52 | -18 |
| R Lobule VI of cerebellar hemisphere |  | 5.75 | 31 | -65 | -21 |
| R Supplementary motor area | 34 | 8.27 | 11 | 0 | 62 |
| R Superior frontal gyrus |  | 5.10 | 21 | -2 | 56 |
| R Postcentral gyrus | 44 | 7.86 | 34 | -32 | 42 |
| L Paracentral lobule | 73 | 7.60 | -4 | -20 | 62 |
| L Medial cingulate gyrus |  | 6.26 | -9 | -20 | 49 |
| R Postcentral gyrus | 32 | 7.09 | 31 | -38 | 62 |
| R Insula | 30 | 6.93 | 36 | -5 | 16 |
| L Precentral gyrus | 73 | 6.68 | -32 | 0 | 59 |
| L Middle frontal gyrus |  | 6.21 | -24 | 8 | 54 |
| R Opercular part of the inferior frontal gyrus | 39 | 6.43 | 54 | 15 | 26 |
| R Triangular part of the inferior frontal gyrus |  | 5.48 | 46 | 18 | 22 |
| L Inferior occipital gyrus (incl. VWFA) | 40 | 6.43 | -46 | -65 | -14 |
| R Superior frontal gyrus | 36 | 6.38 | 26 | 38 | 42 |
| R Medial cingulate gyrus | 26 | 6.04 | 14 | -18 | 44 |
| R Supplementary motor area |  | 5.33 | 11 | -15 | 52 |
| R Medial cingulate gyrus |  | 5.05 | 16 | -25 | 39 |
| L Crus I of cerebellar hemisphere | 20 | 5.89 | -14 | -68 | -28 |
| L Precuneus | 32 | 5.76 | -2 | -58 | 14 |
| L Precuneus | 25 | 5.49 | -9 | -42 | 6 |
| L Posterior cingulate gyrus |  | 5.39 | -6 | -45 | 19 |

Note. Local peaks from a contrast represent the comparison of experimental > control groups at TP_1_, TP_2_, TP_3,_ and TP_4_ all pooled together. X, y, and z are MNI coordinates.

# 6 Abbreviations

**Table S21.** Abbreviations used in the manuscript and their meanings.

| **Abbreviation** | **Meaning** |
| --- | --- |
| AAL | Automated Anatomical Labeling Atlas |
| ACG | Anterior Cingulate Gyrus |
| AMYG | Amygdala |
| ANG | Angular Gyrus |
| ANOVA | Analysis of Variance |
| BOLD | Blood-oxygen-level-dependent |
| CAL | Calcarine Cortex |
| CAU | Caudate |
| CER3 | Lobule III of cerebellar hemisphere |
| CER4 | Lobule IV of cerebellar hemisphere |
| CER5 | Lobule V of cerebellar hemisphere |
| CER6 | Lobule VI of cerebellar hemisphere |
| CER7B | Lobule VIIb of cerebellar hemisphere |
| CER8 | Lobule VIII of cerebellar hemisphere |
| CER9 | Lobule IX of cerebellar hemisphere |
| d | Cohen's d |
| DD6 | 6-dots Detection Task |
| EPI | Echo Planar Imaging |
| eta_p_^2^ | Partial eta-squared |
| F | Analysis of Variance F-test's value |
| FFG | Fusiform Gyrus |
| fMRI | Functional Magnetic Resonance Imaging |
| FWE | Family-Wise Error |
| FWHM | Full Width at Half Maximum |
| GLM | General Linear Model |
| IFG | Inferior Frontal Gyrus |
| IFGoperc | Pars opercularis of the Inferior Frontal Gyrus |
| IFGorb | Pars orbitalis of the Inferior Frontal Gyrus |
| IFGtriang | Pars triangularis of the Inferior Frontal Gyrus |
| INS | Insula |
| IOG | Inferior Occipital Gyrus |
| IPL | Inferior Parietal Lobule |
| ISI | Inter-stimulus interval |
| ITG | Inferior Temporal Gyrus |
| LDT | Lexical Decision Task |
| LING | Lingual Gyrus |
| M | Mean |
| MCG | Middle Cingulate Gyrus |
| MFG | Middle Frontal Gyrus |
| MOG | Middle Occipital Gyrus |
| MTG | Middle Temporal Gyrus |
| p | p-value |
| PAL | Pallidium |
| PCG | Posterior Cingulate Gyrus |
| PCL | Paracentral Lobule |
| PCUN | Precuneus |
| PoCG | Postcentral Gyrus |
| PreCG | Precentral Gyrus |
| PUT | Putamen |
| rmANOVA | Repeated measures Analysis of Variance |
| RO | Rolandic Operculum |
| SD | Standard Deviation |
| SFG | Superior Frontal Gyrus |
| SMA | Supplementary Motor Area |
| SMG | Supramarginal Gyrus |
| SPL | Superior Parietal Lobule |
| SPM | Statistical Parametric Mapping |
| STG | Superior Temporal Gyrus |
| t | Student's t-test value |
| T1w | T1-weighted image |
| THA | Thalamus |
| TP | Time point |
| TP-1 | Time point 1 week before training |
| TP0 | Training onset |
| TP1 | Time point after 1 week of training |
| TP2 | Time point after 6 weeks of training |
| TP3 | Time point after 3 months of training |
| TP4 | Time point after 7 months of traning (end of training) |
| TP5 | Time point after 10 months (after a 3-month-long break from learning) |
| VER | Vermis |
| VWFA | Visual Word Form Area |
| VWFA* | Visual Word Form Area's anatomical equivalent in the right hemisphere |
